# Dysregulation of transcripts SMAD4-209 and SMAD4-213 and their respective promoters in colon cancer cell lines

**DOI:** 10.7150/jca.98911

**Published:** 2024-08-06

**Authors:** Tamara Babic, Milena Ugrin, Sanja Jeremic, Milan Kojic, Jelena Dinic, Bojana Banovic Djeri, Jerome Zoidakis, Aleksandra Nikolic

**Affiliations:** 1 Institute of Molecular Genetics and Genetic Engineering, University of Belgrade, 11042 Belgrade, Serbia; 2 Institute of Virology, Vaccines and Sera “Torlak”, 11152 Belgrade, Serbia; 3 Institute for Biological Research “Siniša Stanković” — National Institute of the Republic of Serbia, University of Belgrade, 11060 Belgrade, Serbia; 4 Department of Biology, National and Kapodistrian University of Athens, 15701 Athens, Greece; 5 Proteomics Laboratory, Biomedical Research Foundation, Academy of Athens, 11527 Athens, Greece

**Keywords:** SMAD4, transcriptional regulation, alternative promoter, colorectal cancer, transcript

## Abstract

**Background:** The pervasive role of alternative promoters in context-specific isoform expression and the importance of promoter choice over its level of transcriptional activity have been recently implied based on pan-cancer *in silico* studies. We aimed to explore this phenomenon at the cellular level on the example of a major tumor suppressor SMAD4 in search of molecular mechanisms in colorectal cancer that could be exploited for novel biomarkers or therapeutic approaches.

**Methods:** Multi-omics technologies, *in silico* tools and *in vitro* functional assays were applied to analyze the transcripts expression and the alternative promoters' function of the SMAD4 gene in colon cell lines HCEC-1CT, HCT116, DLD-1, SW480 and SW620.

**Results:** High expression of the transcript SMAD4-213 emerged as a hallmark of colon cancer cells, while *in silico* tools point to its possible additional role and potential for sponging miRNAs. Based on the observed dysregulation of SMAD4-209 and SMAD4-213 in malignant vs. non-malignant colon cells, we propose that their expression ratio might be a solid biomarker candidate for colorectal cancer detection.

**Conclusions:** A differential pattern of the respective promoters' activity was observed that corresponds to the expression of transcripts, confirming the role of alternative promoters in context-specific isoform expression. The investigated *SMAD4* promoters and transcripts harbor translational potential that should be further investigated.

## Introduction

SMAD family member 4 (SMAD4) is a key mediator of the canonical transforming growth factor-beta (TGF-β) signaling pathway and has a major role in maintaining tissue homeostasis and cellular stability during the cell cycle. SMAD4 transduces signals from the cell membrane to the nucleus by binding to the SMAD-binding DNA element, acting as a transcriptional regulator of its target genes 1. Functional genetic alterations in the *SMAD4* coding region occur in about 30% of colorectal cancer (CRC) patients and it includes loss of heterozygosity which is often followed by deletion of the other allele or intragenic inactivating mutation, which eventually leads to decreased expression level or total loss of SMAD4 protein 2. Many studies showed that not only complete loss of SMAD4 is associated with tumor progression, but also decreased *SMAD4* expression level is an important factor in adverse disease prognosis and resistance to chemotherapy 3-5.

Transcriptional regulation of a gene is a multi-level and highly coordinated molecular mechanism that gives rise to transcriptome and proteome complexity. Based on the current genome assembly GRCh38, the human genome annotates 19,827 coding genes that give rise to 252,974 transcripts (Ensembl 109) 6. Like many other aspects of cellular dysregulation during cancer development and progression, several transcriptional regulatory mechanisms have been proven to be altered in colorectal cancer. A tumor-specific profile of transcripts with alternative transcription start sites (TSSs) was revealed in an exon array analysis in CRC samples, pointing to a role of aberrant mRNA and non-coding RNA expression patterns in carcinogenesis 7. Alternative gene promoters that drive transcription of multiple transcripts add even more complexity to the transcriptome and its' aberrant use was shown in several diseases including cancer 8. The pan-cancer analysis found that alternative promoter activity in tumors, including CRC, provides a more accurate predictor of patient survival than gene expression, once more pointing to the importance of a specific set of tumor-related transcripts instead of overall gene expression level 9. The choice of the alternative promoter that will be active in a cell-specific context is mediated by several mechanisms, including diverse promoter structure, epigenetic modifications and differential binding of transcription factors and regulators.

*SMAD4* gene transcriptional regulation, including alternative transcription initiation, splicing, polyadenylation and translation initiation, remains another yet unexplored mechanism that could be involved in the onset and progression of colorectal cancer. *SMAD4* spans 56652 bp of chromosome 18 from 51028394 to 51085045 on the forward strand. Its complex regulatory region is spanning over 80 kb and contains four segments with a promoter activity (A, B, C and D) 10. Two of them, C and D, have been confirmed earlier to be active in different cell types 11, 12. According to Ensembl 109 database, there are 24 alternative *SMAD4* transcripts that differ in their coding, 3'UTR or 5'UTR regions, as a consequence of different transcriptional regulatory mechanisms. According to the RNA Annotation and Mapping of Promoters for the Analysis of Gene Expression (RAMPAGE) data from the project Encyclopedia of DNA Elements (ENCODE), the major contributor to the SMAD4 protein expression in most tissues is transcript SMAD4-201 (ENST00000342988.8) 13. Our recent study revealed an abundance of SMAD4-201 transcript below 50% and variable expression of total RNA transcribed from the *SMAD4* gene in CRC cell lines and tissue samples, implying the relevance of other than SMAD4-201 transcript isoforms 14. The pervasive role of alternative promoters in context-specific isoform expression and the importance of promoter choice over its level of transcriptional activity have been recently implied based on pan-cancer *in silico* studies. Thus, we aimed to explore this phenomenon at the cellular level on the example of a major tumor suppressor *SMAD4* in search of molecular mechanisms in colorectal cancer that could be exploited for novel biomarkers or therapeutic approaches.

## Materials and Methods

### Cell cultures

Several human immortalized cell lines originating from the colon tissue were used in this study: immortalized epithelial cell line HCEC-1CT (CVCL_AQ45) (Evercyte GmbH, Austria) and malignant cell lines SW480 (CVCL_0546), SW620 (CVCL_0547), HCT116 (CVCL_0291) and DLD-1 (CVCL_0248) (ATCC, USA). All cell lines were maintained at 37°C and 5% CO2 in Dulbecco′s Modified Eagle′s - Medium (DMEM) (Capricorn Scientific, Germany) supplemented with 10% fetal bovine serum (FBS) (Capricorn Scientific, Germany) and 1% antibiotic/antimycotic solution (Capricorn Scientific, Germany).

Additionally, HCEC-1CT, HCT116 and DLD-1 cells were cultivated in 3D. For the generation of spheroids, adherent cells were detached with 1x trypsin/EDTA (Capricorn Scientific, Germany) and counted using a standard hemocytometer. Approximately 2x10^5^ cells/well were seeded in 24-well plate Nunclon™ Sphera™ Dish (Thermo Fisher Scientific, USA), specified as the low cell attachment surface, containing 1 mL of complete culture medium described above. Spheroids were cultured for 7 days in a humidified incubator at 37°C with 5% CO2. The collection of compact spheroids, without cell debris, was performed under the microscope to enable picking up only live cells for further extraction of total RNA.

### RNA sequencing

Total RNA was isolated from HCEC-1CT, HCT116 and DLD-1 cell spheroids from two independent 24-well plates per cell line to ensure biological replication and consistency using PureLink™ RNA Mini Kit (Thermo Fisher Scientific, USA) according to the manufacturer's protocol. Following RNA extraction, multiple replicates for each cell line were pooled and analyzed together to improve the sensitivity of downstream analyses, allowing for more accurate detection and quantification of low-abundance transcripts and providing robustness, ensuring that the final data is reflective of the general trend across all replicates. The concentration and purity of total RNA were evaluated by measuring the absorbance at 260 nm and 280 nm on the BioSpec-nano spectrophotometer (Shimadzu Corporation, Japan).

High-throughput next-generation RNA sequencing was performed by Novogene (UK) Company Limited (Cambridge, United Kingdom). Total RNA was subjected to sample quality check using 1% agarose gel electrophoresis, NanoDrop (Thermo Fisher Scientific, USA) reading to check for RNA amount and purity, and Agilent2100 (Agilent, USA) to check for RNA Integrity Number. Library preparation included ribosomal RNA depletion which enabled RNA enrichment for gene expression profiling for both coding and non-coding transcripts. Paired-end sequencing (2x150 bp) was performed on Illumina's NovaSeq6000 platform (Illumina, Inc., USA). Bioinformatics analysis included quality control (QC), removal of reads containing adapters, reads containing ambiguous nucleotides N>0.1% and reads with low quality (>50% bases showed QC<=5), mapping of clean reads to the reference genome version GRCh.38.p14 using HISAT2.2.1 and quantification of gene/transcript expression level using Novogene's well-established pipeline which resulted in FPKM (Fragments Per Kilobase of transcript sequence per Millions base pairs) values calculated for each transcript 15.

For differential gene/transcript expression analysis, edgeR4.2.0 software was used to analyze the significance of expression differences in the samples without biological replicates 16. Prior to differential gene expression analysis, for each sequenced library, the read counts were adjusted by the edgeR program through one scaling normalized factor and differential expression analysis of two conditions was performed using the edgeR R package. The P values were adjusted using the Benjamini and Hochberg methods. The corrected p-value of 0.05 was set as the threshold for significantly differential expression. A differentially expressed transcript was considered relevant if |logFC|>2.5 was in the same direction in both malignant cell lines vs. in non-malignant with p<0.05.

### *In silico* analysis of SMAD4-213 transcripts

The sequence of the SMAD4-213 (ENST00000592186.5) transcript was obtained as a FASTA file using the human GRCh38 assembly in the Ensembl genome browser 6.

The coding potential of the transcript was evaluated with the Coding Potential Calculator 2 tool 17. The 3D structure prediction of protein translated from SMAD4-213 was obtained using the UniProt database 18. Subcellular localization was analyzed with the AnnoLnc2 and LncLocator tools 19, 20. Transcriptional regulation of SMAD4-213 was analyzed with AnnoLnc2 and transcriptional regulators which are expressed only in colorectal carcinoma and have a predicted binding site upstream of/overlapping TSS were considered significant. RNA-binding proteins (RBPs) site prediction for SMAD4-213 was analyzed using RBPsuite using a general model 21. RNA-binding proteins with a score >0.9 and a verified motif file from the MEME-fimo tool were considered significant. The STRING database was used to assess protein-protein interactions (PPIs) in functional protein association networks using predicted RBPs as query proteins and only previously confirmed interactions in databases, experiments, or textmined from publications were chosen in settings parameters 22. Interactions between the SMAD4-213 and miRNA molecules were predicted using miRDB and AnnoLnc2 bioinformatics tools 19, 23. Only the miRNAs predicted to bind with a target score >85 in miRDB and miRNA which have a conservation score of 0.17 in primates and are supported by CLIP-seq in AnnoLnc2 were taken into consideration.

### Reporter vectors construction

Genomic DNA was isolated from the HCEC-1CT cell line using PureLink™ Genomic DNA Mini Kit (Thermo Fisher Scientific, USA) according to the manufacturer's protocol and used as a template for amplification of C and D promoter regions.

The amplification of 1197 bp long promoter C was performed using the following primers: 5'-GCGCGCTATAGAATTCCGAGTGTAAACACCTCTGGGGC-3' and 5'-CCGCCGAATTGGATCCCCGTCCGAGTTTAACTTGATTC-3'. Underlined nucleotides represent the restriction sites that were incorporated, *EcoRI* and *BamHI* respectively. The reaction mixture contained 200 ng of DNA, 1,5 U FIREPol DNA Polymerase (Solis BioDyne, Estonia), 1xReaction Buffer B (Solis BioDyne, Estonia), 2mM MgCl2 (Solis BioDyne, Estonia), 10% DMSO (Sigma-Aldrich, USA), 0.2 mM each dNTP (Thermo Fisher Scientific, USA) and 3 pmol of each primer. The amplification was performed on GeneAmp® PCR System 2700 (Applied Biosystems, USA) under the following conditions: initial denaturation at 95°C for 5 min, 30 cycles consisting of denaturation at 95°C for 30 s, annealing at 62°C for 45 s and polymerization at 72°C for 1 min, and final elongation at 72°C for 10 min.

The amplification of 1209 bp long promoter D was performed using the following primers: 5'-GCGCGCTATACTCGAGGCTGGGTCATATTTACTCTG-3' and 5'-CCGCCGAATTAAGCTTGCAAGTTTTTAAATCTGCCACC-3' Underlined nucleotides represent the restriction sites that were incorporated, *XhoI* and *HindIII* respectively. The reaction mixture contained 200 ng of DNA, 1,5 U Fast Gene Taq DNA Polymerase (NIPPON Genetics, Germany), 1xReaction Buffer A (NIPPON Genetics, Germany), 0.2 mM each dNTP (Thermo Fisher Scientific, USA) and 3 pmol of each primer. The amplification was performed on GeneAmp® PCR System 2700 (Applied Biosystems, USA) under the following conditions: initial denaturation at 95°C for 5 min, 25 cycles consisting of denaturation at 95°C for 30 s, annealing at 50°C for 30 s and polymerization at 72°C for 1 min, and final elongation at 72°C for 10 min.

The primary genetic structure of amplified promoter regions was confirmed by Sanger sequencing using the amplification primers.

Amplified promoter regions C and D were cloned into reporter vectors pZsGreen1-DR (Takara Bio, Japan) and pmCherry-1 (Takara Bio, Japan), respectively, by performing double digestion of vector and amplified fragment carrying promoter region with restriction enzymes *EcoRI* and *BamHI* for promoter C (Thermo Fisher Scientific, USA) and *XhoI* and *HindIII* (Thermo Fisher Scientific, USA) for promoter D, resulting in the formation of single-stranded sticky ends. Linearized vector and fragment carrying promoter region were ligated using T4 DNA Ligase (Thermo Fisher Scientific, USA). Ligated products were transformed in XL10-Gold Ultracompetent Cells (Agilent Technologies, USA). Reporter vector constructs were checked by restriction enzyme analysis and confirmed by Sanger sequencing. Plasmid DNA was isolated and prepared for transfection using Pure YieldTM Plasmid Miniprep System (Promega, USA).

### Transient transfection

Transfection of 1x10^5^ HCEC-1CT and 2x10^5^ SW620 cells was carried out in 6-well plates using Lipofectamine™ 3000 (Thermo Fisher Scientific, USA) according to the manufacturer's protocol. Combinations of empty vectors pZsGreen1-DR and pmCherry-1 or vectors with cloned promoter regions were added simultaneously in the same well to reduce experimental conditions variability. Cells were co-transfected with 1.5 μg of empty vectors or reporter constructs in Opti-MEM™ (Thermo Fisher Scientific, USA) for 4 hours, after which 10%FBS/DMEM medium was added. Cells were harvested for downstream analysis after 48 hours.

### Fluorescence-based reporter assay

Transfected HCEC-1CT and SW620 cells were trypsinized, centrifuged, and resuspended in 1 mL of phosphate buffered saline (PBS). Triplicates for every transfected vector combination were pulled before fluorescent measurement to increase sensitivity, as subtle changes in promoter activity were important to be detected and to provide a reduction of technical variability that can arise during fluorescent measurement. The green fluorescence intensity of pZsGreen1-DR and pZsGreen1-DR/promoter C, and the red fluorescence intensity of pmCherry-1 and pmCherry-1/promoter D were measured on microplate reader Infinite® 200 PRO (Tecan, Switzerland). The wavelengths used for the measurement were as follows: excitation maximum at 496 nm and emission maximum at 506 nm for pZsGreen1-DR and pZsGreen1-DR/promoter C, and excitation maximum at 587 nm and emission maximum at 610 nm for pmCherry-1 and pmCherry-1/promoter D. Fluorescent values of empty reporter vectors without promoter sequences were subtracted from the values of reporter vector constructs to obtain actual measurement of promoter activity. Measurement of the promoters' C and D activity was calculated the following way:

- promoter C: fluorescence intensity _C/pZsGreen1-DR_ - fluorescence intensity _pZsGreen1-DR_
- promotor D: fluorescence intensity _D/pmCherry-1_ - fluorescence intensity _pmCherry-1_

### Promoter activity estimation using RNA sequencing data

Ensembl annotations (version 109) were used to identify TSSs (defined as the start of the first exon) for all annotated transcripts. TSSs were mapped to SMAD4 genomic sequence to decipher which transcript is regulated by promoters C and D. Promoter activity was estimated as the total transcription initiated at each promoter in FPKM provided by RNA sequencing data.

### *In silico* analysis of *SMAD4* gene promoters

*SMAD4* promoter sequences C and D used in this study were previously experimentally validated 10. Sequences were retrieved as FASTA files using the human GRCh38 assembly at the following positions: chr18: 51029690 - 51030886 for promoter C and chr18: 51046143 - 51047351 for promoter D.

Prediction of the core promoter elements was performed by the tool Integrative Genomics Viewer 24.

The presence of CpG islands in promoter regions was predicted using the online tool MethPrimer with search criteria in GC content ≥50%, island length>200 bp and Observed CpG/Expected CpG ratio ≥ 0.60 25.

Five different bioinformatics tools were used to predict the differential binding of transcriptional regulators to the *SMAD4* gene promoter sequences: Tfsitescan, AliBaba2.1, PROMO, TFBIND and CiiiDER 26-30. Transcriptional regulators binding to either of the promoter regions were collected for all tools, after which differentially bound transcriptional regulators were filtered out. Default values for query parameters and human libraries were used for this purpose. Additional filtering implied differentially bound transcriptional regulators predicted by at least two different tools and protein expression score to be medium or high in colon tissue according to The Human Protein Atlas 31.

### DNA pull-down assay

Approximately 1.2x10^8^ cells of each cell type (HCEC-1CT, SW480 and SW620) were lysed using NT2 buffer with protease inhibitors (NT2 buffer: 50mM Tris-HCl ph7.4, 150mM NaCl, 1mM MgCl2, 0.05%NP-40) and supernatants were stored at -80ºC until further use. Bradford reagent (Thermo Fisher Scientific, USA) was used to determine protein concentration and the same quantity of 2.2 mg was used in DNA pull-down assay for every cell line.

The amplification of the promoter regions was performed using following primers: 5'-CGAGTGTAAACACCTCTGGGGC-3' and 5'- biotin-CCGTCCGAGTTTAACTTGATTC-3' for promoter C, and 5'-GCTGGGTCATATTTACTCTG-3' and 5'- biotin-GCAAGTTTTTAAATCTGCCACC -3' for promoter D. Reaction mixtures and conditions were as described in Reporter vector constructs.

Exactly 2.2 mg of extracted proteins of each cell line and 0.5 μg of each amplified promoter sequence (including sample with dH_2_O instead of promoter sequence as negative control) were incubated for 1h and 30 min with continuous rotation at room temperature. After the incubation, 30 μL of previously washed Dynabeads™ M-280 Streptavidin (Thermo Fisher Scientific, USA) with NT2 buffer was added to the mixture and incubated for an additional 1h and 30 min. Using a magnetic rack, beads-biotinylated promoter sequence-proteins complexes were pulled down from the mixture and, after 3 steps of washing, eluted in NT2 buffer with protease inhibitors and mixed with the same volume of Laemmli buffer (2x Laemmli buffer: 4% SDS, 20% glycerol, 10% 2-mercaptoethanol and 0.125 M Tris HCl, pH approx. 6.8.). Eluted proteins were boiled and stored at -20ºC until further use.

### Proteomics analysis

The eluted proteins were processed by Sodium Dodecyl Sulfate Polyacrylamide Gel Electrophoresis (SDS-PAGE) followed by in-gel trypsin digestion and peptide purification as previously described 32. Peptides were analyzed with a nano Elute HPLC coupled to timsTOF flex mass spectrometer with a Captive Spray ion source (Bruker Daltonics, Bremen, Germany). An analytical 15 cm column was used (C18 column: 150 mm × 75 μm, 1.9 μm diameter beads) with a gradient of 25 min, solvent A (0.1% formic acid) and solvent B (acetonitrile + 0.1% formic acid). Mass spectra were collected using the data-independent acquisition (DIA)-parallel accumulation-serial fragmentation (PASEF) mode.

The raw data files were processed with a DIA-NN library-free search using as a reference the human canonical proteome (https://www.uniprot.org/proteomes/UP000005640, 15/6/2023). All protein identifications required the detection of at least one peptide unique to the protein group for positive protein identification.

## Results

### Analysis of *SMAD4* gene transcripts in RNA sequencing data of colon cell lines cultivated in 3D

**Figure 1:**
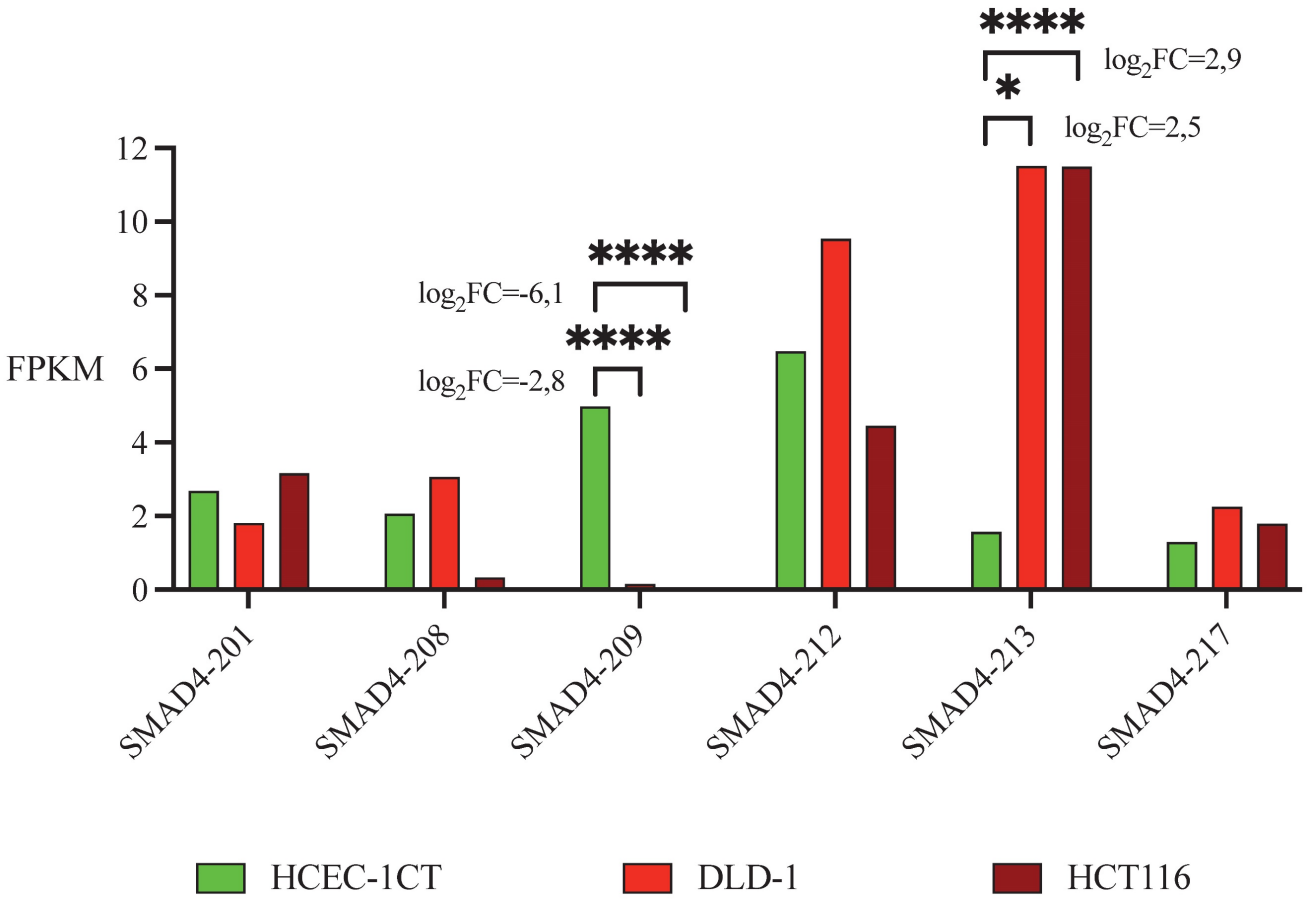
Expression of the SMAD4 transcripts in colon cell lines based on FPKM values obtained through RNA sequencing. Presented are SMAD4 transcripts with moderate/high abundance in non-malignant HCEC-1CT and malignant DLD-1 and HCT116 cell lines. LogFC and p values are presented only for transcripts that have |logFC| values>2.5 being in the same direction (both positive or both negative) in both malignant cell lines in comparison to non-malignant and which p values are <0.05. FPKM - Fragments Per Kilobase of transcript sequence per Millions base pairs. logFC - log 2-fold change, * p<0.05, *** p<0.001.

Sequencing of RNA detected high expression of total *SMAD4* RNA - 31 FPKM in HCEC-1CT and HCT116 cells and 44 FPKM in DLD-1 cells (Table S1). Nine *SMAD4* transcripts have been identified, each detected in at least one of the analyzed cell lines, while other transcripts were considered non-expressed using the FPKM threshold of 0.3 33. Five transcripts had moderate abundance in non-malignant cell line (1<FPKM<10): SMAD4-201, SMAD4-208, SMAD4-209, SMAD4-212 and SMAD4-217. Transcript SMAD4-213 was the only highly expressed transcript (FPKM>10). Differential transcripts expression analysis using edgeR software showed that SMAD4-209 had a higher expression level, and thus was predominant in non-malignant cell line HCEC-1CT in comparison to malignant HCT116 and DLD-1 cell lines, while expression of SMAD4-213 has the opposite direction - it was predominant in malignant cell lines (Table 1 and Figure 1).

### *In silico* analysis of SMAD4-213 transcript

Transcript SMAD4-213 was analyzed by several *in silico* tools to evaluate its potential function. Its TSS was validated at the genomic position GRCh38:chr18:51,047,047 in the promoter D. Classification of SMAD4-213 as a coding transcript by Ensembl was confirmed by Coding Potential Calculator 2 with a very high coding potential score of 0.99. According to the UniProt database, there is experimental evidence at the protein level that a 1036 nt long SMAD4-213 transcript gives rise to 320 amino acids long protein (K7ES96). This protein is located in the nucleus and lacks the MH2 domain characteristic for full-sequence SMAD4 protein. The 3D structure prediction by the UniProt database of the K7ES96 protein showed a preserved tertiary structure of the MH1 domain.

**Figure 2:**
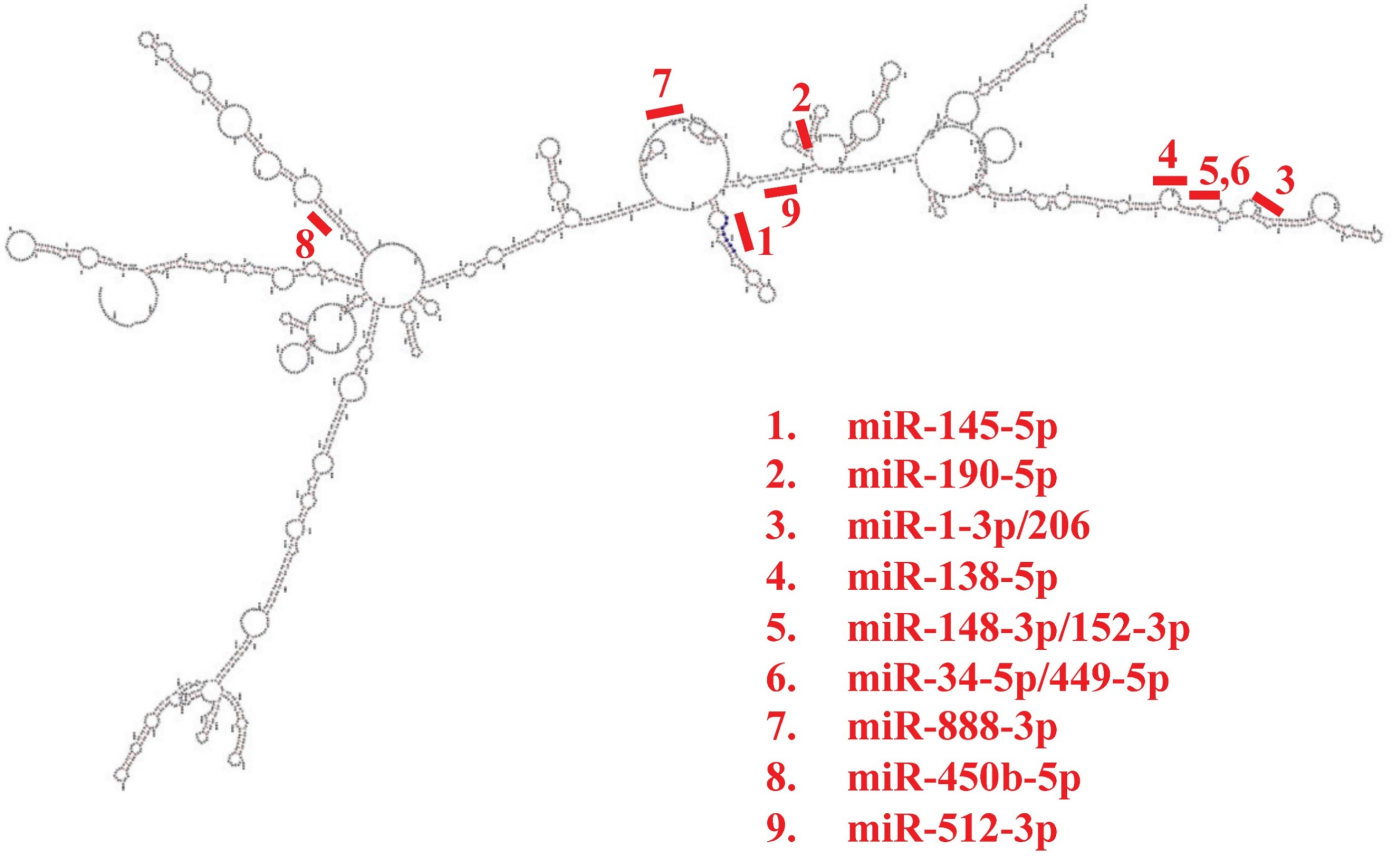
Schematic representation of the predicted microRNA binding sites positions in the secondary structure of the SMAD4-213 transcript obtained by AnnoLnc2 and miRDB tool.

Subcellular localization of SMAD4-213 predicted by AnnoLnc2 and LncLocator tools is indicated to be the cytoplasm. Protein factors that could regulate transcription of SMAD4-213 via AnnoLnc2 predicted binding sites upstream of/overlapping TSS that are expressed in colorectal cancer are CDX2, CEBPB, ELF1, NCOA1, NCOR1, NCOR2 and TFAP4. RNA-binding proteins predicted by RBPsuite to bind to SMAD4-213 transcript are TARDBP, QKI, PABPC4, SRSF1, LIN28B, PCBP1, U2AF2, SRSF9, FUS, CPEB4, IGF2BP2, PUM1, IGF2BP3 and TAF15. Further PPI network analysis for predicted RBPs performed using the STRING database revealed a high interaction score between proteins with PPI enrichment p-value<1.0e-16. The most enriched biological processes were mRNA stabilization, regulation of splicing and regulation of mRNA metabolic processes. The following miRNAs have predicted binding sites in the SMAD4-213 sequence: miR-888-3p, miR-450b-5p and miR-512-3p according to miRDB, and miR-145-5p, miR-190-5p, miR-1-3p/206, miR-138-5p, miR-148-3p/152-3p and miR-34-5p/449-5p according to AnnoLnc2 (Figure 2).

### Activity pattern analysis of *SMAD4* alternative promoters in colon cell lines

**Figure 3:**
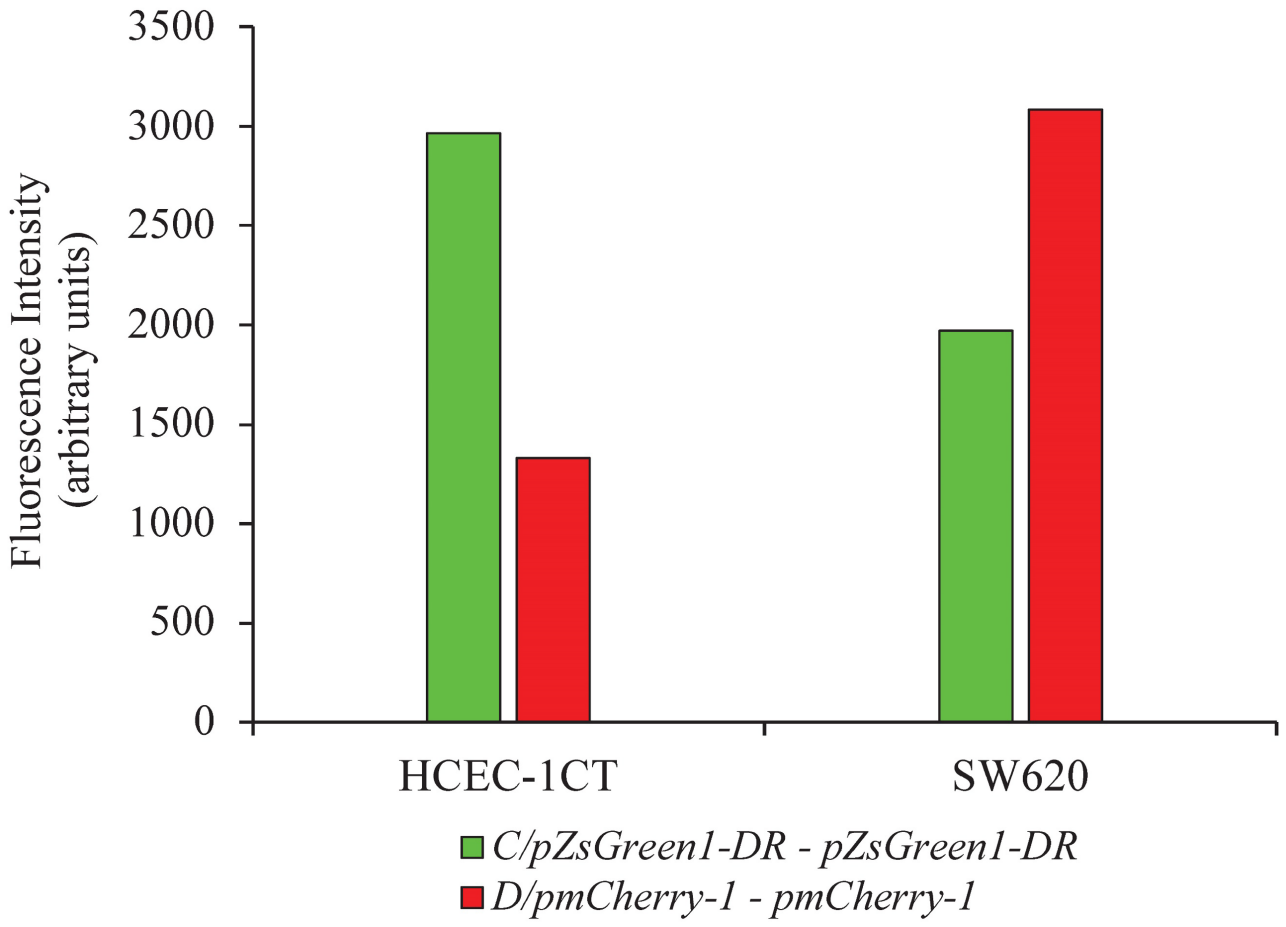
Fluorescence measurement of proteins originating from reporter vector constructs for promoter C and D in malignant SW620 and non-malignant HCEC-1CT cell lines. The fluorescence values of the reporter vectors with cloned promoters from which the values for the empty vectors were subtracted are presented.

Cell lines HCEC-1CT and SW620 were co-transfected with the vector combinations: pZsGreen1-DR and pmCherry-1 or pZsGreen1-DR/promoter C and pmCherry-1/promoter D. Fluorescence intensity measurement of the cells using microplate reader showed difference in the expression of green and red fluorescent proteins representing differential activity pattern of the promoters in SW620 in comparison to HCEC-1CT cells. While promoter C was more active in non-malignant cell, promoter D was more active in malignant cells (Figure 3).

**Figure 4:**
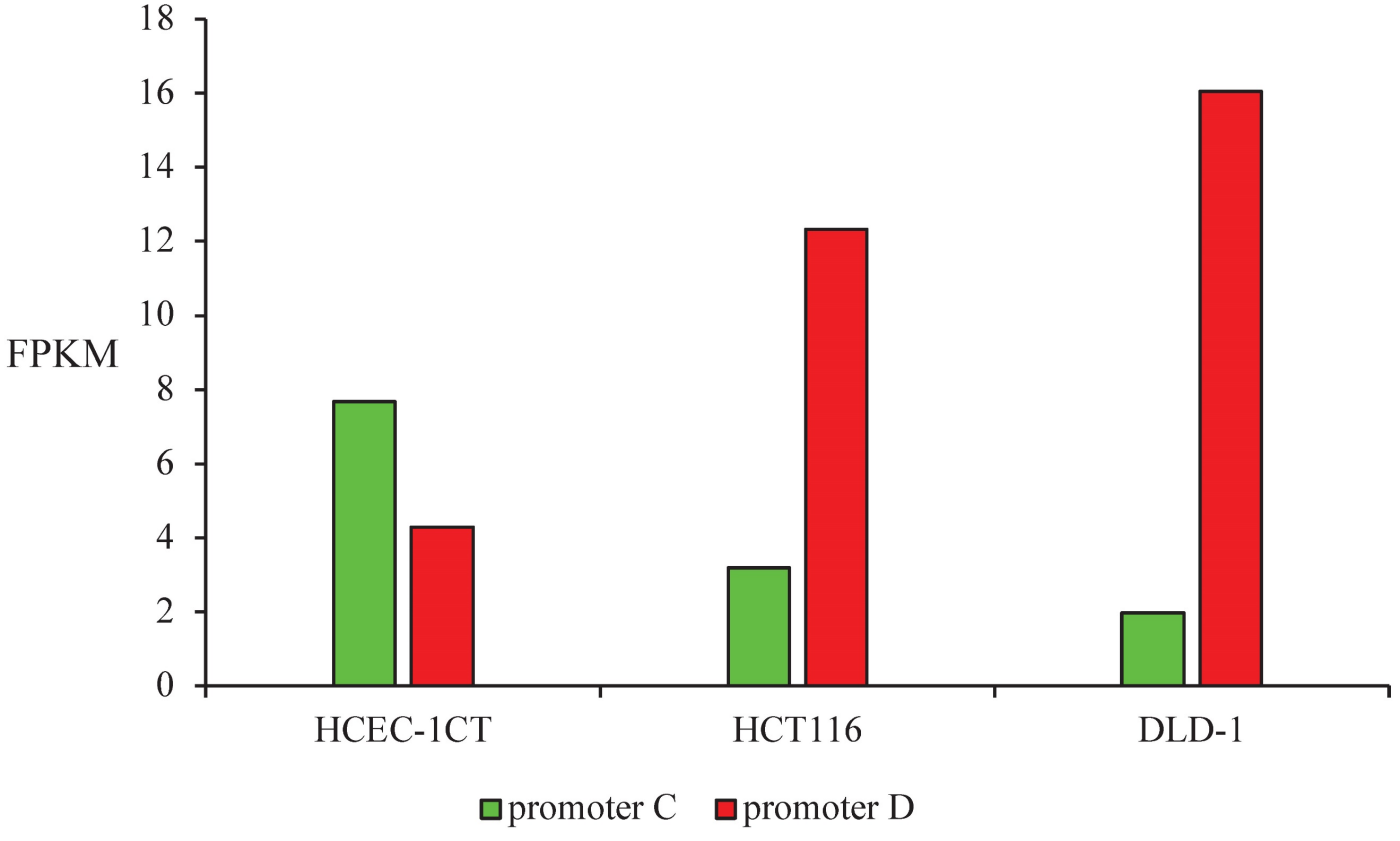
Promoters C and D activity estimated as the total transcription initiated at each promoter in FPKM in non-malignant HCEC-1CT and malignant HCT116 and DLD-1 cell lines.

Promoters C and D activity were estimated as the total transcription initiated at each promoter in FPKM provided by RNA sequencing data. Promoter C had 2 times higher activity than promoter D in non-malignant cells HCEC-1CT, while the activity pattern had the opposite trend in malignant cell lines. Promoter D was 4 times more active in HCT116 and 8 times more active in DLD-1 than promoter C (Figure 4).

### *In silico* analysis of *SMAD4* gene promoters

The core promoter elements predicted by the tool Integrative Genomics Viewer were Initiator (Inr), downstream promoter element (DPE) and downstream TFIIB recognition element (BREd) for promoter C and Inr and BREd for promoter D. CpG island search indicated that the promoter C contains one CpG island throughout whole region (length: 992 bp; position: 101 - 1092), while there were no CpG islands in the promoter D.

**Figure 5:**
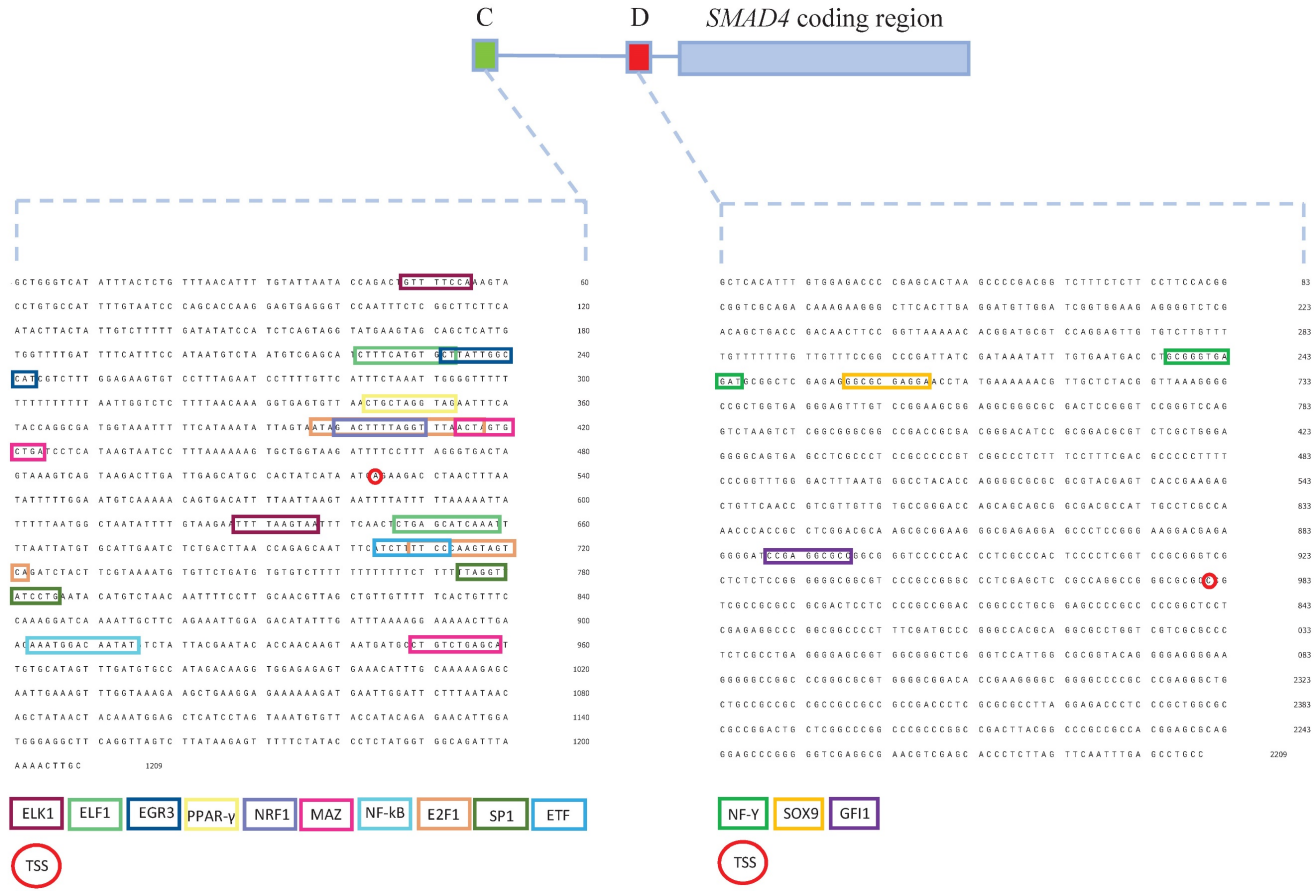
Schematic representation of the SMAD4 gene promoters C and D with binding sites of transcriptional regulators predicted to interact with these sequences. Promoter C is presented as a green box, while promoter D as a red box. Transcriptional regulators predicted to bind to promoter C - NF-kB, EGR3, PPAR-γ, E2F1, MAZ, ELF1, ELK1, ETF1, SP1 and NRF. Transcriptional regulators predicted to bind to promoter D - GFI1, NF-Y and SOX9. TSS - transcription start site.

Prediction of differential binding of transcriptional regulators to the *SMAD4* gene promoters identified 125 proteins potentially binding to promoter C, and 215 proteins potentially binding to promoter D (Table S2). After filtering out differentially bound transcriptional regulators predicted by at least two bioinformatics tools and having protein expression score medium or high in colon tissue, NF-kB, EGR3, PPAR-γ, E2F1, MAZ, ELF1, ELK1, ETF1, SP1 and NRF1were predicted to bind to promoter C while GFI1, NF-Y and SOX9 were predicted to bind to promoter D (Figure 5).

### Proteomics analysis of transcriptional regulators interacting with promoter regions

To determine differentially bound transcriptional regulators to *SMAD4* alternative promoters C and D, DNA pull-down assay coupled with mass spectrometry was applied (Table S3). Negative control (NC) samples were used to eliminate non-specifically bound proteins to magnetic beads from further analysis. Only proteins predicted *in silico* and confirmed by the proteomics analysis were taken into consideration. Transcriptional regulators fulfilling the criteria were SP1, NRF1 and FOXA2. SP1 was detected in SW480 cell line with DNA-probe for promoter C, NRF1 was detected predominantly in the sample with promoter C in HCEC-1CT and in the sample with promoter D in SW620 cell lines, and FOXA2 was detected only in the sample with promoter D in the SW620 cell line.

## Discussion

This study investigated the transcriptional regulation of the *SMAD4* gene in colorectal cancer in search of the differentially regulated transcripts that could serve as biomarkers, their potential roles in malignant cells and the mechanism responsible for their differential expression. SMAD4 has a distinctive role in the initiation and progression of colorectal cancer, but its transcriptional regulation remains understudied 2, 5, 34-36. Alternative promoter usage and its impact on cancer diagnosis and prognosis has been confirmed in colon cancer, prostate cancer, multiple myeloma and hepatocellular carcinoma 37-41. *SMAD4* gene has 24 transcript isoforms reported in the Ensembl database and we aimed to detect their expression profile in malignant and non-malignant cell lines originating from colon tissue. In our previous study, the expression level of the major SMAD4-201 transcript was measured in a set of permanent human colon cell lines and in tumor and corresponding healthy tissue samples from patients with CRC 14. Relative abundance of SMAD4-201 and overall *SMAD4* RNA expression varied in both cell lines and tissue samples and the observed fluctuations in the composition of *SMAD4* transcripts have to be attributed to changes in the expression of the transcripts other than SMAD4-201. High-throughput next-generation RNA sequencing was employed in this study to establish the expression profile of *SMAD4* transcripts in non-malignant cell line HCEC-1CT and malignant cell lines HCT116 and DLD-1 cultured in 3D. In the previous study, where relative abundance was measured using real-time qPCR and the same three cell lines were cultured in 2D, the relative expression level of SMAD4-201 was 39-47% of the *SMAD4* transcript pull 14. Using next-generation RNA sequencing, the detected expression level of SMAD4-201 was 4-10% of the total *SMAD4* expression level. Those distinctions might be due to the difference in applied methodological approaches and cell culturing. Both approaches applied in this study are more advanced and accurate, as next-generation sequencing provides better sensitivity, while culturing cells in 3D as spheroids ensures that the obtained transcriptome better reflects the state of the cells from their natural environment.

Transcript SMAD4-209 was detected almost exclusively in non-malignant cells and could be considered a hallmark of healthy tissue. Its TSS is located in the promoter C, adjacent to the first non-coding exon. SMAD4-209 is coding for full-length SMAD4 protein and contributes to the SMAD4 protein pull with a tumor-suppressive role and maintenance of tissue homeostasis and cell cycle regulation. Contrary, transcript SMAD4-213 was predominantly expressed in malignant cells. Its contribution to the overall *SMAD4* expression level is 5% in HCEC-1CT, 26% in HCT116 and 37% in DLD-1 cells, making it the most abundant transcript in malignant cells. Its TSS is positioned in the promoter D region, adjacent to the first coding exon. According to the clinical tissue samples analysis provided by the GEPIA2 tool, SMAD4-213 transcript has higher expression in colon and rectal adenocarcinoma (2.28 Transcripts Per Million - TPM and 2.22 TPM) in comparison to healthy colon and rectum tissue (0.62 TPM and 0.49 TPM), which is in line with our analysis. Overexpression of SMAD4-209 in malignant cell lines would be of benefit for exploring its possible anti-tumor effect, while overexpression of SMAD4-213 in non-malignant cell lines would confirm its tumor-promoting role and both research directions would elucidate possible mechanism underlying those effects.

Among transcripts with moderate abundance, SMAD4-212 had noticeable expression in all three analyzed cell lines, but showed no distinction between malignant and non-malignant cells. Transcripts SMAD4-208 and SMAD4-212 showed variation in expression level in two different CRC cell lines DLD-1 and HCT116. This is probably due to the different consensus molecular subtype (CMS) of the cell lines, since DLD-1 belongs to CMS1 and HCT116 belongs to CMS4 42. In addition, cell lines belong to different Dukes types: DLD-1 - Dukes type C and HCT116 - Dukes type A 43, 44. Different genomic, transcriptomic and epigenomic landscapes of the cell lines DLD-1 and HCT116 certainly contribute to the divergent transcript levels. Since both transcripts do not code for the full-length protein and, hence, do not contribute to the pull of functional SMAD4 in the cell, their role might be of regulatory nature and fine quantity differences in the cell might be of importance for their role.

Considering the differential expression of the SMAD4-209 and SMAD4-213 transcripts in non-malignant and malignant cells, their expression level ratio might be a sensitive indicator of the malignant status of the tissue and could serve as a biomarker for CRC detection. Since many patients with CRC have aberrant TGF-β/SMAD4 signaling which may affect transcriptional regulation, localization and stability of those transcripts, their biomarker potential should be further investigated in the human tissue samples of CRC patients by combined analysis of SMAD4-209 and SMAD4-213 expression levels.

In order to decipher the possible roles of SMAD4-213 in CRC, we performed *in silico* analysis, which indicated that it is a coding transcript located in the cytoplasm. According to the UniProt database, it encodes experimentally validated 320 amino acids long protein (UniProt ID: K7ES96) which is quite truncated in comparison to full-length SMAD4 protein. The primary structure of the SMAD4 protein consists of the MH1 domain responsible for DNA binding, linker region and the MH2 domain responsible for transcriptional activity by interacting directly with the MH1 domain of other SMAD proteins 34. Truncated protein K7ES96 lacks an MH2 domain, while 3D structure prediction showed a preserved tertiary structure of the MH1 domain. Subcellular localization of the K7ES96 is the nucleus, similar to the full-length protein, which can be explained by the preserved nuclear localization signal (NLS) within the MH1 domain. Taking everything into account, the proposed functional cascade would include transcription of the SMAD4-213 from promoter D, its translocation in the cytoplasm where it is translated into K7ES96, which is then translocated to the nucleus where it can bind DNA with MH1 domain but can not interact with partner proteins due to the lack of the MH2 domain. This truncated protein can not regulate transcription of the target genes through interaction with partner proteins, like full-length SMAD4 protein, and it may contribute to steric obstruction at the target genes binding sites by preventing binding of other transcriptional regulators, disturbing this way TGF-β signaling and potentially promoting cell processes characteristic for malignant transformation. In support of the coding role of the SMAD4-213 transcript, our analysis of RBPs predicted to bind to the transcript and their functional PPI network analysis revealed enriched interactions that are part of biological processes responsible for the stabilization and splicing of SMAD4-213 in the nucleus. Among predicted transcriptional regulators that could influence the expression of this transcript in CRC, TFAP4 promotes metastases and EMT and predicts poor prognosis in CRC 45-47. It is also predicted by both our *in silico* analysis to control transcriptional regulation of SMAD4-213 and to have a binding site in the promoter D region from which SMAD4-213 is transcribed. Although our *in silico* analysis gave insight into the possible role of the SMAD4-213 transcript and protein K7ES96 encoded by this transcript, further experimental validation of the results will contribute more thoroughly to its role in malignant transformation.

None of the predicted miRNAs have a binding site in the 3'UTR or 5'UTR regions of the SMAD4-213, but in the coding region, hence it is unclear whether they exert enhancing or repressing roles in the regulation of SMAD4-213 expression 48. The dual role of miRNAs in general is a consequence of the complex interplay between different cofactors or RNA-binding proteins that interact with miRNAs and their target mRNAs. In addition, some miRNAs may exert a dual role depending on the cellular context underscoring their versatility and complexity as regulators of gene expression 49. Several miRNAs that are predicted to interact with SMAD4-213 have a tumor-suppressive role in CRC and the sponging function of SMAD4-213 may disable that role. miR-145-5p, miR-206 and miR-138-5p have been found to suppress proliferation, metastasis and EMT in CRC by its involvement in several pathways 50-57. Besides coding for the truncated protein, the structure and localization of SMAD4-213 point to its potential additional role. Dual roles of RNA molecules defined by their coding and non-coding potentials is a growing concept 58. mRNAs can exert their regulatory functions mediated by the interaction of their 3'UTR non-coding region with regulatory factors, RNA-binding proteins and miRNAs acting that way as a competing endogenous RNA (ceRNA). Further validation studies of predicted miRNAs should elucidate the underlying mechanism and explore this complex RNA regulatory network.

Considering the differential expression of SMAD4-209 and SMAD4-213 transcripts in non-malignant vs. malignant tissue, we aimed at functional analysis of their respective promoters to investigate the possible mechanism that could influence their activity pattern in CRC. The activity pattern of *SMAD4* promoter regions C and D was measured by a fluorescence-based reporter assay. The promoters' C and D activity was assessed by measuring the fluorescence intensity of green fluorescent protein originating from reporter vector construct pZsGreen1-DR/promoter C and red fluorescent protein originating from reporter vector construct pmCherry-1/promoter D, respectively. The experiment entailed pooling of transfected cells into a single sample for fluorescence measurement to avoid the variabilities due to the transfection process and allow maximization of the signal-to-noise ratio while ensuring that the measurements were sufficiently powered to detect subtle changes in promoter activity. Fluorescence measurement on the microplate reader showed higher promoter C activity in non-malignant HCEC-1CT in comparison to the malignant SW620 cell line, while promoter D activity showed the opposite trend when being more active in malignant in comparison to non-malignant cells. Calva et al. found a similar activity pattern of promoter C, where higher activity was detected in normal colon cell line CCD-18Co than in CRC cell line HCT116. However, while we detected higher promoter D activity in malignant cell line SW620, they did not detect any promoter D activity in HCT116 cells 10. This might be due to the difference in cancer stages, since SW620 is a more advanced stage than HCT116, indicating that promoter D might not yet overtake the transcriptional activity 59. This result points to the differential activity of *SMAD4* promoters and confirms the hypothesis on their aberrant use in cancer.

Promoters' C and D activity pattern was also estimated from our transcriptomics data by defining the promoter activity as the total transcription initiated at each promoter. The same activity pattern was obtained as in the reporter assay. Promoter C was more active in non-malignant cell line HCEC-1CT, while promoter D was more active in both malignant cell lines. Promoter D activity was higher in DLD-1 compared to the HCT116 cells, which points to progressed dysregulation in the more advanced tumor stage. The translational potential of the activity pattern of the *SMAD4* promoter regions can be further examined as a biomarker tool or therapeutic target.

To further elucidate possible mechanisms that could influence differential *SMAD4* promoters' activity in CRC, we performed *in silico* analysis of promoters C and D and the pull-down assay coupled with mass spectrometry to validate the results of *in silico* analysis. We found that both promoter regions contain core promoter elements of canonical promoters 60. Promoter C contains a CpG island throughout the whole sequence, while there was no CpG island found in promoter D. Although promoter C contains a CpG island, analysis of this region in CRC patients and cell lines showed no hypermethylation of the promoter region and excluded influence of this epigenetic modification on transcriptional regulation of *SMAD4* 12, 61. The presence of binding motifs of transcriptional regulators indicates potential differences in activity that are in line with the observed promoter activity patterns. Transcriptional regulators that are predicted to bind exclusively to promoter C are involved in cell cycle regulation, proliferation, cell development and apoptosis. However, transcriptional regulators predicted to bind to promoter D regulate cell cycle and development in a homeostatic cell state, but have been found to promote malignant transformation in CRC. Among those transcriptional regulators that interact with promoter D, NF-Y and SOX9 predominantly bind to cancer-associated promoters 62, 63. Transcriptional regulators predicted in a previous study to have a binding site in the promoter C were mostly involved in a cell-cycle regulation acting in a way that negatively regulates cell division, which is in line with our findings. Among them, transcriptional regulators that are also predicted in our analysis are ELK1 and ELF1 - members of the ETS gene family, SP1 and NRF1. However, they predicted a number of conserved transcriptional regulators for promoter D that have a binding site within the first coding exon of *SMAD4*, while our prediction analysis pointed to only three transcriptional regulators that have a binding site upstream of the translation start site (Fig. 4) 10. This discrepancy could be explained by the fact that we used *in silico* tools that predict only conserved transcriptional regulator binding sites and we aimed our analysis on differentially active transcriptional regulators within C or D promoters.

Proteomics analysis identified several candidate transcriptional regulators that could contribute to the differential activity of promoters C and D in malignant in comparison to non-malignant tissue. Binding sites for the transcriptional regulators SP1, NRF1 and FOXA2, both predicted in the previous study and also in our *in silico* analysis, were confirmed in our proteomics data 10. We found SP1 to bind to promoter C in the SW480 cell line. SP1 is a transcriptional regulator that plays a significant role in CRC development and progression and is highly expressed in human CRC tissues compared to adjacent normal colon tissues 64. Results from the previous study regarding the expression pattern of SP1 is in line with our findings since we did not detect SP1 in the non-malignant HCEC-1CT cell line. NRF1 was shown to predominantly bind to promoter C in the HCEC-1CT cell line and to promoter D in the SW620 cell line. NRF1 has a distinctive role in homeostasis of proteasome to ensure protein quality control during development and in adult tissues. However, it is specifically activated in cancer cells and has an oncogenic role in the tumor environment, which might be the reason why we detected its binding to promoter D in the malignant SW620 cell line 65. The dual role of the NRF1 in healthy and tumor tissue is in line with our proteomics findings and could contribute to the explanation of the differential activity pattern of the promoters. FOXA2 was shown to bind to promoter D in the SW620 cell line. A recent study found exclusive binding of FOXA2 to genome-wide promoters in SW620 in comparison to the SW480 cell line and its importance in CRC metastasis 66. Our proteomics data confirmed the binding of HOXA2 only to promoter D in the SW620 cell line, while there was no binding in SW480 or HCEC-1CT cell lines, pointing to the role of promoter D in CRC progression and metastasis. Our proteomics findings showed abundant non-specific binding of proteins to magnetic beads in NC samples, although multiple washing steps with high-salt concentration buffer were performed. A possible solution to this issue would be the optimization of binding and washing with multiple buffers or adding non-competitive and competitive oligonucleotide probes, capable to discriminate non-specific proteins-DNA interactions. Another approach that could improve more targeted binding of the transcriptional regulators could be employing EMSA (electrophoresis mobility shift essay) coupled with mass spectrometry 67. To further validate the binding of transcription factors identified by mass spectrometry to its target sequence in the promoter, surface plasmon resonance could be exploited.

Our study provides preliminary data based on state-of-the-art omics approaches and bioinformatic tools that contribute to understanding the role of alternative transcription regulation in cancer. However, these findings have not been validated on clinical samples, which limits their direct applicability to real-world clinical settings. Additionally, our study provides a glimpse into a molecular mechanism involved in transcriptional dysregulation, while lacking experimental functional studies to confirm the definitive impact of the proposed transcripts in the malignant transformation. These limitations highlight the need for future research to further validate our findings and understand their full potential.

## Conclusions

This study found differential expression levels of SMAD4-209 and SMAD4-213 transcripts in non-malignant and malignant cells. *In silic*o analysis predicted distinctive roles of these transcripts in the cell that could explain their opposite levels in CRC: SMAD4-209 is coding for full-length SMAD4 protein - a major regulator of homeostasis and cell cycle in healthy tissue, while SMAD4-213 is coding for a truncated protein that could affect highly regulated TGF-β signaling and push the cell into homeostatic dysregulation, and has a predicted secondary structure with potential for sponging miRNAs with tumor suppressive roles in CRC. Since SMAD4-209 originates from promoter C and SMAD4-213 from promoter D, we investigated the activity pattern of *SMAD4* promoters and found their differential activity in CRC: promoter C had higher activity in non-malignant cells while promoter D had higher activity in malignant cells, which was confirmed by our transcriptomics data. *In silico* analysis predicted transcriptional regulators with differential binding affinity to promoters C and D which could explain possible mechanisms for their opposite activity in CRC. Furthermore, proteomics analysis validated several transcriptional regulators predicted by *in silico* analysis with a known role in CRC.

Based on the observed dysregulation of SMAD4-209 and SMAD4-213 in malignant vs. non-malignant colon cells, we propose that their expression ratio might be a solid biomarker candidate for colorectal cancer detection. A differential pattern of the respective promoters' activity was observed that corresponds to the expression of transcripts, confirming the role of alternative promoters in context-specific isoform expression. The investigated *SMAD4* promoters and transcripts harbor translational potential that should be further investigated and exploited for CRC diagnostics and treatment.

## Supplementary Material

Supplementary tables.

## Figures and Tables

**Table 1:** LogFC and p values calculated using edgeR software for differential expression between non-malignant HCEC-1CT and malignant DLD-1 and HCT116 cell lines in moderately/highly expressed SMAD4 transcripts. Transcripts with |logFC| values>2.5 being in the same direction (both positive or both negative) for non-malignant vs. malignant cell lines and having p value <0.05 are bolded. LogFC - log 2 fold change

	HCEC-1CT vs. DLD-1 (transcripts.readcount)		HCEC-1CT vs. HCT116 (transcripts.readcount)	
	logFC	p value	logFC	p value
SMAD4-201	0.20569927	0.31695615	0.22282412	0.29155168
SMAD4-208	0.85781196	0.01275749	-2.5653814	2.4127E-08
**SMAD4-209**	**-2.7590642**	**2.181E-06**	**-6.1302512**	**3.1798E-29**
SMAD4-212	1.33479567	2.8452E-09	-0.5502299	0.00932189
**SMAD4-213**	**2.51571909**	**0.0492606**	**2.85442619**	**3.1517E-28**
SMAD4-217	1.23920715	4.2997E-06	0.45849105	0.08718781

## Abbreviations

SMAD4: SMAD family member 4
TGF-β: transforming growth factor-beta
CRC: colorectal cancer
TSS: transcription start site
RAMPAGE: RNA Annotation and Mapping of Promoters for the Analysis of Gene Expression
ENCODE: Encyclopedia of DNA Elements
DMEM: Dulbecco′s Modified Eagle′s - Medium
FBS: fetal bovine serum
FPKM: Fragments Per Kilobase of transcript sequence per Millions base pairs
RBPs: RNA-binding proteins
PPIs: protein-protein interactions
PBS: phosphate buffered saline
Inr: Initiator
DPE: downstream promoter element
BREd: downstream TFIIB recognition element
ceRNA: competing endogenous RNA
EMSA: Electrophoretic Mobility Shift Assay
SDS-PAGE: Sodium Dodecyl Sulfate Polyacrylamide Gel Electrophoresis
