# Supplementary material for: Dysregulation of transcripts SMAD4-209 and SMAD4-213 and their respective promoters in colon cancer cell lines
Source: J Cancer. 2024 Aug 6;15(15):5118–31. doi: 10.7150/jca.98911 (PMC11310865; doi:10.7150/jca.98911)
Supplement: Supplementary file 1 — Supplementary tables. [file jcav15p5118s1.pdf]

Table S1. High-throughput next-generation RNA sequencing of non-malignant HCEC-1CT and malignant HCT116 and DLD-1 cell lines originating from colon tissue. In the blue-colored table are presented FPKM values for the whole expression of the *SMAD4* gene, while in the green-colored table are presented FPKM values for each SMAD4 transcript.

gene_id	gene_name	gene_locus
ENSG00000141646	SMAD4	18:51028394-51085045

transcript_id	transcript_name	gene_id	gene_name	transcript_locus
ENST00000589076	SMAD4-207	ENSG00000141646	SMAD4	18:51030100-51048708
ENST00000590061	SMAD4-210	ENSG00000141646	SMAD4	18:51030213-51048857
ENST00000592911	SMAD4-215	ENSG00000141646	SMAD4	18:51030597-51058154
ENST00000588745	SMAD4-205	ENSG00000141646	SMAD4	18:51047047-51078467
ENST00000592186	SMAD4-214	ENSG00000141646	SMAD4	18:51047047-51078467
ENST00000342988	SMAD4-201	ENSG00000141646	SMAD4	18:51030213-51085039
ENST00000398417	SMAD4-202	ENSG00000141646	SMAD4	18:51029614-51085045
ENST00000591126	SMAD4-212	ENSG00000141646	SMAD4	18:51052326-51079777
ENST00000611848	SMAD4-217	ENSG00000141646	SMAD4	18:51058353-51079143
ENST00000591914	SMAD4-213	ENSG00000141646	SMAD4	18:51030213-51049324
ENST00000589941	SMAD4-209	ENSG00000141646	SMAD4	18:51044959-51048798
ENST00000589706	SMAD4-208	ENSG00000141646	SMAD4	18:51047179-51049565
ENST00000588860	SMAD4-206	ENSG00000141646	SMAD4	18:51028394-51048816
ENST00000590499	SMAD4-211	ENSG00000141646	SMAD4	18:51066822-51077291
ENST00000586253	SMAD4-204	ENSG00000141646	SMAD4	18:51077251-51078870
ENST00000593223	SMAD4-216	ENSG00000141646	SMAD4	18:51067113-51077130
ENST00000585448	SMAD4-203	ENSG00000141646	SMAD4	18:51051093-51054916

HCT116 (FPKM)	DLD-1 (FPKM)	HCEC-1CT (FPKM)
30.884188	43.799004	30.83934

HCT116 (FPKM)	DLD-1 (FPKM)	HCEC-1CT (FPKM)
0	0	0
0	0	0
0	0	0
0	0	0
0	0	0
3.168145	1.815537	2.689202
0.013276	0.001923	0.038767
4.459052	9.533424	6.472704
1.792871	2.256931	1.294333
11.492303	11.510384	1.571642
0.036732	0.158341	4.97713
0.330983	3.068607	2.06937
0	0	0
0.288089	1.159342	0.478574
0.275016	0.487777	0.344155
0.515416	1.476431	0.664413
0	0	0

Table S2. Diferentially bound transcriptional regulators to SMAD4 promoters C and D predicted by bioinformatic tools Tfsitescan, AliBaba2.1, PROMO, TFBIND and CiiiDER.

Promoter C	Promoter D
ADF1	OCT1
AHR	AFP1
AP2	ALX1
AP3	ALX4
ARNT::HIF1A	ANTP
CMYC	AP1
CPE_BINDING_PRO	AP1FJ
CTCFL	ARID3B
E1AF	ARID5A
E2F1	ARX
E2F2	ASCL1
E2F4	ATF3
E2FDRTF	ATOH1
E4F1	ATOH7
EBF1	BATF
EBF3	BATF::JUN
EGR1	BATF3
EGR3	BHLHA15
EGR4	BRACH
EHF	BRN2
ELF1	BSX
ELK1	C/EBP
ELK3	C/EBPBETA
ELK4	C/EBPDELTA
ETF	CDPCR1
ETS1	CDPCR3HD
ETS2	CDX4
ETV4	CEBPALPHA
FEV	CF1
FOXD1	CIZ8
FOXE1	CJUN
FOXK1	COUP
FOXK2	CREB
FOXO6	CUX1
FOXP2	CUX2
FOXQ1	DBP
GABPA	DDIT3::CEBPA
GCF	DL
GCM1	DLX1
GLI3	DLX3

HAND1::TCF3	DLX4
HEN1	DMRTA2
HES1	DPRX
IRF2	DRGX
ISL1	E4BP4
ISL2	ELF5
KLF1	EN1
KLF4	EOMES
KLF5	FOSB::JUNB
KLF9	FOSL1::JUNB
KROX20	FOSL1::JUND
LVC	FOXA2
MAZ	FOXA3
MEF2C	FOXB1
MEP1	FOXC1
MSANTD3	FOXC2
MTBF	FOXH1
NET_SAP2	FOXI1
NF1B1	FOXJ2
NFIC	FOXJ3
NFKAPPAB1	FOXN3
NFMUE1	FOXO1
NKX23	FOXP1
NKX25	FTZ
NKX3.2	GATA1
NKX31	GATA2
NKX32	GATA3
NR1I3	GBX1
NR4A2	GBX2
NRF1	GCN4
OCT1A	GFI1
OSR1	GFI1B
OSR2	GLO
OVOL1	GMEB1
OVOL2	GMEB2
P40X	GRE
PAX4PD	GSX1
PAX5	H3ABP
PEA2_2	HESX1
PLAGL1	HFH2
POU3F2	HIC1
POU3F3	HLF
POU5F1B	HLTF
PPARALPHA:RXRAL	HMBOX1
R2	HMX2
RAP1	HNF1A

RARALPHA1	HNF1B
RARBETA:RXRALPH	HNF1C
REB1	HNF3B
RELA	HNF4A
RELB	HNF4G
REPRESSOR_OFA	HOXA10
RORB	HOXA11
RREB1	HOXA2
RUNX3	HOXA6
SIF	HOXA7
SOX12	HOXB5
SP1	HOXB8
SP3	HOXB9
SP8	HOXC10
SPDEF	HOXC11
STAT5A	HOXC12
TCF12	HOXC13
TCF3	HOXC9
TCF7L1	HOXD10
TCFL5	HOXD11
TFAP2A	HOXD12
TFAP2B	HOXD3
TFAP2C	HOXD9
VEZF1	HSEBINDING_PRO
WT1	ICSBP
YY2	IK3
ZBTB14	ISGF3
ZBTB33	ISX
ZBTB6	JUN::JUNB
ZBTB7A	LBX2
ZBTB7C	LHX1
ZFP57	LHX3
ZFX	LHX6
ZIC1	LHX9
ZIC3	LIN54
ZIC4	LMX1A
ZNF219	LMX1B
ZNF423	LVB
ZNF460	LYF1
	MAFB
	MAX
	MEF2A
	MEIS2
	MEOX2
	MGA
	MIXA

MIXL1
MNX1
MSGN1
MSX2
MSX3
MUEBPC2
MYBL1
NFATC1
NFATC4
NFBA1
NFE2L2
NFIX
NKX25
NKX28
NKX3_1R52C
NKX61
NKX63
NOBOX
NOTO
NR2C2
NR2E1
NR4A1
NR5A1
NRL
NRSF
OCT2.1
OTX1
PAX6
PDX1
PIT1A
PITX1
PITX2
PITX3
POU2F2
POU6F1
POU6F2
RAX2
RBPJKAPPA
RHOX11
RORA2
RUSH
SCRT1
SCRT2
SEF1
SHOX2
SIX1

SMAD2::SMAD3
SNAI1
SNAI2
SOX13
SOX14
SOX15
SOX17
SOX2
SOX3
SOX8
SOX9
STAT1::STAT2
STAT2
TAL1ALPHAE47
TAL1BETAE47
TAL1BETAITF2
TBP
TBR1
TBX1
TBX2
TBX3
TBX4
TCF7L2
TEC1
TFAP4
TWIST2
UNCX
VBP
VMAF
VMW65
XBP1
XFD1
ZBTB12
ZEN1
ZKSCAN1
ZNF217
ZNF354C

Table S3. Proteomics analysis of transcriptional regulators interacting with promoter regions												
**Protein.Group**	**Protein.Ids**	**Protein.Names**	**Genes**	**HCEC1CT_(**	**HCEC1CT_**	**HCEC1CT_N**	**SW480_C**	**SW480_D**	**SW480_NC**	**SW620_C**	**SW620_D**	**SW620_NC**
A0A024R1R8;Q9Y2S6	Q9Y2S6;A0A024R1R8	TMA7B_HUMAN;TMA7_HUMAN	TMA7;TMA7B				3912.09	548.562			3760.8	
A0A075B6H7;A0A0C4DH55;P0162	A0A075B6H7;A0A0C4DH90;A0A0C4DH55;P01624	KV315_HUMAN;KV37_HUMAN;KVD07_HUM	IGKV3-15;IGKV3-7;IGKV3D-7									
A0A0B4J2D5;P0DPI2	P0DPI2;A0A0B4J2D5	GAL3A_HUMAN;GAL3B_HUMAN	GATD3;GATD3B						3307.97			
A0A0C4DH36	A0A0C4DH36	HV338_HUMAN	IGHV3-38	3366.45			2239.52	1139.11	1193.9			
A0AV96	A0AV96	RBM47_HUMAN	RBM47					4957.72		878.12	10243.4	5718.77
A0AVT1	A0AVT1	UBA6_HUMAN	UBA6					3300.5	3384.65	3090.23		
A0FGR8	A0FGR8	ESYT2_HUMAN	ESYT2	3744.35	3037.41	5838.97	3463.32	6437.14	4607.32	16105.9	5262.65	11386.9
A0MZ66	A0MZ66	SHOT1_HUMAN	SHTN1						872.127	1117.13	612.393	1082.73
A1L0T0	A1L0T0	HACL2_HUMAN	ILVBL		3183.67		1839.74	3269.61	4218.14	4285.24	3259.41	3600.14
A1L390	A1L390	PKHG3_HUMAN	PLEKHG3					3813.71	2069.52	11804.2		8161.37
A2RRP1	A2RRP1	NBAS_HUMAN	NBAS		997.991		972.653	1788.9	936.137	1439.25	1061.94	716.029
A3KMH1	A3KMH1	VWA8_HUMAN	VWA8	3401.23	2447.23	3620.95	3835.52	4495.24	3793.46	4114.52	3906.2	3788.36
A5YKK6	A5YKK6	CNOT1_HUMAN	CNOT1	5826.81	4832.17	6319.4	7985.91	5579.34	5700.35	7827.39	8087.22	7570.27
A6NHL2	A6NHL2	TBAL3_HUMAN	TUBAL3	4017.02	2351.02	2353.52	3331.23	1649.45		4070.88	3179.3	4912.77
A6NHR9	A6NHR9	SMHD1_HUMAN	SMCHD1	1915.97	1335.46	1425.82	1638.7	2349.65	2384.61	4606.64	1153.86	4287.43
A6NJ78	A6NJ78	MET15_HUMAN	METTL15			1832.28	2524.56	2547.06	1772.65	2271.28	2134.43	2800.83
A7E2V4	A7E2V4	ZSWM8_HUMAN	ZSWIM8		4178.97		1783.41	2644.49	1742.23	1341.63	1847.73	2354.47
A7KAX9	A7KAX9	RHG32_HUMAN	ARHGAP32							2904.66		3242.06
A8K2U0	A8K2U0	A2ML1_HUMAN	A2ML1			5000.6	3075.81		2161.16	1663.22	2468.79	2472.46
A8MWD9;P62308	A8MWD9;P62308	RUXGL_HUMAN;RUXG_HUMAN	SNRPG;SNRPGP15				4631.27	3522.3	1748.43	2905.09	8285.89	3090.33
B5ME19;Q99613	Q99613;B5ME19	EIF3C_HUMAN;EIFCL_HUMAN	EIF3C;EIF3CL	11273.8	9152.91	10113.7	31143.6	18356	23284.6	22413.8	13852.8	17343.3
B9A064;P0CG04	P0DOY3;P0CF74;P0DOY2;B9A064;P0CG04;A0M8Q6	IGLC1_HUMAN;IGLL5_HUMAN	IGLC1;IGLL5	7755.68	4014.98	16783.3	4606.89	2862.19	1984.3	3318.67	5421.99	2935.48
C9JN71	C9JN71	ZN878_HUMAN	ZNF878	15906.3	12614.1	27135.8	6140.17	82885.2	41629.3	46807.2	15646.3	51768.2
C9JRZ8	C9JRZ8	AK1BF_HUMAN	AKR1B15									
E9PAV3	E9PAV3	NACAM_HUMAN	NACA	5816.59		3414.06	6113.21	6427.77	12197.5	5970.33	8330.1	4662.23
E9PRG8	E9PRG8	CK098_HUMAN	C11orf98				1067.53	5282.31	2824.05	1157.12	1838.14	1328.83
L0R819	L0R819	ASURF_HUMAN	ASDURF						1328.38	2358.44		2247.89
O00116	O00116	ADAS_HUMAN	AGPS				6667.37	7746.93	4649.5	4848.19	4633.53	4426.85
O00139	O00139	KIF2A_HUMAN	KIF2A	3113.38	4465.88	3215.25	2904.16	4814	4358.48	2975.27	1394.49	3929.22
O00148	Q13838;O00148	DX39A_HUMAN	DDX39A	37065	17437.6	33474.6	54332	34888.2	34843	51756.8	63405	45559.4
O00151	O00151	PDLI1_HUMAN	PDLIM1	17078.5	3470.77	15021.5	6955.95	8242.18	17046.8	1860.72	61434.9	2858.57
O00154	O00154	BACH_HUMAN	ACOT7	4145.93	3641.01	5154.91	4512.3	4298.95	6288.06	4884.39	2514.21	3335.21
O00159	O00159	MYO1C_HUMAN	MYO1C	42363.8	41480.4	41038	24522	33821.6	23526.5	187277	69217.3	138733
O00161	O00161	SNP23_HUMAN	SNAP23	3702.23	5299.14	4230.72	21155.5	52926.5	58516.1	86889.4	18689.6	80737.5
O00165	O00165	HAX1_HUMAN	HAX1				3982.59	769.707	1807.84	2274.68	5965	8728.16
O00170	O00170	AIP_HUMAN	AIP	4399.82	2006.63	4240.11	7473.25	3036.2	2249.69	6139.69	2880.92	5784.45
O00178	O00178	GTPB1_HUMAN	GTPBP1		1187.09	1455.22	1772.99	2287.98	2179.89	1103.06		1339.52
O00182	O00182	LEG9_HUMAN	LGALS9							2408.85		2347.49
O00186	O00186	STXB3_HUMAN	STXBP3	1208.11	1107.4		1582.13	3438.82	2388.95	1399.19	898.802	1737.2
O00203	Q13367;O00203	AP3B1_HUMAN	AP3B1	5435.87	6382.26	4133.51	7246.42	5756.16	4298.9	3780.78	5289.88	2554.56
O00213	O00213	APBB1_HUMAN	APBB1					2233.29	1968.74			
O00214	O00214	LEG8_HUMAN	LGALS8				5525.12	6848.34	6542.16	26201.8	8511.46	24288.6
O00231	O00231	PSD11_HUMAN	PSMD11	15388.9	16196.5	16469	17749.4	19223.9	20832.3	21067.8	12093.4	15987.5
O00232	O00232	PSD12_HUMAN	PSMD12	11015.5	9855.71	12409.8	18232.5	17595.3	18903.9	14007.7	8804.9	15261.1
O00233	O00233	PSMD9_HUMAN	PSMD9	9906.54	8331.87	10665.6	9532.76	3893.9	7303.89	6607.62	9526.59	7499.04
O00267	O00267	SPT5H_HUMAN	SUPT5H				1502.93	1182	1917.05	1872.25		991.885
O00273	O00273	DFFA_HUMAN	DFFA				692.896		3882.89	2523.25	753.402	1636.42
O00291	O00291	HIP1_HUMAN	HIP1	13662		17792.1				23983.2		21707.8
O00299	O00299	CLIC1_HUMAN	CLIC1	14550.1	12458.4	17746.5	17088.4	6969.5	12223.5	4454.81	8432.73	5182.42
O00303	O00303	EIF3F_HUMAN	EIF3F	4272.8		4738.85	7454.24	15869.6	4993.07	7994.83	4764.73	6727.04
O00330	O00330	ODPX_HUMAN	PDHX	5677.21	5770.59	11537	42520.1	2196.91	12526.9	2447.96	3144.54	5416.4
O00401	O00401	WASL_HUMAN	WASL			2726.29		1515.66	2548.99			
O00410	O00410	IPO5_HUMAN	IPO5	8699.52	7719.02	8934.75	13480.8	13574	11884.2	16896	15580.6	13953.5
O00411	O00411	RPOM_HUMAN	POLRMT							1999.03	3377.75	2424.91
O00425	O00425	IF2B3_HUMAN	IGF2BP3	16644.8	8259.91	6952.73	21363.3	53328.1	15166.7	7894.5	10635.2	11558.6
O00429	O00429	DNM1L_HUMAN	DNM1L	2968.82	2898.9	3553.2	12562	6211.3	9364.95	7503.02	11527.7	6645.67
O00442	O00442	RTCA_HUMAN	RTCA	3123.49		1998.48	3744.63	7656.63	5334.47	3835.18	3041.31	4743.81
O00443	O00443	P3C2A_HUMAN	PIK3C2A				1482.23	2889.57	2997.3	1888.1	3192.6	2210.53
O00458	O00458	IFRD1_HUMAN	IFRD1					3608.14	1113.98	582.02		808.86
O00461	O00461	GOLI4_HUMAN	GOLIM4				428.6	1318.71	1285.23	2429.94		1970.37
O00463	O00463	TRAF5_HUMAN	TRAF5							1573.31		1964.81
O00469	O00469	PLOD2_HUMAN	PLOD2	2115.63		3384.52	3939.32	2163.27	2048.9	1445.61		
O00471	O00471	EXOC5_HUMAN	EXOC5		1475.61		1137.73	1175.22	999.96	880.86	1227.62	1116.35
O00483	O00483	NDUA4_HUMAN	NDUFA4				7082.09				12563.3	
O00487	O00487	PSDE_HUMAN	PSMD14		5118.68	7171.9	9319.3	9604.98	8372.1	8513.48		8089.47
O00499	O00499	BIN1_HUMAN	BIN1				1313.5		1988.81			
O00505	O00629;O00505	IMA4_HUMAN	KPNA3	3475.59		3723.33	2399.07	3896.12	4162.22	4346.7	1907.67	4590.64
O00515	O00515	LAD1_HUMAN	LAD1							11593	6978.18	9170.3
O00541	O00541	PESC_HUMAN	PES 1.00	3766.79	2731.72	3976.28	2245.37	6641.89	4695.95	4288.35	2923.63	4322.85
O00560	O00560	SDCB1_HUMAN	SDCBP	6617.26	2188.38	5711.24	3664.19	2166.48	2304.33	1453.86	1872.1	1021.85
O00567	O00567	NOP56_HUMAN	NOP56				2789.78	2924.67	1565.92	2383.12	3897	3017.6
O00571	O15523;O00571	DDX3X_HUMAN	DDX3X	20142	19014.1	18357.2	17230.1	25533.1	16556.7	14503.1	9577.19	17348.2
O00629	O00629	IMA3_HUMAN	KPNA4				709.569	1308.13	1667.85	930.608		1102.13
O00712	O00712	NFIB_HUMAN	NFIB	14335.9								
O00743	O00743	PPP6_HUMAN	PPP6C			4532.06	4342.74	5484.19	9067.41	5485.86	4057.95	4567.4
O00763	O00763	ACACB_HUMAN	ACACB	86065.6	71054.2	58448.6	9860.33	32531.4	8561.62	35553.5	83987.9	38319.6
O00764	O00764	PDXK_HUMAN	PDXK						2433.75	1746.28		1749.85
O14493;O15551;O95484;P56747	P56747;O14493;O15551;O95484	CLD3_HUMAN;CLD4_HUMAN;CLD6_HUMA	CLDN3;CLDN4;CLDN6;CLDN9					13975.9	5445.98	20359.1	7569.68	15704.3
O14530	O14530	TXND9_HUMAN	TXNDC9				480.327	498.132	924.613	463.413		731.182
O14531	O14531	DPYL4_HUMAN	DPYSL4								4868.79	
O14556	O14556	G3PT_HUMAN	GAPDHS				2025.96	8059.46				
O14578	O14578	CTRO_HUMAN	CIT	46204	35773	28994.4	28717.8	10127.9	10018.5	16652.3	57241.4	12908.2
O14579	O14579	COPE_HUMAN	COPE	7656.65	8682.54	7353.34	6865.09	8166.43	8064.75	10772.8	9080.09	7934.37
O14617	O14617	AP3D1_HUMAN	AP3D1	4380.25	4248.9		1900.99	6626.9	3154.38	1647.24	1963.33	2705.6
O14639	O14639	ABLM1_HUMAN	ABLIM1					4113.88	1564.03	1535.58		
O14641	O14641	DVL2_HUMAN	DVL2					1180.34		1270.92	895.021	1456.07
O14646	O14647;O14646	CHD1_HUMAN	CHD1				1589.26	5561.17	3699.31	2756.7	1423.24	2564.8
O14653	O14653	GOSR2_HUMAN	GOSR2			2832.07	2752.88	2869.88	3046.59		1362.29	1887.8
O14656	O14656	TOR1A_HUMAN	TOR1A				2110.43	2832.59	2958.18	2326.9	2141.23	1462.86
O14672	O14672	ADA10_HUMAN	ADAM10	5271.8	3312.31	3969.82	4168.95	3083.6	5481.65	4683.1	2224.71	4460.89
O14730	O14730	RIOK3_HUMAN	RIOK3	3032.76		3412.11	5390.04	1395.94	1627.35	600.736	3375.6	4180.88
O14733	O14733	MP2K7_HUMAN	MAP2K7				24317.3	1980.21	1418.78	2042.21	29410.9	2437.59
O14734	O14734	ACOT8_HUMAN	ACOT8		6323.4	6609.79	3198.09	2311.69	1153.47	4923.08	3278.61	4787.81
O14735	O14735	CDIPT_HUMAN	CDIPT	4863.57	3481.37	7059.15	2670.32			2325.28	2627.03	2386.26
O14737	O14737	PDCD5_HUMAN	PDCD5	4392.69	1283.65	3194.75	4894.63	2717.33	8158.67	6315.77	5964.33	5215.84
O14744	O14744	ANM5_HUMAN	PRMT5	4962.45	3349.47	4439.66	5880.71	8683	9110.91	6293.13	4381.72	8652.63
O14745	O14745	NHRF1_HUMAN	NHERF1			2495.22	1688.6			1095.69	1985.12	1149.71
O14757	O14757	CHK1_HUMAN	CHEK1					2269.95	1903.42	3220.42	1701.2	2932.08
O14773	O14773	TPP1_HUMAN	TPP1	13673.2	10126.1	13954.2	9327.64	7845.42	5599.73	9239.7	15675.1	10211.2
O14776	O14776	TCRG1_HUMAN	TCERG1				783.182	2841.58	1659.49	1735.51		1532.14
O14802	O14802	RPC1_HUMAN	POLR3A	11366.7	14767.1	4496.38	2841.61	3869.42	1812.85	2957.99	2156	3019.69
O14818	O14818	PSA7_HUMAN	PSMA7	5149.09	4064.84	5713.71	4539.52	3200.26	6661.39	6187.82	4523.36	7129.77
O14828	O14828	SCAM3_HUMAN	SCAMP3	5408.52		5959.39	6673.64	4858.89	4317.78	3332.9	3668.3	2324.75
O14867	O14867	BACH1_HUMAN	BACH1	2732.88			2800.88	5189.56	3338.27			
O14874	O14874	BCKD_HUMAN	BCKDK				6703.83				5457.57	4776.84
O14879	O14879	IFIT3_HUMAN	IFIT3	3953.55	13220.6	2605.86	4161.43	4749.5	5321			
O14880	O14880	MGST3_HUMAN	MGST3	2252.62	3193.08			1850.44		1933.98	921.773	986.956
O14907	O14907	TX1B3_HUMAN	TAX1BP3						505.253	1076.48	1791.25	
O14908	O14908	GIPC1_HUMAN	GIPC1						2849.86			2068.63
O14929	O14929	HAT1_HUMAN	HAT1					2453.72	4239.52	3536.06		3878.32
O14936	O14936	CSKP_HUMAN	CASK					1978.75	1884.94	4077.95		4607.26
O14939	O14939	PLD2_HUMAN	PLD2		2068.12	2434.97	1110.97	2782.84	2732.55	4656.63	1758.57	4425.35
O14950;P19105	P24844;P19105;O14950	ML12A_HUMAN;ML12B_HUMAN	MYL12A;MYL12B	41506.6	24828.5	31551.4	174838	59453.3	44833.7	60429.1	125830	34444.3
O14964	O14964	HGS_HUMAN	HGS	2907.62		3537.27	4866.64			1819.08	2738.36	
O14965	O14965	AURKA_HUMAN	AURKA				3827.24	4195.65	3364.94	3305.87	2363.86	3446.54
O14966;P57729;Q13637	O14966;P57729;Q13637	RAB32_HUMAN;RAB38_HUMAN;RAB7L_HU	RAB29;RAB32;RAB38			5768.82				3408.1	1708.51	2471.61
O14974	O14974	MYPT1_HUMAN	PPP1R12A	20642.1	18826.5	17394.8	53310.7	101261	65828	8171.44	9258.54	8210.18
O14976	O14976	GAK_HUMAN	GAK				1985.25	4260.46	4315.41	5586.33	3243.34	6607.34
O14979	O14979	HNRDL_HUMAN	HNRNPDL	3526.15			3359.51	16358.7	6802.63	3360.05	902.872	4330.2
O14980	O14980	XPO1_HUMAN	XPO1	15622.3	12410.9	12040.6	19177.3	13389.4	16724.8	25357.8	24647.5	24644.1
O14981	O14981	BTAF1_HUMAN	BTAF1	4363.25	4781.44		5318.13	6762.3	5181.87	5881.61	6146.78	5474.16
O15014	O15014	ZN609_HUMAN	ZNF609				1295.07	2978.85	2315.01	1657.86	931.814	1617.19
O15020	O15020	SPTN2_HUMAN	SPTBN2		582.102			3409.1	2272.09	1969.86	1412.06	1685.69
O15027	O15027	SC16A_HUMAN	SEC16A	4855.08	2501.73	2650.6	3732.41	4969.36	3663.72	4228.3	4824.14	5108.34
O15031	O15031	PLXB2_HUMAN	PLXNB2	4715.85	4665.76		4253.52	4766.83	4229.54	6446.51	5615.57	5525.45
O15042	O15042	SR140_HUMAN	U2SURP	2610.59	3520.24		3699.63	11358.6	4189.32	7810.86	4183.59	7217.95
O15056	O15056	SYNJ2_HUMAN	SYNJ2	17701	13547.4	10728.1	12920.5	8235.77	2959.69	4629.98	12042.2	6325.57
O15061	O15061	SYNEM_HUMAN	SYNM	13261.5	13819.8	10332.5		6972.62	5324.76			
O15067	O15067	PUR4_HUMAN	PFAS			4147.99	6082.59	2990.28	4458.72	8922.15	5485.73	8306.87
O15075	O15075	DCLK1_HUMAN	DCLK1									
O15084	O15084	ANR28_HUMAN	ANKRD28				1792.85	4343.38	3315.23	2383.16	1125.98	2595.6
O15091	O15091	MRPP3_HUMAN	PRORP		3777.78	3788.82	3589.86	5129.01	3403.55	9842.26	9435.54	9818.21
O15111	O15111	IKKA_HUMAN	CHUK	2263.13	2320.82	1731.4	4785.82	5951.48	5857.1	2815.59	2049.57	1929.29
O15117	O15117	FYB1_HUMAN	FYB1					9234.62	884.342			
O15118	O15118	NPC1_HUMAN	NPC1		2948.68			3674.77	3200.66	2800.02	2141.56	5171.42
O15119	O15119	TBX3_HUMAN	TBX3									13055.5
O15127	O15127	SCAM2_HUMAN	SCAMP2	4751.54		3207.47	6820.42	4411.52	5388.34	5095.43	4513.47	4562.47
O15143	O15143	ARC1B_HUMAN	ARPC1B	4559.24	2967.31	6445.17	3305.2	6562.7	11974.4	4764.64	1068.38	5227.15
O15144	O15144	ARPC2_HUMAN	ARPC2	8016.76	10839	13498.9	9497.99	16348.6	33980.9	12623.2	4441.08	14747.2
O15145	O15145	ARPC3_HUMAN	ARPC3		7739.95	11850.5	9264.89	19792	29513.1	11414.2	7028.57	8110.13
O15160	O15160	RPAC1_HUMAN	POLR1C	9254.19	14628.9		2827.93	3372.46	4934.42	5533.96	1595.58	3467.37
O15162	O15162	PLS1_HUMAN	PLSCR1					3382.25	3233.16	2574.73		
O15194	O15194	CTDSL_HUMAN	CTDSPL							2714.67		1442.02
O15212	O15212	PFD6_HUMAN	PFDN6	7746.25	8909.5	6708.77	4701.19	9459.19	7582.66	6629.88	4556.04	6562.19
O15228	O15228	GNPAT_HUMAN	GNPAT				4175.14		3944.62	3144.2	2943.1	4104.39
O15231	O15231	ZN185_HUMAN	ZNF185				340.221		1762.07			
O15234	O15234	CASC3_HUMAN	CASC3					3974.4	1975.34			
O15235	O15235	RT12_HUMAN	MRPS12	2102.42	3024.92	2557.75	2079		1894.12	1950.03	3221.11	1607.72
O15258	O15258	RER1_HUMAN	RER1	1406.45		3378.51	3727.87		1853.13	1816.49	2147.83	2564.23
O15269	O15269	SPTC1_HUMAN	SPTLC1	6938.78	9744.6	8452.25	7814.84	6325.09	5366.9	8531.21	10314.6	9612.88
O15270	O15270	SPTC2_HUMAN	SPTLC2				1535.8	1615.79	1121.79	1482.34	1227.4	1835.79
O15347	O15347	HMGB3_HUMAN	HMGB3	4275.22	3301.59		3146.1	4829.18	3910.73	3456.45	5902.05	3949.43
O15355	O15355	PPM1G_HUMAN	PPM1G				11728.4	10730.2	18571.3	13326.3	13039.7	13267.2
O15360	O15360	FANCA_HUMAN	FANCA					5017.3	2064.88	3956.7	4513.6	4032.64
O15371	O15371	EIF3D_HUMAN	EIF3D	3243.47	3125.91	3661.48	7256.18	6982.51	6570.21	4817.2	3717.84	5674.83
O15372	O15372	EIF3H_HUMAN	EIF3H	2140.52	1394.87	1955.23	4767.32	7294.93	8157.64	8860.91	3101.84	8812.39
O15382	O15382	BCAT2_HUMAN	BCAT2					1225.93	3040.05	1658.43		1593.15
O15397	O15397	IPO8_HUMAN	IPO8	5491.6	3155.94	4494.46	9288.02	2519	3392.59	7843.72	11979.9	10653.3
O15427	O15427	MOT4_HUMAN	SLC16A3	5011.91	4582.26	3886.12	2820.23	5579.28	2211.28	4937.47	5454.36	5320.05
O15439	O15439	MRP4_HUMAN	ABCC4							3471.62	1308.79	2449.29
O15460	O15460	P4HA2_HUMAN	P4HA2				4550.46	9549.09	4336.04	3669.43	3547.85	4961.16
O15479	O15479	MAGB2_HUMAN	MAGEB2				15630.2	37136	19562.3	1804.21	3168.11	2398.32
O15484	O15484	CAN5_HUMAN	CAPN5	1338.34			2416.49	10552.7	8818.7	6115.54	2116.34	6158.58
O15498	O15498	YKT6_HUMAN	YKT6				6012.96	7568.54	4067.1	7262.82	2147.43	9695.11
O15511	O15511	ARPC5_HUMAN	ARPC5	5849.04			4371.72		5742.85		5045.58	
O15523	O15523	DDX3Y_HUMAN	DDX3Y	2926.01	2511.1	1982						
O15525	O60675;O15525	MAFG_HUMAN	MAFG				3623.5	2742.13	2250.8		2091.34	
O15533	O15533	TPSN_HUMAN	TAPBP	3668.28			2371.3	1092.53	1019.3	3740.49	2782.56	3029.9
O43143	O43143	DHX15_HUMAN	DHX15	6098.92	6313.98	6435.8	4859.19	23464	12681.7	10372.9	2775.58	8752.82
O43148	O43148	MCES_HUMAN	RNMT					3415.17	3673.78	1664.72		
O43155	O43155	FLRT2_HUMAN	FLRT2	3904.94	1893.78	4508.89						
O43156	O43156	TTI1_HUMAN	TTI1				1267.68	1660.28	1189.92	1251.36	971.353	1483.41
O43169	O43169	CYB5B_HUMAN	CYB5B				1489.97	8098.14	6250.82	16175.1	3834.98	12357.6
O43172	O43172	PRP4_HUMAN	PRPF4	908.551	1382.62	1868.82	1750.72	3051.14	3632.84	3154.51	1374.48	2985.27
O43175	O43175	SERA_HUMAN	PHGDH	26855.1	26694.4	27201.9	27384.2	27624.6	20730.7	17905.5	20277.4	17673.1
O43237	O43237	DC1L2_HUMAN	DYNC1LI2	5337.77	4403.43	9091.24	6399.85	6893.01	4703.13	5773.72	8258.63	5141.96
O43242	O43242	PSMD3_HUMAN	PSMD3	12265.2	15296.5	12412.7	18662.6	18041.2	15827.9	12553	16867.2	14032.4
O43251	O43251;Q9NWB1	RFOX2_HUMAN	RBFOX2				1723.11		695.832	734.779	1898.76	2315.85
O43252	O43252	PAPS1_HUMAN	PAPSS1			1840.49		1653.12	1979.32			
O43264	O43264	ZW10_HUMAN	ZW10				5825.79	4776.96	2575.49	5594.75	5611.04	5172.73
O43290	O43290	SNUT1_HUMAN	SART1				309.821	1412.98	798.826	981.978	457.141	498.151
O43294	O43294	TGFI1_HUMAN	TGFB1I1				1680.28	4418.1	2510.39			
O43301	O43301	HS12A_HUMAN	HSPA12A							4169.83	4518.89	1194.51
O43324	O43324	MCA3_HUMAN	EEF1E1		1659.29	3687.59	5304.24	2822.13	2230.92	2724.58	3868.22	3396.7
O43353	O43353	RIPK2_HUMAN	RIPK2							1429.27	1431.43	2076.89
O43390	O43390	HNRPR_HUMAN	HNRNPR		3338		2940.32	11880.6	3898.8	3405.09		3971.71
O43395	O43395	PRPF3_HUMAN	PRPF3				7442.3	10617.7	8723.28	4940.75	5664.44	8592.75
O43396	O43396	TXNL1_HUMAN	TXNL1	5411.53		6298.89	1555.87	2211.47	13720.3	2626.83		4235.2
O43399	O43399	TPD54_HUMAN	TPD52L2	9188.54		8739.52	14275.2	5939.43	15568.9	5414.28	4973.61	6695.19
O43427	O43427	FIBP_HUMAN	FIBP							1780.93		1914.46
O43447	O43447	PPIH_HUMAN	PPIH				1749.89	1258.4	1999.24	1360.24		1198.67
O43488	Q8NHP1;O43488	ARK72_HUMAN	AKR7A2			1716.69			2515.86	1746.65	1471.55	2228.94
O43491	O43491	E41L2_HUMAN	EPB41L2	5189.44		5065.13	3350.03	3583.28	4130.15	5301.37	2483.08	5654.66
O43493	O43493	TGON2_HUMAN	TGOLN2				2303.82		635.919		1136.93	
O43502	O43502	RA51C_HUMAN	RAD51C				2529.51			3999.95	7483.3	5460.01
O43542	O43542	XRCC3_HUMAN	XRCC3				1048.35		834.28	2557.68	971.83	2487.95
O43561	O43561	LAT_HUMAN	LAT				1941.24	2691.33	3498.4	7505.79	1859.54	5768.1
O43592	O43592	XPOT_HUMAN	XPOT	1800.72	4920.05	4661.28	7167.46	8953.78	8852.34	6915.87	5603.97	9805.99
O43615	O43615	TIM44_HUMAN	TIMM44			2694.34	6603.41	4328.01	5872.04	7109.2	5667.63	7152.28
O43663	O43663	PRC1_HUMAN	PRC1					2905.16	2138.37	960.875		
O43670	O43670	ZN207_HUMAN	ZNF207				876.172	928.131	1318.43	2241.26	615.663	2089.83
O43681	O43681	GET3_HUMAN	GET3	11058.7	6025.44	9133.85	4458.35		5650.33		6495.83	
O43684	O43684	BUB3_HUMAN	BUB3	4840.72		3566.58	3354.02	6482.33	3876.24	3890.63	2702.35	4427.83
O43707	O43707	ACTN4_HUMAN	ACTN4	21070.6	13234.6	25012.6	30826.6	33311.1	57161.7	37590.2	13721.6	32202.9
O43747	O43747	AP1G1_HUMAN	AP1G1	3949.63	3510.26	3307.29	4765.03	5578.97	5236.7	8532.6	5584.2	7430.38
O43765	O43765	SGTA_HUMAN	SGTA				2252.09	1681.81	2005.62	1912.74		2210.07
O43768	O43768	ENSA_HUMAN	ENSA						1870.93	967.534		874.112
O43776	O43776	SYNC_HUMAN	NARS1	3531.48		6798.85	6486.18	6837.14	15909.4	2933.8	1338.05	3164.56
O43790	O43790	KRT86_HUMAN	KRT86	6742.84		4748.26	6477.45		1156.7	2469.11	4117.86	2217.76
O43795	O43795	MYO1B_HUMAN	MYO1B	27567.7	36685.8	30333.6	22979.1	27406.5	20981.3	81331.7	26118.2	67604.3
O43809	O43809	CPSF5_HUMAN	NUDT21	7012.92	7652.71	6701.72	9683	81458.2	11261	7423.12	8583.36	7381.49
O43815	O43815;Q13033;Q9NRL3	STRN_HUMAN	STRN			1777.88	2069.38	2482.02	3808.94	2390.86		2151.9
O43824	O43824	GTPB6_HUMAN	GTPBP6	2476.07	1921.01	2183.68	1026.41	3282.37	1882.44	2127.74	1110.47	2587.91
O43837	O43837	IDH3B_HUMAN	IDH3B	6536.74	9476.73	4271.27	7151.66	7980.23	5601.73	11715.5	9668.27	9601.76
O43847	O43847	NRDC_HUMAN	NRDC				3491.08	1514.93	3921.01	1740.76		
O43852	O43852	CALU_HUMAN	CALU	15785.5	7550.11	16949.2	17965.6	8165.06	14475.8	4719.59	7774.84	4787.25
O43854	O43854	EDIL3_HUMAN	EDIL3		2252.48	3550.21	6751.96	9253.56	11751.5		6738.18	
O43896	Q12756;O43896;O60333	KIF1C_HUMAN	KIF1C			9236.15						
O43913	O43913	ORC5_HUMAN	ORC5					5379.11	2399.2	3761.33	2535.32	946.475
O43929	O43929	ORC4_HUMAN	ORC4					9181.07	3938.04	4931.71	7496.07	
O60218	O60218	AK1BA_HUMAN	AKR1B10			4840.22						
O60220	O60220	TIM8A_HUMAN	TIMM8A				2929.88		2399.2	2363.32		2152.74
O60231	O60231	DHX16_HUMAN	DHX16					1323.38	843.221	706.645		
O60232	O60232	ZNRD2_HUMAN	ZNRD2				5334.65				4585.28	
O60237	O60237	MYPT2_HUMAN	PPP1R12B					1745.54	1018.99			
O60245	O60245	PCDH7_HUMAN	PCDH7				2282.87	4787.58	5343.93			
O60248	O60248	SOX15_HUMAN	SOX15					731.072				
O60256	O60256	KPRB_HUMAN	PRPSAP2					3443.56	4988.04	2213.14		
O60264	O60264;P28370	SMCA5_HUMAN	SMARCA5			3629.26	3734.11	6729.36	3789.21	2676.73	2641.68	3639.2
O60271	O60271	JIP4_HUMAN	SPAG9				2603.84	2382.29	3453.55	1729.78		1945.46
O60285	O60285	NUAK1_HUMAN	NUAK1	50380		46929.3				5616.05	12788.3	5729.02
O60313	O60313	OPA1_HUMAN	OPA1		2453.12	1754.07	5221.17	4973.4	5245.73	5977.36	4923.58	5707.16
O60315	O60315	ZEB2_HUMAN	ZEB2	2363.46		1581.34						
O60341	O60341	KDM1A_HUMAN	KDM1A					5331.9	3926.81	4698.41	5002.38	3394.28
O60437	O60437	PEPL_HUMAN	PPL	21495.9	10886.2	2853.21		1448.01	657.715	2692.64	1698.3	2150.29
O60476	O60476	MA1A2_HUMAN	MAN1A2				7240.63	1329.56			8817.15	
O60488	O60488	ACSL4_HUMAN	ACSL4	9527.4	9614.64	9827.08	6795.06	6308.35	6572.38	6570.47	4822.45	6084.43
O60493	O60493	SNX3_HUMAN	SNX3			1966.64			2262.73			
O60502	O60502	OGA_HUMAN	OGA			593.911		2013.09	1427.53	1388.69	809.037	2533.69
O60504	O60504	VINEX_HUMAN	SORBS3									
O60506	O60506	HNRPQ_HUMAN	SYNCRIP	9505.59	11838.6	7841.44	7196.71	19784.2	13126.5	9534.64	7289.45	9920.3
O60518	O60518	RNBP6_HUMAN	RANBP6				778.48	9160.2	7311.7	3022.4	1858.59	5518.98
O60547	O60547	GMDS_HUMAN	GMDS			9921.97	3415.65			7386.35	12231.7	8367.56
O60568	O60568	PLOD3_HUMAN	PLOD3				4055.11	4668.38	4815.47	2081.96	5487.53	2268.84
O60573	O60573	IF4E2_HUMAN	EIF4E2				662.284	644.822	568.138	1122.31		
O60609	O60609	GFRA3_HUMAN	GFRA3							5156.39	896.96	6261.15
O60610	O60610	DIAP1_HUMAN	DIAPH1				6642.27	6898.82	6934.24	7576.42	5881.77	7385.93
O60645	O60645	EXOC3_HUMAN	EXOC3	5658.24	7944.96	3903.85	7774.56	5960.97	6878.31	5681.29	4542.56	5881.12
O60656;P[illegible]	O9HAW9;O60656;P22310;P22309;P19224;P35503;P3	UD110_HUMAN;UD11_HUMAN;UD13_HUM/	UGT1A1;UGT1A10;UGT1A3;UGT1A4;UGT1A5;UGT1A6;UGT1A7;UGT1A8;UGT1A9									
O60658	O60658	PDE8A_HUMAN	PDE8A					1139.45	1084.05	2856.67	993.044	3518.25
O60664	O60664	PLIN3_HUMAN	PLIN3	5988.84	2782.85	6294.17	4781.06	6210.43	10487.9	4118.47	1310.73	4329.52
O60678	O60678	ANM3_HUMAN	PRMT3				2244.96	2237.97	4585.53	2124.33	1934.33	2139.73
O60684	O60684	IMA7_HUMAN	KPNA6			2969.1	5035.6		2035.58	3117.17	3421.56	3313.5
O60701	O60701	UGDH_HUMAN	UGDH	9237.86	8027.53	14670.9	4098.05	4002.64	4894.8	5828.44	6066.77	6700.48
O60716	O60716	CTND1_HUMAN	CTNND1	6535.36	6418.93	5667.72	8466.61	12537.8	9757.8	22932.8	11245.1	20792.7
O60732	O60732	MAGC1_HUMAN	MAGEC1				12771.3	10223.6	11676.3			
O60739;P41567	O60739;P41567	EIF1B_HUMAN;EIF1_HUMAN	EIF1;EIF1B				1662.95	1449.97	2793.07	1379.23		
O60749	Q13596;O60749	SNX2_HUMAN	SNX2	4313.9		3577.66	6850.83	3439.85	4756.07	2311.73	2431.77	2825.08
O60762	O60762	DPM1_HUMAN	DPM1	19841	58012	26301.4	9662.15	13914.5	11688	20452.7	15785.9	17067
O60763	O60763	USO1_HUMAN	USO1	5261.58	3335.5	4672.54	5321.65	5624.19	6431.3	3875.4	3872.71	3217.04
O60826	O60826	CCD22_HUMAN	CCDC22	1634.05	1275.28	1093.99		9245.23	4391.55	1263.46	1156.29	940.325
O60828	O60828	PQBP1_HUMAN	PQBP1				607.293	5173.39	6328.25	3466.57		3509.75
O60831	O60831	PRAF2_HUMAN	PRAF2				2184.56					
O60832	O60832	DKC1_HUMAN	DKC1		2350.83	1364.25	999.132	6221.86	2269.33	5740.06	3581.8	2532.19
O60841	O60841	IF2P_HUMAN	EIF5B	15244.1	25088.4	13659.9	14322	22441.8	15262	15196.4	10162	13414.7
O60869	O60869	EDF1_HUMAN	EDF1				3273.16	1231.1	3583.88		1974.63	1611.26
O60884	O60884	DNJA2_HUMAN	DNAJA2				11058.7	7185.23	7393.12	11645.5	17906	12611.6
O60888	O60888	CUTA_HUMAN	CUTA	15657.5	5545.9	11201		6326.9	26811.5	17700.5		
O60907;Q9BZK7	O60907;Q9BZK7	TBL1R_HUMAN;TBL1X_HUMAN	TBL1X;TBL1XR1			3840.75	967.42	1091.95	1112.26	2094.89		1153.83
O60911	O60911	CATL2_HUMAN	CTSV				2097.67				2345.38	2195.74
O75027	O75027	ABCB7_HUMAN	ABCB7			2576.27	2167.35			2885.78	5253.18	2844.6
O75044	P0DJJ0;O75044;P0DMP2	SRGP2_HUMAN	SRGAP2				964.93		1126.05		1037.39	3084.41
O75083	O75083	WDR1_HUMAN	WDR1	20624.6	16300.2	22133.9	16157.5	21431.6	28422.3	17005	9429.91	11701.4
O75122	O75122	CLAP2_HUMAN	CLASP2		1325.83		7756.97	5114.92	3768.4	2460.89	3794.43	3820.16
O75127	O75127	PTCD1_HUMAN	PTCD1					1361.26		1287.44	3272.77	2686.8
O75131	O75131	CPNE3_HUMAN	CPNE3	7343.21	4752.94	6488.19	5418.57	3185.55	8875.12	5202.62	5225.39	5596.91
O75145	O75145	LIPA3_HUMAN	PPFIA3			3426.13			33279.9	17879.3		13955.2
O75146	O75146	HIP1R_HUMAN	HIP1R				4911.53	5839.06	7564.24	4604.51		5411.72
O75147	O75147	OBSL1_HUMAN	OBSL1			278.682	524.741	435.872	372.121	496.549		628.858
O75150	Q5VTR2;O75150	BRE1B_HUMAN	RNF40				1779.86		2369.69	2697.51		
O75153	O75153	CLU_HUMAN	CLUH				3818.19		2387.51	3884.78	3335.83	4281.57
O75165	O75165	DJC13_HUMAN	DNAJC13	5509.18	6802.85	4108.07	3444.5	4159.6	3391.53	10953.4	5542.56	7345.49
O75175	O75175	CNOT3_HUMAN	CNOT3			1178.82	1717.03	448.627	960.663	1169.1	830.318	895.94
O75179	O75179;Q8IWZ3	ANR17_HUMAN	ANKRD17				4157.79	3738.54	2913.09	3040.23	5368.29	3008.81
O75190	O75190	DNJB6_HUMAN	DNAJB6				6285.85	6061.25	4110.42	2504.84	9767.85	3158.91
O75223	O75223	GGCT_HUMAN	GGCT	15986.4		8372.96	2777.07		4741.22	3283.16	3063.09	3496.86
O75306	O75306	NDUS2_HUMAN	NDUFS2	3411.93		2875.94	3049.08	3274.92	3589.59	7742.41	3510.63	5076.65
O75312	O75312	ZPR1_HUMAN	ZPR1					4375.56	2724.34	1841.12		
O75323	O75323	NIPS2_HUMAN	NIPSNAP2	6978.62		6617.94	5254.77	6842.83	2955.99	6281.92	5202.53	6744.26
O75326	O75326	SEM7A_HUMAN	SEMA7A				1401.36	3225.32	2641.78	1519.67		1099.97
O75340	O75340	PDCD6_HUMAN	PDCD6	3213.34		3743.6	3565.23	5157.15	4277.52	5323.73	3339.74	5376.76
O75342	O75342	LX12B_HUMAN	ALOX12B	20817.3	22403.3	20928.1	6764.29	6809.42	4573.58	5107.91	6082.18	7578.62
O75347	O75347	TBCA_HUMAN	TBCA				2164.13		8986.57	3580.82	2915.73	4329.1
O75348	O75348	VATG1_HUMAN	ATP6V1G1	1160.82			1942.92	1948.9	2426.06	1991.95	1219.15	1427.47
O75351	O75351	VPS4B_HUMAN	VPS4B	3119.63		2489.43	2102.97	1387.45	2840.57	1261.13	394.191	1277.12
O75352	O75352	MPU1_HUMAN	MPDU1				519.436	3185.44	3469.98	3987.46	1719.23	2747.92
O75362	O75362	ZN217_HUMAN	ZNF217	1448.95	823.531	1299	898.84	1323.85	1304.97	647.737		778.156
O75367	O75367	H2AY_HUMAN	MACROH2A1	6574.3	6483.17	3746.61	3114.4	5039.6	4890.77	756.096	2868.11	
O75368	O75368	SH3L1_HUMAN	SH3BGRL				509.066	821.661	2047.86	704.659	421.717	438.151
O75369	O75369	FLNB_HUMAN	FLNB	17470.2	11641.3	16968.8	26910.1	28320.2	27935.1	15186.8	8875.2	13978.3
O75380	O75380	NDUS6_HUMAN	NDUFS6	801.999			238.506	223.895	240.94	1023.18	516.816	587.344
O75381	O75381	PEX14_HUMAN	PEX14							2443.29		2406.22
O75390	O75390	CISY_HUMAN	CS	9025.2		9957.44	24082.9		14682.2	19427.1	22149.1	26097.1
O75394	O75394	RM33_HUMAN	MRPL33	2097.47		3599.1	939.328		1162.04	1148.75	2112.27	1589
O75396	O75396	SC22B_HUMAN	SEC22B	6799.58	9734	4632.11	3123.9	3033.36	4325.84	2259.95	3580.82	2190.79
O75400	O75400	PR40A_HUMAN	PRPF40A					3515.27	1438.97	1099.6		1127.87
O75419	O75419	CDC45_HUMAN	CDC45							1364.12		
O75420	O75420	GGYF1_HUMAN	GIGYF1					7197.38				
O75427	O75427	LRCH4_HUMAN	LRCH4				2867.44	7719.25	5258.19	3480.59		3027.51
O75436	O75436	VP26A_HUMAN	VPS26A	3642.38		4284.06	3342.12	1125.89	2538.61	1669.86	2759.44	1979.98
O75439	O75439	MPPB_HUMAN	PMPCB	6186.88	3786.68	6286.5	6289.21	2887.08	5941.35	4997.1	7579.51	5580.85
O75475	O75475	PSIP1_HUMAN	PSIP1	6081.34				4135.73	3424.09			1824.36
O75477	O75477	ERLN1_HUMAN	ERLIN1		1748.32	2278.66	683.217			1748.44		1981.66
O75489	O75489	NDUS3_HUMAN	NDUFS3	689.961			2107.23	3943.49	3757.04	5209.43	3259.9	5513.34
O75494	O75494	SRS10_HUMAN	SRSF10	1671.59		1958.47		4965.67	1949.5	2110.74	1130.73	1780.72
O75508	O75508	CLD11_HUMAN	CLDN11	1527.21	2134.62	1433.01				2058.28	628.57	1810.96
O75521	O75521	ECI2_HUMAN	ECI2						1172.04	1212.1		1031.72
O75531	O75531	BAF_HUMAN	BANF1	7005.17	20103.2	5137.56	1500.71	10717.2	4011.3	2610	4198.31	6449.46
O75533	O75533	SF3B1_HUMAN	SF3B1	7271.98	10114.8	5278.2	5626.87	18657	8103.48	8665.09	6188.98	8949.92
O75534	O75534	CSDE1_HUMAN	CSDE1	2254.17		3806.61	2264.46	1490.31	1835.07	3762.19	1661.55	3639.16
O75556	O75556	SG2A1_HUMAN	SCGB2A1						798.029		3577.86	
O75569	O75569	PRKRA_HUMAN	PRKRA			18089.4	1297.65	3484.95	1650.77	3236.93	4381.68	2182.56
O75592	O75592	MYCB2_HUMAN	MYCBP2				2334.97			2333.48	4506.13	2416.46
O75600	O75600	KBL_HUMAN	GCAT		787.94		439.518	2008.11	1053.37	1078.34	2565.42	2397.95
O75608	O75608	LYPA1_HUMAN	LYPLA1			3724.11	3367.39	7448.24	9770.77	4780.11	2734.01	5061.32
O75616	O75616	ERAL1_HUMAN	ERAL1	3923.46	7093.01	3427.61	4745.96	3330.38	3446.55	6875.43	5628.19	7094.58
O75635	O75635	SPB7_HUMAN	SERPINB7			4586.81	2918.12	2134.2	2725.4	2071.89	2301.74	3258.99
O75638	O75638	CTAG2_HUMAN	CTAG2			577.904	19913.5	19014.1	17298.1		1843.73	
O75643	O75643	U520_HUMAN	SNRNP200	6959.93		4824.12	7426.42	9794.72	7796.33	8520.61	9841.26	8375.62
O75663	O75663	TIPRL_HUMAN	TIPRL						1173.09			977.302
O75665	O75665	OFD1_HUMAN	OFD1							1409.46		2519.94
O75676	O75676	KS6A4_HUMAN	RPS6KA4		9994.85	8912.44		1533.15	1619.85	5563.3	6853.05	6266.31
O75694	O75694	NU155_HUMAN	NUP155			17647.6	15696.6	10780.7	11448.5	16850.7	16239.4	18828.2

O75695	O75695	XRP2_HUMAN	RP2	9059.18	1829.37	1477.7	13886.9	42124.6	39828.1	81007.5	17446.3	80400.5
O75718	O75718	CRTAP_HUMAN	CRTAP	4571.42			4561.21	14439.8	12945.9	3162.6	1365.41	3453.46
O75746	O75746	S2512_HUMAN	SLC25A12	4131.87				2751.12	1592.18	2440.46		672.836
O75762	O75762	TRPA1_HUMAN	TRPA1	13103.2	19556.8	16005.3						
O75781	O75781	PALM_HUMAN	PALM				3004.67	4033.25	4257.27	3189.75	5083.58	3950.54
O75787	O75787	RENR_HUMAN	ATP6AP2					2813.81	2393.24	3664.89	962.937	1134.6
O75794	O75794	CD123_HUMAN	CDC123	4836.42			6751.68	5093.65	5461.12	4581.93	6004.44	4853.07
O75821	O75821	EIF3G_HUMAN	EIF3G	13739.2			12696.7	15719.6	22006.2	13640.7	6222.48	10592.4
O75822	O75822	EIF3J_HUMAN	EIF3J				8500.48	8936.67	26570.7	9208.7	4529.09	9156.75
O75832	O75832	PSD10_HUMAN	PSMD10	2856.44	1519.98	3165.98	7777.99	5825.73	9847.96	11818.8	9392.86	12313.6
O75843	O75843	AP1G2_HUMAN	AP1G2				5751.3	6848.32	5854.61	7853.39	6322.95	7267.41
O75844	O75844	FACE1_HUMAN	ZMPSTE24					2831.47	2184.26	2983.81		1792.05
O75874	O75874	IDHC_HUMAN	IDH1	6308.13	3326.2	6750.09	4507.11	6005.85	13143.1	4761.11	2070.22	4566.67
O75879	O75879	GATB_HUMAN	GATB				4417.09		4485.05	5264.2	4735.85	6096.95
O75880	O75880	SCO1_HUMAN	SCO1			3655.76	2777.33	3806.97	2640.97	6639.12	2133.84	7212.59
O75891	O75891	AL1L1_HUMAN	ALDH1L1				4892.14	8573.55	7074.11			
O75909	O75909	CCNK_HUMAN	CCNK		1801.69		748.933	1057.13	1939.99	2301.16	405.565	2337.58
O75914	O75914	PAK3_HUMAN	PAK3			8628.35	6641.57					
O75923	O75923	DYSF_HUMAN	DYSF				4309.56	6128.93	5232.61			
O75947	O75947	ATP5H_HUMAN	ATP5PD			1896.7			1408.6	1813.73		2068.81
O75955	O75955	FLOT1_HUMAN	FLOT1	9239.59	7901.29	7281.54	11873.6	13141.8	12022.8	60094.6	22808.5	45075.9
O75962	O75962	TRIO_HUMAN	TRIO	1868.35	1900.56		1172.61	2698.43	3010.46	1626.71		1864.98
O75964	O75964	ATP5L_HUMAN	ATP5MG	4652.86	3667.34	5346.58	1143.12	1419.05	1282.77	2642.32	2283.73	2183.83
O76003	O76003	GLRX3_HUMAN	GLRX3			39889.6	4381.1		8771.72		4174.76	
O76009	O76009	KT33A_HUMAN	KRT33A			3018.3						2763.37
O76011	O76011;A0A140TA62	KRT34_HUMAN	KRT34				4575.49		1783.84	2005.11	1920.13	3180.81
O76013	O76013	KRT36_HUMAN	KRT36				3016.8			3038.26		949.719
O76021	O76021	RL1D1_HUMAN	RSL1D1				284.435	2317.25	1219.63	503.616		684.998
O76024	O76024	WFS1_HUMAN	WFS1	2615.23	2226.68	2116.52				11093.3	3471.06	10363.4
O76031	O76031	CLPX_HUMAN	CLPX	7377.51	6732.99	7900.88	9770.99	6785.76	6153.52	7411.3	12910.1	8984.89
O76070	O76070	SYUG_HUMAN	SNCG				5686.35	1735.65	2042.78			
O76094	O76094	SRP72_HUMAN	SRP72	11115.3	9982.42	7377.06	12562.6	8948.11	11787.7	10152.4	6925.61	10015.6
O94760	O94760	DDAH1_HUMAN	DDAH1			2090.38	5100.52	3814.47	13758.8	2205.78		1854.84
O94766	O94766	B3GA3_HUMAN	B3GAT3						1394.57	2262.63		1652.3
O94776	O94776	MTA2_HUMAN	MTA2	3173.48	3150.3	3373.28	5267.74	5126.94	5725.25	13377.1	5608.68	11732
O94804	O94804	STK10_HUMAN	STK10				1264.99	1875.68	1736.75			1023.71
O94808	O94808	GFPT2_HUMAN	GFPT2					4496.74	3116.99			
O94822	O94822	LTN1_HUMAN	LTN1		742.174	991.359	2656.55	5013.81	4151.63	1725.84	2748.25	1952.36
O94826	O94826	TOM70_HUMAN	TOMM70	1702.92	1743.58	1362.26	1465.38	4332.71	4355.69	3239.69	1258.97	4453.17
O94829	O94829	IPO13_HUMAN	IPO13					776.751	1592.23	2008.37	764.518	2907.73
O94832	O94832	MYO1D_HUMAN	MYO1D						2364.53	27954.2	8208.35	19237
O94842	O94842	TOX4_HUMAN	TOX4				3296.47	1566.3	1888.53	1963.81	5552.84	2578.95
O94851	O94851	MICA2_HUMAN	MICAL2	3329.7			2122.43					
O94874	O94874	UFL1_HUMAN	UFL1			7529.99	2802.22	2490.59	1589.44	2924.59	3140.34	4860.81
O94903	O94903	PLPHP_HUMAN	PLPBP					517.738	1052.35			
O94905	O94905;O75477	ERLN2_HUMAN	ERLIN2	7676.07	11334.7	10593.6	6521.2	12198.1	8073.82	13873	4604.85	11621.8
O94906	O94906	PRP6_HUMAN	PRPF6	8045.67	7115.98	7206.16	12609	8362.61	5649.59	9927.43	18329.4	10261.6
[illegible]	[illegible]	[illegible]	[illegible]	[illegible]	[illegible]	[illegible]	[illegible]	[illegible]	[illegible]	[illegible]	[illegible]	[illegible]

**P10606**	P10606	COX5B_HUMAN	COX5B	5981.26	4414.03	3940.98	3645.85	3921.09	2991.24	8364.69	4655.5	6707.73
**P10619**	P10619	PPGB_HUMAN	CTSA	14660.6	12002.4	15208.9	2639.2	2052.74	3306.89	3074.01	3524.2	2755.07
**P10620**	P10620	MGST1_HUMAN	MGST1	30246.5	37914.6	10522.4	5636.99			7929.98	4085.5	4472.72
**P10644**	P10644	KAP0_HUMAN	PRKAR1A	9760.26	7253.14	5329.82	6274.02	9084.37	10036	4975.71	4120.59	4070.77
**P10768**	P10768	ESTD_HUMAN	ESD				427.727	1194.33	5240.63	968.304		511.114
**P10809**	P10809	CH60_HUMAN	HSPD1	41930	19469.7	41445.2	38434.5	60500.8	76918	93134.8	54715.9	89176.9
**P11021**	P11021	BIP_HUMAN	HSPA5	106636	90909.3	78737.6	58153.4	53391.4	73334.5	41229.7	27448.7	39403.6
**P11047**	P11047	LAMC1_HUMAN	LAMC1	974.499			2300.9	878.835	1601.4	443.282		494.865
**P11142**	P11142	HSP7C_HUMAN	HSPA8	156316	107906	129805	186851	166579	228143	179685	141626	184948
**P11166**	P11166	GTR1_HUMAN	SLC2A1	7596.57	9325.57	9012.05	13163.8	61458.6	35778.4	130824	33225	124843
**P11169**	P11169	GTR3_HUMAN	SLC2A3		1051.08			7308.73	5503.88	10974.9	4951.9	8732.2
**P11171**	P11171	EPB41_HUMAN	EPB41						1581.61	3670.11		3643.13
**P11172**	P11172	UMPS_HUMAN	UMPS	14162.6	10749.2	13210.6	19778.7	11951.4	12356.4	16869.7	26692.2	18469.3
**P11177**	P11177	ODPB_HUMAN	PDHB	2878.04	5109.04	3945.96	9363.83	9080.86	16492.6	8813.94	4628.33	5560.68
**P11182**	P11182	ODB2_HUMAN	DBT	649947	678064	711581	847509	234182	225402	396252	896432	431924
**P11216**	P11217;P11216	PYGB_HUMAN	PYGB	8277.23	9104.81	10772.9	13409.7	15762.7	17341.1	13496.7	7523.3	12554.5
**P11233**	P11233	RALA_HUMAN	RALA			5231.74	6876.61	14996.5	10959	33534.7	7046.8	30846.1
**P11234**	P11234	RALB_HUMAN	RALB				2298.69	5835.74	5862.56	10785.1	2755.49	9204.1
**P11274;Q12979**	P11274;Q12979	ABR_HUMAN;BCR_HUMAN	ABR;BCR			1809.18	2907.08	1821.18	3629	4441.15	1727.09	5047.27
**P11279**	P11279	LAMP1_HUMAN	LAMP1	9652.49	11247.3	12406.9		6520.8	6511.41	10352.7	3466.75	11758.3
**P11310**	P11310	ACADM_HUMAN	ACADM	10959.8	8869.14	9937.42	11499.2	12801.9	8133.95	14886.7	21630.4	14359.1
**P11387**	P11387	TOP1_HUMAN	TOP 1.00	9300.89	8237.48	6828.78	6260	7010.05	4323.38	1699.04	4219.86	4208.87
**P11388**	P11388	TOP2A_HUMAN	TOP2A	2238.54	2407.53		2481.12	4261.19	1881	2184.56	3675.84	2085.8
**P11413**	P11413	G6PD_HUMAN	G6PD	16961.9	11395.5	16270.5	9176.06	9623.3	11470	10711.7	6864.94	11045
**P11441**	P11441	UBL4A_HUMAN	UBL4A	2327.78		2805.33	4244.42	2193.9	4688.59	2938.39	2283.97	3327.87
**P11498**	P11498	PYC_HUMAN	PC	878776	677136	936922	837446	201687	193865	869626	2.25502E+06	1.02646E+06
**P11586**	P11586	C1TC_HUMAN	MTHFD1	16139.1	12497.9	16740.3	19119.6	19287.5	19687.1	22656.8	21967.3	22747.4
**P11717**	P11717	MPRI_HUMAN	IGF2R	3477.07	4427.96	4889.73	5812.23	4276.06	4991.91	1917.23	761.931	1915.44
**P11766**	P11766	ADHX_HUMAN	ADH5					1334.89	2717.95	1867.58		2467.42
**P11802**	P11802	CDK4_HUMAN	CDK4	17248.5	22879.8	16841.1	6908.35	6969.94	4313.57	9319.78	6345.04	7307.9
**P11908**	P11908	PRPS2_HUMAN	PRPS2				3143.35	4245.02	5022.51	9122.92	7606.6	10867.8
**P11940**	P11940;Q4VXU2	PABP1_HUMAN	PABPC1	127197	66819.7	31535.9	69952.2	139785	41130.3	50266.4	54320.7	45071.4
**P12004**	P12004	PCNA_HUMAN	PCNA			4797.39	4954.96		4103.6	4414.78	5434.26	4071.35
**P12035**	P12035	K2C3_HUMAN	KRT3			29306.8	29393.1					
**P12081**	P12081;P49590	HARS1_HUMAN	HARS1	4814.18	2298.39	5992.15	5896.19	6831.98	12988.7	3857.14	3213.77	6810.39
**P12235**	P12235	ADT1_HUMAN	SLC25A4						3049.41	2749.37		7602.74
**P12259**	P12259	FA5_HUMAN	F5							31761.4	9998.72	32997.4
**P12268**	P12268	IMDH2_HUMAN	IMPDH2	80248.2	233087	15314	159566	339673	30320.4	26554	26620.2	23957.7
**P12270**	P12270	TPR_HUMAN	TPR				1453.23	2687.55	2022.11	3432.78	1368	3386.05
**P12273**	P12273	PIP_HUMAN	PIP	30863.2	21090.9	44742.9	16877.8	5838.39	11304.6	5639.04	20774.4	13161.2
**P12429**	P12429	ANXA3_HUMAN	ANXA3	2847.76		3601.93	3240.13	3190.83	6442.71	3796.92	2301.92	3003.84
**P12532**	P12532	KCRU_HUMAN	CKMT1B							1979.63	1322.01	1886.98
**P12694**	P12694	ODBA_HUMAN	BCKDHA	14827.6	14339.3	15111.2	7810.49	6493.4	6510.54	13153.8	17782.3	16411.2
**P12814**	P12814	ACTN1_HUMAN	ACTN1	15272.5	28797	16241.5	30504.2	33421.6	33422.6	17456.2	10082.1	15914.8
**P12830**	P12830	CADH1_HUMAN	CDH1							23423.7	5125.81	24370.5
**P12882**	Q9UKX2;P12882	MYH1_HUMAN	MYH1			624200				890616	151882	726646
**P12883**	P12883	MYH7_HUMAN	MYH7		5219.38	3311.19	11586.1	6968.93			6924.32	1580.22
**P12931**	P12931	SRC_HUMAN	SRC					4888.18	2128.85	4275.84	1442.86	3105.02
**P12955**	P12955	PEPD_HUMAN	PEPD	3047.04	1500.88	2670.79	1266.38	3427.58	8367.06	3124.95	2305.75	3874.36
**P12956**	P12956	XRCC6_HUMAN	XRCC6	#########	#########	71058.6	1.16938E+06	963237	54267	59284.1	738105	37069.6
**P12980**	P12980	LYL1_HUMAN	LYL1									1642.48
**P13010**	P13010	XRCC5_HUMAN	XRCC5	#########	#########	59401.5	2.58516E+06	1.30128E+06	68779.2	86000.9	878735	38170.5
**P13073**	P13073	COX41_HUMAN	COX4I1				5291.92			3260.81	10962.3	2954.05
**P13473**	P13473	LAMP2_HUMAN	LAMP2	11839.8	7002.57	18762.8	27394.6	11449.8	68214.3	15863	17494	11655.6
**P13489**	P13489	RINI_HUMAN	RNH1	14343.2		16197	11202.4	15646.8	10938.3	12696.5	7896.93	6991.28
**P13533**	P13533	MYH6_HUMAN	MYH6									
**P13639**	P13639	EF2_HUMAN	EEF2	30585.2	16016.7	38516.4	36833.5	41461.2	72337.7	31265.1	20174.1	29762.8
**P13645**	P13645	K1C10_HUMAN	KRT10	#########	1.2903E+06	2.54851E+06	929853	288513	615105	666364	1.07599E+06	1.17443E+06
**P13646**	P13646	K1C13_HUMAN	KRT13	19746.6		63235.9	55645.7		15801.4	26582.7	159689	41639.3
**P13647**	P13647	K2C5_HUMAN	KRT5	243128	213336	421874	177914	65895.4	117958	130626	289311	254902
**P13667**	P13667	PDIA4_HUMAN	PDIA4	10455		10918.9	5542.05	5648.81	9774.61	8915.3	5953.13	7737.52
**P13674**	P13674	P4HA1_HUMAN	P4HA1		4927	4622.75	7244.87	10316.5	7432.9	6009.32	7672.76	6340.85
**P13688**	P13688	CEAM1_HUMAN	CEACAM1				394.126	1098.52	979.323	6181.28	627.218	4756.24
**P13693**	Q56UQ5;P13693	TCTP_HUMAN	TPT1	3574.35		5603.8	8262.6	7594.8	26085	3736.33	2289.76	3939.68
**P13716**	P13716	HEM2_HUMAN	ALAD									
**P13796**	P13796	PLSL_HUMAN	LCP1			6349.2	25865.3	10536.7	31129.5	11970.7	10328.6	15466.8
**P13797**	P13797	PLST_HUMAN	PLS3	10270.5	8359.49	12350.9	12417.2	11519.6	20312	10116.6	5139.23	11157.2
**P13798**	P13798	ACPH_HUMAN	APEH		1188.36	3474.38	2565.28	3484.91	5640.55	3619.2	3649.23	2545.6
**P13804**	P13804	ETFA_HUMAN	ETFA	21383	28765.2	28346.2	46459.6	37995.1	20752.6	41180.7	73812.9	33301.4
**P13805**	P13805	TNNT1_HUMAN	TNNT1									
**P13807**	P13807	GYS1_HUMAN	GYS1			2866.77	2198.32	3687.68	4040.46	1556.09		1714.64
**P13861**	P13861	KAP2_HUMAN	PRKAR2A				3810.19	2238.89	916.844	1271.16	3306.35	1792.84
**P13929**	P13929	ENOB_HUMAN	ENO3	139768	16907.7	53883.4	33270.2	24792.7	68655.7	66422.2	29662.8	40281.2
**P13984**	P13984	T2FB_HUMAN	GTF2F2	3165.13		3065.26		3614.38	3869.13	2580.66		
**P13987**	P13987	CD59_HUMAN	CD59		5332		15892.2	10812.6	17833.1	11637.9	8103.38	11509.4
**P13995**	P13995	MTDC_HUMAN	MTHFD2					1876.05	4878.17	1888.44		1704.59
**P14174**	P14174	MIF_HUMAN	MIF	71669.8	8601.04	72182	32067	12951.1	52692.6	39950.1	23879.5	37806.1
**P14222**	P14222	PERF_HUMAN	PRF1							5536.87	4082.71	9153.35
**P14314**	P14314	GLU2B_HUMAN	PRKCSH	9475.1	5569.56	8352.7	11505.6	8583.65	16873.8	12183.5	6735.69	10537.8
**P14324**	P14324	FPPS_HUMAN	FDPS	2665.44			4211.04		3012.72	2481.64	1807.24	1892.91
**P14373**	P14373	TRI27_HUMAN	TRIM27	4307	3974.92	4110.77	3102.89	15451.3	10229	22037.7	1926.28	27576.6
**P14550**	P14550	AK1A1_HUMAN	AKR1A1	8515.65	4324.68	8244.49	4652.03	7897.43	23330.7	2990.51	2084.05	2471.86
**P14618**	P14618	KPYM_HUMAN	PKM	59052.8	34428.9	71617.9	38734.9	44805.7	102659	58694.5	30082.1	54764.4
**P14625**	P14625	ENPL_HUMAN	HSP90B1	22574.4	13685.1	25051.8	19879.3	19385	28201.5	22107.7	13038.9	24809.7
**P14635**	P14635	CCNB1_HUMAN	CCNB1				910.593	6508.11	4581.41	2924.82	1593.52	1813.56
**P14649**	P14649	MYL6B_HUMAN	MYL6B	1009.17			5067.23	8093.32	4657.53	2916.36	3219.34	2088
**P14678;P63162**	P14678;P63162	RSMB_HUMAN;RSMN_HUMAN	SNRPB;SNRPN	5511.75	5097.43	4537.86	8556.9	26703.4	4558.3	13911.5	13379.2	10994.5
**P14735**	P14735	IDE_HUMAN	IDE	6702.63		6928.77	5788.12	7831.95	6127.72	7825	5796.06	6853.53
**P14866**	P14866	HNRPL_HUMAN	HNRNPL	4086.7		2818.09	5114.41	4238.57	2015.72	1625.51	2760.24	1579.54
**P14868**	P14868	SYDC_HUMAN	DARS1	13883.5	13301.9	11743.4	17802.7	15694.6	15734.5	15302	14652.4	15711
**P14923**	P14923	PLAK_HUMAN	JUP	44022.4	25699.2	52680.1	42969.4	39111.3	34457.5	49110.8	28189.9	48770.2
**P15104**	P15104	GLNA_HUMAN	GLUL							2490.84	1011.53	3222.36
**P15121**	P15121	ALDR_HUMAN	AKR1B1		3141.22	9260.22		3274.98	12933.4	2945.3	1826.54	
**P15144**	P15144	AMPN_HUMAN	ANPEP			2410.66	8981.56	9130.85	9563.84			
**P15151**	P15151	PVR_HUMAN	PVR	1037.22	721.53	959.482	938.239	3997.88	4213.58	6299.78	616.605	5072.08
**P15153**	P15153	RAC2_HUMAN	RAC2				8949.07	2532.43	10510.1	2612.96	2443.71	3888.41
**P15170**	P15170	ERF3A_HUMAN	GSPT1	3833.72	5664.08	6277.32	6521.29	10569.6	18797.9	5941.83	2792.23	6409.37
**P15259**	P15259	PGAM2_HUMAN	PGAM2						3173.61			
**P15311**	P15311	EZRI_HUMAN	EZR	7977.8		7940.05	14161.9	16869.3	26902.4	15633.5	5885.84	16581.8
**P15328**	P15328	FOLR1_HUMAN	FOLR1				3988.99	4199.52	3281.18	10065	3815.76	10569.8
**P15428**	P15428	PGDH_HUMAN	HPGD									
**P15531**	P15531;O60361;P22392	NDKA_HUMAN	NME1	59830.9	27156.5	70785	95274.5	66436.9	164634	59791.9	36214.5	65493.9
**P15559**	P15559	NQO1_HUMAN	NQO1	37280.9	26643.2	37389.9	8555.57	2789.99	5997.09	27602.8	16380.2	23630.5
**P15586**	P15586	GNS_HUMAN	GNS	3724.16		4243.54	3324.88		2056.39	5838.07		
**P15880**	P15880	RS2_HUMAN	RPS2	53424.2	63406.3	50904.9	38783.9	78044.2	57476.2	53888.9	31317.4	44499.3
**P15882**	P15882	CHIN_HUMAN	CHN1				1116.25	3215.21	2383.38	956.531		
**P15924**	P15924	DESP_HUMAN	DSP	53308.1	35194	64957.2	33248.5	19603.9	17122.4	78713.5	42105.5	66004.6
**P15927**	P15927	RFA2_HUMAN	RPA2	35134.2	270012	7690.69	17096.2	20042	6534.95	7244.27	32237.8	
**P16070**	P16070	CD44_HUMAN	CD44	14484.8	12313.4	9545.94	10283.5	20992.4	36936.1	5992.02	3203.61	4496.09
**P16144**	P16144	ITB4_HUMAN	ITGB4		7707.62	7020.25		4581.24		59085.3	21066	44451.9
**P16152**	P16152	CBR1_HUMAN	CBR1	4006.38	1999.68	3478.83	2125.36	731.656	1432.58	790.838		
**P16219**	P16219	ACADS_HUMAN	ACADS						587.55			
**P16220**	P16220	CREB1_HUMAN	CREB1									
**P16278**	P16278	BGAL_HUMAN	GLB1	45296.7	48301.3	51196.6	1316.26	7332.89	8099.68	6208.68	2048.22	5264.44
**P16401**	P16401	H15_HUMAN	H1-5	14699.7	12986.8	8827	3176.05	6526.61	3923.23	3271.92	2774.61	2225.05
**P16403**	P16402;P10412;P16403	H12_HUMAN	H1-2	37272.2	36915.1	21431.9	19200.8	17928.9	9869.72	14828.5	15650.9	9484.85
**P16422**	P16422	EPCAM_HUMAN	EPCAM							10901.7	6543.87	9835.9
**P16435**	P16435	NCPR_HUMAN	POR						435.254	1161.72		1770.03
**P16444**	P16444	DPEP1_HUMAN	DPEP1							24651.1	8775.08	18450.2
**P16615**	Q93084;P16615	AT2A2_HUMAN	ATP2A2	9002.81	7797.77	11675.5	10775.6	7126.2	5950.71	11530.5	11704.9	12749.9
**P16671**	P16671	CD36_HUMAN	CD36				6253.56				5094.12	
**P16930**	P16930	FAAA_HUMAN	FAH			2179.32	1360.58	3387.18	8703.69	1621.85		988.45
**P16949**	P16949	STMN1_HUMAN	STMN1	12149	4309.68	13158.9	12720.9	9919.77	11986.7	13541	10831.6	16182.5
**P16989**	P16989	YBOX3_HUMAN	YBX3	4762.3	6756.51	1505.28	5801.88	4979.2	3822.43	3318.26	7910.33	2208.56
**P17066**	P48741;P17066	HSP76_HUMAN	HSPA6				15260.6				7731.2	3684.99
**P17096**	P17096	HMGA1_HUMAN	HMGA1	60226.2	20800.8	19757.7	71381.4	159046	74815.6	18870.8	31407.4	24995.5
**P17174**	P17174	AATC_HUMAN	GOT1	4853.55		4796.29	2329.73	4765.33	7137.2	2044.24		2146.5
**P17252**	P17252	KPCA_HUMAN	PRKCA				4151.83	5968.33	4353.02	5566.65	1852.23	5532
**P17301**	P17301	ITA2_HUMAN	ITGA2	1604.49				3287.64	3231.92	1778.58		1103.99
**P17480**	P17480	UBF1_HUMAN	UBTF	7562.48	23768.1	7885.94	3552.32	12990.2	3476.59	3578.66	6088.71	4333.83
**P17535**	P17535	JUND_HUMAN	JUND									
**P17540**	P17540	KCRS_HUMAN	CKMT2									
**P17544**	P17544	ATF7_HUMAN	ATF7					3687.55	1987.77			
**P17600**	P17600	SYN1_HUMAN	SYN1				9824.35		1715.56	7554.92	4610.29	12104.5
**P17612**	P22694;P17612	KAPCA_HUMAN	PRKACA	6782.65	7828.47	2939.89	1634.15	4048.59	4098.37	3978.21	1231.37	4550.09
**P17655**	P17655	CAN2_HUMAN	CAPN2	11862.6	6620.18	5944.9	10678.2	6016.29	6125.75	6262.57	10825.8	8593.09
**P17661**	P17661	DESM_HUMAN	DES	26404.3	29683.7	13180.3			15002.1	29182.1		
**P17676**	P17676	CEBPB_HUMAN	CEBPB	4162.39			2248.39	2295.9				
**P17677**	P17677	NEUM_HUMAN	GAP43							4554.2	979.014	4757.68
**P17812**	P17812;Q9NRF8	PYRG1_HUMAN	CTPS1	15358.5	16198.1	14330	30656.8	33544	25735	23146.9	21938.6	25269.6
**P17813**	P17813	EGLN_HUMAN	ENG	1110.6		2482.97	2089.61	5142.84	5347.44			
**P17844**	P17844	DDX5_HUMAN	DDX5	50844.4	53322.1	53079.1	41779.6	66789.4	33581	58759	45531.3	56099.5
**P17858**	P08237;P17858	PFKAL_HUMAN	PFKL	18030.5	19663.3	17716.9	10436.4	7524.72	8483.66	13889	11935.4	14188
**P17900**	P17900	SAP3_HUMAN	GM2A			4122.75	1970.05				2602.18	
**P17931**	P17931	LEG3_HUMAN	LGALS3	13315.9	7793.24	13460.5	5433.24	9621.52	14479.4	338398	30821.3	248200
**P17980**	P17980	PRS6A_HUMAN	PSMC3	12243.1	8173.76	13233.2	15460.7	17755	14241.8	10452.7	8441.17	11776.8
**P17987**	P17987	TCPA_HUMAN	TCP1	33684	18575.3	33348.9	47627.5	44625.2	44679	53306.3	58095.3	52878.2
**P18031**	P18031	PTN1_HUMAN	PTPN1				2885.01	4450.14	3081.88	3999.53	1778.89	4317.59
**P18074**	P18074	ERCC2_HUMAN	ERCC2					3848.34	1379.85			
**P18077**	P18077	RL35A_HUMAN	RPL35A	31284.5	44147.9	18859.4	31786.3	93203.9	48778.1	37370.6	33428	40298.5
**P18085**	P18085	ARF4_HUMAN	ARF4	9990.72	9036	14454.8	31273.7	15464.9	7112.24	4287.75	14409.3	3580.35
**P18124**	P18124	RL7_HUMAN	RPL7	37696.4	57103	45161.2	58388.4	78204	51164.3	56958.6	47606.2	51317.3
**P18206**	P18206	VINC_HUMAN	VCL	10411.1	6682.09	9897.85	10634.1	12972	22102.9	11655.9	4751.26	11553
**P18615**	P18615	NELFE_HUMAN	NELFE					1222.05	1704.9			
**P18621**	P18621	RL17_HUMAN	RPL17	5133.9	12071.6	6396.26	6504.46	23513.3	17939.9	6920.41	10146.9	5555.19
**P18669**	P18669	PGAM1_HUMAN	PGAM1	10882.6	3825.39	14264	5782.14	15653.8	40716.3	15553.2	3974.83	18632.3
**P18754**	P18754	RCC1_HUMAN	RCC1		1973.91	1928.03	1156.34	2619.38	3811.29	2060.3		1986.94
**P18858**	P18858	DNLI1_HUMAN	LIG1				1349.45	1662.45	2014.57	1496.79		1458.39
**P18887**	P18887	XRCC1_HUMAN	XRCC1	23577.4	44539.1		29809.6	34590.5	4891.12	4003.28	5630.28	2654.28
**P19012**	P19012	K1C15_HUMAN	KRT15			29540.5			3491.35	6972.71	79614.8	6132.75
**P19013**	P19013;Q86Y46;Q3SY84	K2C4_HUMAN	KRT4	2200.71	730.45	16307.8	5750.94		4059.71	6378.1	49555.8	5571.42
**P19022**	P19022	CADH2_HUMAN	CDH2				3861.56	13949.4	16294.4			
**P19086**	P19086	GNAZ_HUMAN	GNAZ						10186.5	20154.7		22698.1
**P19174**	P19174	PLCG1_HUMAN	PLCG1					4930.72	5691.51	3460.81		1655.37
**P19237**	P19237	TNNI1_HUMAN	TNNI1									
**P19256**	P19256	LFA3_HUMAN	CD58					3711.07	2580.02	6641.07	1889.2	5374.13
**P19338**	P19338	NUCL_HUMAN	NCL	18589.2	14044.4	15846.7	18308.7	19785.5	19570.7	33881.3	17494.5	30080
**P19367**	P19367	HXK1_HUMAN	HK1	2937.02		5289.45	4860.3	7305.72	9252.59	2821.95		2599.23
**P19388**	P19388	RPAB1_HUMAN	POLR2E									
**P19404**	P19404	NDUV2_HUMAN	NDUFV2	3354.63	3500.12	4708.49	3448.29	3884.18	6716.76	10129.7	8149.27	8136.04
**P19525**	P19525	E2AK2_HUMAN	EIF2AK2	5397.55	3598.23	5336.12	3249.64	2449.86	1874.04	2696.78	2610.52	3099.08
**P19532**	P19532	TFE3_HUMAN	TFE3	1991.1			2048.17					
**P19623**	P19623	SPEE_HUMAN	SRM	15359.8	12267.2	17668.4	19718.5		10285.8	16183.1	8983.69	17417.2
**P19634**	P19634	SL9A1_HUMAN	SLC9A1	670.804	786.876	1040.49	296.742	539.095	284.526	1693.56		1051.41
**P19838**	P19838	NFKB1_HUMAN	NFKB1					1168.53	723.098			
**P19957**	P19957	ELAF_HUMAN	PI3			1036.23	627.769				299.464	
**P20020**	P23634;P20020	AT2B1_HUMAN	ATP2B1	6252.69	5880.18	6259.15	4774.91	3105.7	2859.66	2254.61	2276.67	2262.13
**P20042**	P20042	IF2B_HUMAN	EIF2S2	4406.68			4422.02	6501.15	6793.06	4683.28	3903.76	3879.35
**P20073**	P20073	ANXA7_HUMAN	ANXA7	9290.13	3124.49	8306.82	5310.42	4212.62	5889.6	7504.98	5579.88	5874.7
**P20226;Q6SJ96**	Q6SJ96;P20226	TBPL2_HUMAN;TBP_HUMAN	TBP;TBPL2				1416.31					
**P20290**	P20290	BTF3_HUMAN	BTF3		940.669	1153.65	654.341	2870.15	4137.28	1695.04	581.723	1979.2
**P20339**	P20339	RAB5A_HUMAN	RAB5A				3049.65	2316.21	4876.69	4714.38	2656.65	5076.24
**P20340**	P20340	RAB6A_HUMAN	RAB6A	5027.38		6680.31	6026.63	29271.5	3431.75	10771.8	12640.1	8011.41
**P20585**	P20585	MSH3_HUMAN	MSH3	22549.3	41129.5	2574.45	14581.1	11443.7	2432.98	3362.59	11647.4	3203.92
**P20591**	P20591	MX1_HUMAN	MX1									
**P20592**	P20592	MX2_HUMAN	MX2									
**P20618**	P20618	PSB1_HUMAN	PSMB1	5637.08	4132.87	7338.94	3980.93	3150.86	6937.69	5304.18	3530.18	5941.07
**P20700**	P20700	LMNB1_HUMAN	LMNB1	6324.55	5263.5	5862.64		1699.32	2149.32	2002.25		
**P20810**	P20810	ICAL_HUMAN	CAST	3724.99		2556.12	4669.97	5601.62	6440.42	2402.36	3779.03	3025.63
**P20839**	P20839	IMDH1_HUMAN	IMPDH1	4950.32	20506.9	1563.36	5172.47	18229	3132.74	3242.74	1776.98	1194.8
**P20908**	P20908	CO5A1_HUMAN	COL5A1					3323.78				
**P20930**	P20930	FILA_HUMAN	FLG	21430.4	18688.3	24441.3	15156.4	15136.2	14088.2	19044.4	12696.3	21024.9
**P20936**	P20936	RASA1_HUMAN	RASA1					1698.7	2204.1	1275.89		
**P21127;Q9UQ88**	Q9UQ88;P21127	CD11A_HUMAN;CD11B_HUMAN	CDK11A;CDK11B	4903.03	13557.3	3262.94	6644.22	8187.39	3668.73	3928.75	7102.78	6385.13
**P21281**	P21281	VATB2_HUMAN	ATP6V1B2	6344.72	3573.69	8191.14	5378.69	7719.15	11609.6	7030.12	4547.88	7079.34
**P21291**	P21291	CSRP1_HUMAN	CSRP1	9392.05		10960	4012.6	3481.08	14393.8	2590.29		2821.96
**P21333**	P21333	FLNA_HUMAN	FLNA	43933	32637.5	47877.2	47690.9	37198.1	47602.7	32156.4	31491.4	26254.4
**P21359**	P21359	NF1_HUMAN	NF1		3210	1077.69	1320.68	2649.9	2387.78	1040.5	802.588	816.489
**P21399**	P21399	ACOHC_HUMAN	ACO1	7025.1	3911.73	13302.4	3348.1	3391.88	5650.28	2935.47		3153.26
**P21579**	P21579	SYT1_HUMAN	SYT1							7991.49	8568.54	7194.81
**P21589**	P21589	5NTD_HUMAN	NT5E	16212.7	16495.4	15321.7	37940.6	31571.8	42718.9	6065.2	5491.19	4980.49
**P21695**	P21695	GPDA_HUMAN	GPD1					471.322				
**P21796**	P21796	VDAC1_HUMAN	VDAC1	19231.5	25222.9	19156	3134.01	7292.6	6968.66	11175.2	6019.56	10494.4
**P21912**	P21912	SDHB_HUMAN	SDHB				2803.77			5627.91	4612.44	7642.69
**P21926**	P21926	CD9_HUMAN	CD9					4489.07	1309.9	6128.6		6197
**P21953**	P21953	ODBB_HUMAN	BCKDHB	6990.53		10621.3	8833		3088.26	9892.29	13104.2	13498.2
**P21964**	P21964	COMT_HUMAN	COMT			2745.12		2135.84	3538.48	1675.09		1579.98
**P21980**	P21980	TGM2_HUMAN	TGM2	12657.6	11775.3	10430.6	37112.2	31243.5	38419.3	1406.91		886.541
**P22059**	P22059	OSBP1_HUMAN	OSBP					1111.13	1314.5	824.123		935.048
**P22061**	P22061	PIMT_HUMAN	PCMT1				1792.58	5007.18	6474.88	2840.9	865.711	3062.86
**P22087**	P22087	FBRL_HUMAN	FBL	6566.22	11157.3	3986.28	2133.37	8165.21	2821.4	6736.66	5043.12	6521.37
**P22090**	P22090;Q8TD47	RS4Y1_HUMAN	RPS4Y1	10345.8	9601.28	9158.24						
**P22102**	P22102	PUR2_HUMAN	GART	6361.13	3769.77	7155.41	15588.8	19409.7	31177.3	16783.2	16686.9	15046.5
**P22234**	P22234	PUR6_HUMAN	PAICS	6559.57	4357.37	8729.81	10161.8	10972.9	19523.8	29863.2	10255.4	19803.8
**P22307**	P22307	SCP2_HUMAN	SCP2	4739.14			2851.87		4085.62	3652.89		
**P22314**	P22314	UBA1_HUMAN	UBA1	10071.5	9316.19	10244.8	10914.2	9941.49	15443	16360.4	10290.9	12926.9
**P22392**	O60361;P22392	NDKB_HUMAN	NME2	9819.74	5059.7	12245.8	13149.6	5333.56	20147.1	10562.6	8773.4	11083.2
**P22455**	P22455	FGFR4_HUMAN	FGFR4		319094		14213.6	59145.1	58357.6	153475	10865.8	165967
**P22528**	P22528;P35321	SPR1B_HUMAN	SPRR1B	10932.2	14916.9	16137.3	20508		3136.1	8086.84	22495.6	11508.4
**P22532;P35325**	P22532;P35325	SPR2B_HUMAN;SPR2D_HUMAN	SPRR2B;SPRR2D			4379.92	7472.13		4817.23	2303.83	7268.96	5050.73
**P22570**	P22570	ADRO_HUMAN	FDXR				752.191	700.456	919.054	458.848	416.481	392.696
**P22626**	P22626	ROA2_HUMAN	HNRNPA2B1	9730.7	5920.55	11353.2	16166.9	96777.1	35077.7	15822.1	10777.4	14820.8
**P22694**	P22694	KAPCB_HUMAN	PRKACB	944.144	5928.87	733.077	386.495	9401.04	4488.72	2101.15	535.392	3237.71
**P22695**	P22695	QCR2_HUMAN	UQCRC2	3663.46	2893.17	3256.1	3288.74	4789.65	4507.99	9555.55	8064.76	9665.83
**P22735**	P22735	TGM1_HUMAN	TGM1	21661.6	13617.5	25313	21419.4	19994.6	6551.31	11762.1	15517.2	10684.4
**P22830**	P22830	HEMH_HUMAN	FECH	4690.15	6857.57			4644.1	3795.7	2295.78		2270.65
**P23109**	P23109	AMPD1_HUMAN	AMPD1									
**P23193**	Q15560;P23193	TCEA1_HUMAN	TCEA1				3586.27	3354.66	3418.91	1528.79		1636.61
**P23229**	P23229	ITA6_HUMAN	ITGA6				2646.89	3425.91	3409.98	16027.3	5401.09	11433.6
**P23246**	P23246	SFPQ_HUMAN	SFPQ	18328.4	14848.5	15191.7	21595	473317	22560.6	15181.8	15026.1	14354.3
**P23258**	P23258;Q9NRH3	TBG1_HUMAN	TUBG1	6058.92		7467.06	9592.31	4615.99	4236.34	5471.18	16182.9	5706.45
**P23284**	P23284	PPIB_HUMAN	PPIB	10748.9	3803.6	13399.2	6532.8	13367.3	22050.8	8486.44	4686	7025.03
**P23381**	P23381	SYWC_HUMAN	WARS1	12569.4	6151.57	9436.04	4532.46	8058.6	16749.1	5165.32	2361.37	5752.72
**P23396**	P23396	RS3_HUMAN	RPS3	82920.5	90624.3	73963.4	116080	131586	79042.5	91429.9	128895	84921.5
**P23458**	P23458	JAK1_HUMAN	JAK1	8822.73	7095.52	14851.3	2898.82	3214.92	3728.93	4174.71	4484.32	9538.38
**P23470**	P23470	PTPRG_HUMAN	PTPRG					7602.2	7473.83			
**P23490**	P23490	LORI_HUMAN	LORICRIN	6000.67	5765.98	7385.8	4054.32		1216.82	3083.79	4248.08	4579.76
**P23526**	P23526	SAHH_HUMAN	AHCY	16516.9	17717.5	15917.2	22367.8	15198.6	31580.1	46581.8	23102.4	48256.6
**P23527;P33778;Q16778;Q8N257**	P57053;O99879;O5ONW6;O16778;P62807;O60814;P2	H2B1B HUMAN;H2B1O_HUMAN;H2B2E_HU	H2BC17;H2BC21;H2BC26;H2	121262	94016.1	100538	155399	111123	69112.8	33622.8	90688.9	30193.8
**P23528**	Q9Y281;P23528	COF1_HUMAN	CFL1	22622.1	15896.6	20328	28500.4	52214.7	44294.5	17683.6	20192.3	16955.4
**P23588**	P23588	IF4B_HUMAN	EIF4B				4845.91	5197.9	10284.3	6528.19	5111.41	7996.37
**P23743**	P23743	DGKA_HUMAN	DGKA				49779.5					
**P23786**	P23786	CPT2_HUMAN	CPT2						1088.82	973.017		675.592
**P23919**	P23919	KTHY_HUMAN	DTYMK			4375.7	4045.19		3670	2584.36	2555.56	2826.77
**P23921**	P23921	RIR1_HUMAN	RRM1				4497.45	4033.37	6833.25	8119.41	5703.92	5382.77
**P24158**	P24158	PRTN3_HUMAN	PRTN3	5828.15		23888	4366.07		2344.48	3458.11	4434.79	3451.4
**P24278**	P24278	ZBT25_HUMAN	ZBTB25									
**P24534**	P24534	EF1B_HUMAN	EEF1B2	4829.18	3553.16	5241.6	5811.65	8564.21	5583.39	4396.09	5278.29	7546.62
**P24539**	P24539	AT5F1_HUMAN	ATP5PB	7124.49	6507.86	7198.27			1139.56	4061.58		5471.09
**P24666**	P24666	PPAC_HUMAN	ACP1						3287.01			
**P24752**	P24752	THIL_HUMAN	ACAT1	6973.62	7776.85	8001.58	6086.64	4583.15	7254.36	6754.31	6345.84	6173.95
**P24844**	P24844	MYL9_HUMAN	MYL9	8740.4	8023.85	4076.61	7193.3	62157.6	26458.3	7042.46	4127.58	
**P24928**	P24928	RPB1_HUMAN	POLR2A				2943.42	4704.77	2641.5	4925.58	3756.72	4518.04
**P24941**	P24941	CDK2_HUMAN	CDK2				960.029	4450.44	2832.45	2406.62	1380.86	1041.69
**P25205**	P25205	MCM3_HUMAN	MCM3	6411.81	8402.65	6301.12	5188.7	4530.28	4919.26	7805.33	5647.45	6730
**P25311**	P25311	ZA2G_HUMAN	AZGP1	14910.5	13170.8	21012.1	13158.8	23855.3	11224.6	7273.55	13927.5	11283.3
**P25398**	P25398	RS12_HUMAN	RPS12	110045	98132	64786	80097.9	84998.1	69125.1	96155.4	78494.4	75037.2
**P25685**	P25685	DNJB1_HUMAN	DNAJB1	5218.92		10658.7	2750.74	3512.97	4871.4	3076.67	3645.26	3570.63
**P25705**	P25705	ATPA_HUMAN	ATP5F1A	56803.1	45057.6	56079.9	26438.3	19162	15251.1	32274.4	34765.1	30068.5
**P25786**	P25786	PSA1_HUMAN	PSMA1	6516.61	4620.31	7498.33	5971.42	5012.51	10104.2	5396.75	4290.09	6476.07
**P25787**	P25787	PSA2_HUMAN	PSMA2	2953.57	2803.13	3596.13	2518.27	3827.71	6871.61	5198.09	3574.83	5325.96
**P25788**	P25788	PSA3_HUMAN	PSMA3	3567.4		6681.7	5946.04	9149.45	15685.4	6168.94	6321.06	8168.73
**P25789**	P25789	PSA4_HUMAN	PSMA4	6623.95	4809.16	10851	4247.38	4684.51	8368.92	10299.7	3306.63	8708.51
**P26006**	P26006	ITA3_HUMAN	ITGA3				4755.24	7535.87	7044.62			1035.32
**P26038**	P26038	MOES_HUMAN	MSN	50011.9	24763.6	54191.5	34852.7	38703.2	76845.7	48200.8	20713.2	49827.5
**P26196**	P26196	DDX6_HUMAN	DDX6	16327.3	12985.3	15484.7	12010.6	26630.3	27629.7	37801.2	13879.4	31768.8
**P26232**	P26232	CTNA2_HUMAN	CTNNA2					13763.8	7691.21	7831.89		3368.4
**P26358**	P26358	DNMT1_HUMAN	DNMT1					5013.36	3930.06	3022.17		1897.53
**P26368**	P26368	U2AF2_HUMAN	U2AF2	20029.1	24799.7	15241.9	14601.6	83497.4	14500.7	14272.1	16532.1	13453.8
**P26373**	P26373	RL13_HUMAN	RPL13	32836.4	34833	34360.3	41207.9	40588.1	36228.5	40547	29610.3	36535.1
**P26440**	P26440	IVD_HUMAN	IVD			1677.93	3308.5	1261.88	2291.41	3264.97	3514.08	2957.87
**P26447**	P26447	S10A4_HUMAN	S100A4				10052.7	7263.27	7859.06	14781.2	17265.8	16512.1
**P26583**	P26583	HMGB2_HUMAN	HMGB2	7655.78	9180.65	7663.32	5368.03	5782.73	7772.06	5412.62	4764.9	6428.01
**P26599**	P26599;O95758	PTBP1_HUMAN	PTBP1	23693.9	12017.8	13595.4	15240.6	34186.1	17308.6	25895.3	16088.6	26516.7
**P26639**	P26639;A2RTX5	SYTC_HUMAN	TARS1	12070.7	23987	10913.5	13581.2	6292	13321.4	8929.77	6658.55	14117.2
**P26640**	P26640	SYVC_HUMAN	VARS1	7873.82	3936.02	7826.45	11671	12676.5	13258.8	11256	7938.77	10768.6
**P26641**	P26641	EF1G_HUMAN	EEF1G	99127.8	68996.6	72672.3	124272	142482	180407	207293	144472	187789
**P26885**	P26885	FKBP2_HUMAN	FKBP2									
**P27105**	P27105	STOM_HUMAN	STOM	13323.1	11153.7	19118.5	2213.42	1581.11	1999.19	1090.91	1831.17	1111.74
**P27144**	P27144;A0A0S5KW96	KAD4_HUMAN	AK4	6570.85	4146.02	6353.72	10914	10925	13730.3	6803.94	5317.94	6918.47
**P27348**	P27348	1433T_HUMAN	YWHAQ	2013.76		5101.34	2448.47	3421.96	10488.6	5286.61	1067.82	6570.74
**P27361**	P27361;P31152;Q16659;P28482	MK03_HUMAN	MAPK3	5607.88	5738.75	6709.07	3401.81	4848.22	8510.41	4671.4	3489.18	5088.94
**P27448**	P27448	MARK3_HUMAN	MARK3				2101.1			4111.35	2132.79	3761.28
**P27635**	P27635;Q96L21	RL10_HUMAN	RPL10	31448.7	39025.1	34923.4	27545.4	60854.4	51233.6	37303.3	25167.3	41539.3
**P27694**	P27694	RFA1_HUMAN	RPA1	112338	536750	16590.8	55820.7	56745.3	17591.4	13505.7	114422	7695.42
**P27695**	P27695	APEX1_HUMAN	APEX1	3332.51	3705.32	3629.29	2267.83	3137.32	6412.54	3672.91	1899.29	3668.17
**P27708**	P27708	PYR1_HUMAN	CAD	10858.9	10691.4	7253.32	27698.5	20851.8	16755	31678.3	43066.6	28289
**P27797**	P27797	CALR_HUMAN	CALR	9825.34	3790.93	11174.3	5644.51	5259.7	8580.25	4984.91	4809.18	5983.37
**P27816**	P27816	MAP4_HUMAN	MAP4	16345.4	11415.3	12867.9	10644.4	8696.92	11007.9	5878.89	6556.6	6431.67
**P27824**	P27824	CALX_HUMAN	CANX	11900.5	6793.36	8558.36	5851.35	7854.74	8451.12	9393.4	4988.78	9909.05
**P28062**	P28062	PSB8_HUMAN	PSMB8			3286.03			2105.99			2439.66
**P28066**	P28066	PSA5_HUMAN	PSMA5	3449.27	3731.04	4668.8	3026.25	2808.08	5058.85	5748.52	3673.4	6699.9
**P28070**	P28070	PSB4_HUMAN	PSMB4	3219.05		5259.78			181.094			624.788
**P28072**	P28072	PSB6_HUMAN	PSMB6	6528.28		6760.76	5676.34	4789.24	9819.36	5687.35	5253.23	6128.15
**P28074**	P28074	PSB5_HUMAN	PSMB5	3838.77	3040.38	8757.48	3158.24	7078.55	17592.8	7915.07	2506	9191.18
**P28288**	P28288	ABCD3_HUMAN	ABCD3			1641.84	3129.92	3619.45	1026.5	1892.09	2443.89	2330.02
**P28290**	P28290	ITPI2_HUMAN	ITPRID2	6868.2	4755.19	5546.15	4686.39	3929.31	3475.42	10073.4	7475.09	9579.96
**P28300**	P28300	LYOX_HUMAN	LOX	1059.88		934.321						
**P28325**	P28325	CYTD_HUMAN	CST5			845.913						
**P28330**	P28330	ACADL_HUMAN	ACADL									
**P28331**	P28331	NDUS1_HUMAN	NDUFS1	2229.05		2857.75	1825.62	7479.89	5847.92	4500.24	4169.29	4092.78
**P28340**	P28340	DPOD1_HUMAN	POLD1			2543.78	11828.7	12104	9009.34	18892.3	18355.4	15808.3
**P28347**	Q99594;P28347;Q15561	TEAD1_HUMAN	TEAD1				902.185	3690.7	1084.76	810.221		1686.24
**P28482**	P28482	MK01_HUMAN	MAPK1					3157.98	5196.16	1600.65		1984.93
**P28749**	P28749	RBL1_HUMAN	RBL1									493.11
**P28799**	P28799	GRN_HUMAN	GRN	30926.7	34487.5	22177.2				3495.2		
**P28838**	P28838	AMPL_HUMAN	LAP3	7191.09		6547.08	4311.76	3850.43	4509.59	4891	4582.44	3212.99
**P29144**	P29144	TPP2_HUMAN	TPP2				2150.22	6153.72	3894.93	4371.52	3507.72	4619.37
**P29218**	P29218	IMPA1_HUMAN	IMPA1	1813.68		1933.12	2668.13	1382.39	3603.61	2500.93	3258.19	2595.34
**P29317**	P29317	EPHA2_HUMAN	EPHA2				4422.69	5851.02	6054.55	12158.7	5716.32	9260.34
**P29323**	P29323;P54762	EPHB2_HUMAN	EPHB2							5992.24		1690
**P29372**	P29372	3MG_HUMAN	MPG	4872.25	10255.2		6898.55	15975.9	4689.84	2753.39	8258.34	
**P29373**	P29373	RABP2_HUMAN	CRABP2			1224.04	1535.42	805.263	2928.85	698.68	964.452	779.015
**P29401**	P29401	TKT_HUMAN	TKT	28168.9	11664.6	25693	8632.74	11624.5	28500.5	14058.2	6255.42	15720.8
**P29508**	P29508	SPB3_HUMAN	SERPINB3	24908.8	21109.7	32520.3	20252.2	4822.8	13195.9	14197.9	21377.8	26756.6
**P29558**	P29558	RBMS1_HUMAN	RBMS1	62317.7	35704.8	5246.36	44293.7	12811.5	1885.98		29484.6	
**P29692**	P29692	EF1D_HUMAN	EEF1D	10304.5	8285.74	6459.28	16228.1	4026.38	8544.1	17931.6	8907.84	11370.2
**P29728**	P29728	OAS2_HUMAN	OAS2		3276.63	1713.06	1866.93	1149.09	1034.85			
**P29966**	P29966	MARCS_HUMAN	MARCKS	8981.75	3910.52	6291.89	13909.7	21948.2	24898.1	30252.1	13352.2	25985.4
**P29992**	P29992	GNA11_HUMAN	GNA11	2208.71		1912.77	12944.9	25449.9	25630.2	49861.7	11344.3	43218.9
**P30038**	P30038	AL4A1_HUMAN	ALDH4A1				2550.87			2392.13	4752.41	2088.71
**P30040**	P30040	ERP29_HUMAN	ERP29	12034.8	3496.32	9841.33	9367.64	9275.14	22632.4	4002.42		6358.88
**P30041**	P30041	PRDX6_HUMAN	PRDX6	22227.9	14809.7	32123.1	9762.96	11808.2	31489	13359.4	6124.99	14221.4
**P30043**	P30043	BLVRB_HUMAN	BLVRB	7446.49		6143.58			2977.7			
**P30044**	P30044	PRDX5_HUMAN	PRDX5	8122.86		6159.46	6666.08	3556.15	13696.2	2404.17		4406.94
**P30046**	A6NHG4;P30046	DOPD_HUMAN	DDT	4878.88		4893.96	2681.87	4167.14	10609.5	2849.65	2611.31	2051.15
**P30048**	P30048	PRDX3_HUMAN	PRDX3	19399.7	9529.79	23658.4	11635.3	7885.12	28473.5	25053.7	22375.3	20677.4
**P30050**	P30050	RL12_HUMAN	RPL12	27654.6	23300.3	22370.7	37595.6	20802	18870.5	22563.3	28680.9	23409.4
**P30084**	P30084	ECHM_HUMAN	ECHS1	6633.64	2002.59	9894.74	2639.17	3187.94	12276.3	8133.69	2300.67	9015.7
**P30085**	P30085	KCY_HUMAN	CMPK1			4423.76	3137.76	2635.95	3107.84	1915.75	2755.61	2977.45
**P30086**	P30086	PEBP1_HUMAN	PEBP1	3273.09		7890.11	3336.75		2779.18	2109.09	3093.08	2928.61
**P30101**	P30101	PDIA3_HUMAN	PDIA3	26211.7	7351.11	25411.6	16016.8	14927.2	34088.1	21891.8	9309.88	20377.3
**P30153**	P30153	2AAA_HUMAN	PPP2R1A	7451.43	8226.9	8545.85	11053.2	8032.19	14979	11726.9	9012.16	10878.2
**P30154**	P30154	2AAB_HUMAN	PPP2R1B	1273.28		839.977	3011.98	4568.18	5463.05	4515.9	1552.62	1662.76
**P30405**	P30405	PPIF_HUMAN	PPIF				4966.54		7372.4	3259.78		2975.56
**P30419**	P30419	NMT1_HUMAN	NMT1			986.972		2445.17	3082.71	1324.24	1337.61	1449.01
**P30519**	P30519	HMOX2_HUMAN	HMOX2					2312.18	2944.41	3033.3		1358.78

P30520	P30520	PURA2_HUMAN	ADSS2				4252.14		1726.81	3299.38		2527.67
P30530	P30530	UFO_HUMAN	AXL		1405.59	1801.01		1447.05	2145.5			
P30566	P30566	PUR8_HUMAN	ADSL					6224.35	11748	2649.65		1676.74
P30740	P30740	ILEU_HUMAN	SERPINB1	7618.81	5375.48	8589.91	5786.22	3958.2	5113.11	13477	9814.79	14916.5
P30837	P30837	AL1B1_HUMAN	ALDH1B1	7401.75	5489.12	7571.49	10856.6	12915.1	6594.3	11715.6	13792	12962
P30838	P30838	AL3A1_HUMAN	ALDH3A1		24450.2					1368.35		
P30876	P30876	RPB2_HUMAN	POLR2B	6301.61	6623.33	5913.48	8283.42	11298	11058.5	13628.7	10568.5	12878.8
P31025	P31025	LCN1_HUMAN	LCN1	33368.3		31319.1	25278.5		4546.81	6410.3	98664.8	5488.18
P31040	P31040	SDHA_HUMAN	SDHA			3727.95			1319.69	2810.34	2163.66	3503.31
P31150	P31150	GDIA_HUMAN	GDI1			1925.18	1686.51		795.825			561.3
P31151	P31151;Q86SG5	S10A7_HUMAN	S100A7	35884.2	31794.5	57931.3	34365.8	15572.2	20424.1	14924.7	46325.5	23886.1
P31153	P31153	METK2_HUMAN	MAT2A	9834.3	9644.68	8608.83	9347.17	6744.73	13370.3	28664.7	23383.3	29162.7
P31323	P13861;P31323	KAP3_HUMAN	PRKAR2B	7832.81	12944.7	5878.97	3143.2	2511.55	3068.13	2114	1067.8	1855.99
P31327	P31327	CPSM_HUMAN	CPS1									
P31350	P31350	RIR2_HUMAN	RRM2					1256.26	2119.96	1796.87	3777.93	2508.5
P31689	P31689	DNJA1_HUMAN	DNAJA1	1785.42	1105.93	6434.32	18204.2	17642.2	18045.4	27955.6	35422.3	26592.1
P31930	P31930	QCR1_HUMAN	UQCRC1	4696.55	4060.47	4332.45	2035.95	3180.09	3137.06	9383.1	5161.24	9350.27
P31937	P31937	3HIDH_HUMAN	HIBADH				1068.38	4124.73	9049.1	1180.32		1059.62
P31939	P31939	PUR9_HUMAN	ATIC	3376.11		5492.2	5918.77	7143.35	14019.8	5349.78	2357.3	5840.15
P31942	P31942	HNRH3_HUMAN	HNRNPH3					34513.7	7769.78	2606.15		6942.52
P31943	P31943	HNRH1_HUMAN	HNRNPH1	35072.8	38612	37958.4	32212.4	82975.7	34858.3	34170.3	23934.8	36214.1
P31944	P31944	CASPE_HUMAN	CASP14	18282.4	13766.4	22697.4	12292.7	12767.8	5891.15	6646.26	10946.6	9757.36
P31946	P31946	1433B_HUMAN	YWHAB			13198.7	5361.87	4978	11048.8	6559.11	3634.9	5933.92
P31947	P31947	1433S_HUMAN	SFN	6725.88	4383.89	5282.95	3538.05	2587.92	6620.09	4698.8	2492.72	5095.51
P31948	P31948	STIP1_HUMAN	STIP1	9053.8	3157.57	9153.4	17504.8	21073	39309.2	18992.2	7518.16	18299.4
P31949	P31949	S10AB_HUMAN	S100A11	7128.61			8456.78	5949.04	9587.55	6600.2	7485.55	4630.24
P32004	P32004	L1CAM_HUMAN	L1CAM					1894.24	1031.3	1773.93		1432.12
P32119	P32119	PRDX2_HUMAN	PRDX2	12738.4	9016.18	16750.2	7635.6	7089.77	8353.54	18992.7	8373.54	16555.2
P32189;Q14409	Q14410;P32189;Q14409	GLPK3_HUMAN;GLPK_HUMAN	GK;GK3	14416.8	12519.3	7902.1						
P32320	P32320	CDD_HUMAN	CDA	4312.34	3102.43	5635.98	3229.08		2434.72	2305.23		2936.58
P32322	P32322	P5CR1_HUMAN	PYCR1	2855.94	4039.22	3931.39	3804.24	4556.4	5142.96	18620.8	12857.6	15637.2
P32455	P32455	GBP1_HUMAN	GBP1				1499.59	1822.33	1667.97	1533.8	1167.26	5693.56
P32929	P32929	CGL_HUMAN	CTH					1011.47	1463.91			879.618
P32969	P32969	RL9_HUMAN	RPL9P9	16331	12248.5	19346.4	15528.4	29555.2	19848.5	15296.7	16090.6	14971.1
P32970	P32970	CD70_HUMAN	CD70				3569.96	6635.94	6977.83			
P33121	P33121	ACSL1_HUMAN	ACSL1	3469.34	4002.41	5615.9				3937.76	3110.66	4058.07
P33176	P33176	KINH_HUMAN	KIF5B	10808.9	5524.94	9877.29	11052.3	9683.35	9016.5	7308.52	6685.45	6192.49
P33316	P33316	DUT_HUMAN	DUT			9159.15	6226.09	5008.37	10316.7	9032.09	6858.49	7560.99
P33527	P33527	MRP1_HUMAN	ABCC1		1859.57				614.019	1698.8	594.481	610.663
P33897	P33897	ABCD1_HUMAN	ABCD1							2633.98		1762.21
P33981	P33981	TTK_HUMAN	TTK				775.77	1384.19		949.697	1436.09	1081.76
P33991	P33991	MCM4_HUMAN	MCM4	7179.63	9351.54	10993.3	12268.4	17869.7	14083.3	20961.8	15358	18922.5
P33992	P33992	MCM5_HUMAN	MCM5	15379.8	15627.3	13321.2	6675.02	4692.32	7192.3	9765	6125.75	9287.51
P33993	P33993	MCM7_HUMAN	MCM7	18177.4	21006.8	14567.7	15426.4	23236.3	18115.3	22743.2	18761.7	24218.7
P34897	P34897	GLYM_HUMAN	SHMT2	9041.25	7434.3		13680.3	14004.1	16116.1	14033.3	13845.5	11128.5
P34932	P34932	HSP74_HUMAN	HSPA4	7709.31	5207.55	7555.27	9792.98	13054.3	16519.1	8736.13	4885.63	8771.32
P35030	P35030	TRY3_HUMAN	PRSS3	3679.55	860.946	2958.46	5174.83	1052.67	1115.03	2602.09	8053.92	1682.35
P35080	P35080	PROF2_HUMAN	PFN2	9666.93		6086.71	6306.48	1739.23	958.741	1713.42	5063.99	
P35221	P35221	CTNA1_HUMAN	CTNNA1		2546.49	3936.08	6182.5	11914.1	9494.13	14828.4	5598.91	12198.6
P35222	P35222	CTNB1_HUMAN	CTNNB1				3624.1	6143.65	3026.03	8319.53	4191.69	7696.97
P35232	P35232	PHB1_HUMAN	PHB1	5641.57		6968.86	5881.43	7968.21	4229.23	9191.85	7863.36	11078.9
P35237	P35237	SPB6_HUMAN	SERPINB6	9980.42	3853.53	12716	10911	6885.21	13898.5	15877.8	24783.7	19794
P35241	P35241	RADI_HUMAN	RDX	6846.81		4688.61	3839.72	6295.98	9331.15	3759.87		2771.77
P35244	P35244	RFA3_HUMAN	RPA3	85883.7	335084	5822.2	26005.1	214681	21010.4	14653.4	81210.3	2895.37
P35249	P35249	RFC4_HUMAN	RFC4	4329.49	5494.51	4097.48	7756.42	9087.54	6788.17	16372.8	13338.6	18088
P35250	P35250	RFC2_HUMAN	RFC2				4568.31	4955.75	2920.56	6868.75	7908.55	8295.77
P35251	P35251	RFC1_HUMAN	RFC1		1984.73			3197.05	1336.94	1485.31	1230.61	1816.41
P35268	P35268	RL22_HUMAN	RPL22	33297.3	22531.1	25015.3	27641.2	63677.5	29617.1	24597.7	42928.3	26298.8
P35269	P35269	T2FA_HUMAN	GTF2F1				1064.44	1192.17	704.227	486.976	1112.18	617.883
P35270	P35270	SPRE_HUMAN	SPR						2572.87	1139.59		1668.02
P35520	P35520	CBS_HUMAN	CBS				3081.09	5203.13	7241.47	1737.11		
P35527	P35527	K1C9_HUMAN	KRT9	#########	#########	2.19672E+06	1.35603E+06	377223	504654	488492	1.21211E+06	850432
P35568	P35568	IRS1_HUMAN	IRS1	1583.7				1851.61				
P35573	P35573	GDE_HUMAN	AGL				954.676	13032.2	7400.67	1199.3	1613.37	2848.89
P35579	P35579	MYH9_HUMAN	MYH9	117151	139364	80665.3	404898	733214	384457	222196	192565	157708
P35580	P35580;Q7Z406	MYH10_HUMAN	MYH10	28393.1	31956.6	20729.8	115533	207347	106216	141080	117382	104737
P35606	P35606	COPB2_HUMAN	COPB2	7087.58	6853.68	9283.15	12005.5	15851.4	14856.2	14073.4	12435	12158.9
P35609	P35609	ACTN2_HUMAN	ACTN2									
P35610	P35610	SOAT1_HUMAN	SOAT1		1395.47			2965.12	1895.99	773.26		989.47
P35611	P35611	ADDA_HUMAN	ADD1				2270.41	1910.51	1550.15	1397.53	1822.15	2071.15
P35613	P35613	BASI_HUMAN	BSG	16056.6	6224.84	12270.4	12595.2	7612.1	8523.6	60236.6	39587.8	35084.4
P35637	P35637	FUS_HUMAN	FUS	6857.2	7856.92	6834.84	5159.55	30497.1	14241	4956.26	6099.94	4889.2
P35658	P35658	NU214_HUMAN	NUP214				2209.6	1690.87	1608.76	1408.77	1955.08	1866.44
P35659	P35659	DEK_HUMAN	DEK	9092.58	9697.5	8510.08	6258.61	15371	5002.85	4565.42	6925.34	6117.32
P35754	P35754	GLRX1_HUMAN	GLRX	9736.48	4827.97	9157.14	6119.33	7824.03	8843.41	6563.17	4216.83	7729.5
P35869	P35869	AHR_HUMAN	AHR									
P35900	P35900	K1C20_HUMAN	KRT20				1486.57			772.959		523.862
P35908	P35908	K22E_HUMAN	KRT2	727684	667375	1.36308E+06	717011	226320	402435	559016	656337	803706
P35998	P35998	PRS7_HUMAN	PSMC2	19721.4	17559	20633.7	20607.1	20580	19815	16832	13275.2	15165.2
P36404	P36404	ARL2_HUMAN	ARL 2.00	3506.07			5217.15		2805.68			1467.68
P36507	Q02750;P36507	MP2K2_HUMAN	MAP2K2			9835.17	8790.07	5635.07	7752.43	9663.38	8709.84	9077.19
P36542	P36542	ATPG_HUMAN	ATP5F1C	20628.9	28653.8	24344.6	6873.44	9040.04	9567.48	24364	11189.4	21378.5
P36551	P36551	HEM6_HUMAN	CPOX	3723.63		3590.89	3735.29	4741.5	9514.74	2504.07		3347.64
P36578	P36578	RL4_HUMAN	RPL4	88321.4	88190.2	71958.3	137222	145789	73153.8	96435.9	91184.5	91898.9
P36639	P36639	8ODP_HUMAN	NUDT1				631.412	393.689	1163.79	532.066		402.019
P36776	P36776	LONM_HUMAN	LONP1	3672.13		4996.97	4247.93	4844.39	5957.98	4181.29	2434.26	5213.41
P36871	P36871	PGM1_HUMAN	PGM1			1373.45			2305.69	794.259		
P36915	P36915	GNL1_HUMAN	GNL1				1701.43	1756.94	3642.33	1082.54		
P36957	P36957	ODO2_HUMAN	DLST	14751.4	11298.6	22608.3	31162.2	4182.07	16156	13851.2	13774.6	18841.9
P37059	P37059	DHB2_HUMAN	HSD17B2	6281.56	6225.69	6184.9						
P37108	P37108	SRP14_HUMAN	SRP14	4573.91		7788.18	6110.83	10870.2	11390.1	8658.55	7400.19	5694.37
P37235	P37235	HPCL1_HUMAN	HPCAL1				1709.9	3978.02	5763.15	11119.7	2617.69	14783.1
P37268	P37268	FDFT_HUMAN	FDFT1				8894.13	3735.73	3626.37	2675.42	8455.93	3298.38
P37802	P37802	TAGL2_HUMAN	TAGLN2	20770.1	18426.4	19151.8	19394.9	7390.73	25416.8	8853.47	11685.6	9895.28
P37837	P37837	TALDO_HUMAN	TALDO1	14353.2		16370.1	5127.23	3692.23	8755.69	7753.51	3401.14	7327.11
P38117	P38117	ETFB_HUMAN	ETFB	3611.35	1644.1	3388.77	2229.63	3001.97	5187.56	5548.65	2491.74	4487.96
P38159	P38159	RBMX_HUMAN	RBMX	9310.26	4091.04	11545.2	7137.74	23698.5	10580	10031.2	7358.76	10808.3
P38606	P38606	VATA_HUMAN	ATP6V1A	6035.21	4467.5	5174.22	6585.79	5827.44	6156.6	3935.61	6119.07	5061.21
P38646	P38646	GRP75_HUMAN	HSPA9	110938	96596.4	83370.3	75698.9	100449	116234	74380.9	48289.6	73377.1
P38919	P38919	IF4A3_HUMAN	EIF4A3			13109.1	5838.06	6004.93	6039.25	7405.9	4063.26	7159.11
P39019	P39019	RS19_HUMAN	RPS19	118884	154498	114919	73561.5	155808	95765.1	104240	86957.8	85739.4
P39023	P39023	RL3_HUMAN	RPL3	62686.9	78827.4	48269.6	97938.5	152433	90895.1	83380.3	80437.9	71895.6
P39060	P39060	COIA1_HUMAN	COL18A1	14074.8	9689.03	12687.1			1888.33	6221.54	3558.6	7197.42
P39656	P39656	OST48_HUMAN	DDOST	22444.1	22011.7	20153.8	15750.3	16435	12428.2	36179.7	26309.5	23738.9
P39687	P39687	AN32A_HUMAN	ANP32A	5268.62		5629.41	4190.08	4208.48	10222	5282.3	5391.88	5048.8
P39748	P39748	FEN1_HUMAN	FEN1	5977.06			5880.18	9339.04	17093.9	9444.62	4442.98	6987.22
P39880	P39880	CUX1_HUMAN	CUX1	1999.45	8522.5			4135.48	2381.99	6634.8	4615.72	
P40121	P40121	CAPG_HUMAN	CAPG			2191.96	5345.35	2168.12	6527.99	1874.34		2838.9
P40123	P40123	CAP2_HUMAN	CAP2	5945.25	5632.61		12237.1	3214.66	8160.59	3241.38	15809.4	3771.87
P40199	P40199	CEAM6_HUMAN	CEACAM6							5462.87	2119.72	4738.4
P40227	P40227	TCPZ_HUMAN	CCT6A	15282.1	8485.02	17395.4	17728.1	19654.5	27061.7	36646.3	16226.5	37782.9
P40425	P40425	PBX2_HUMAN	PBX2					6114.94	3305.89	1426.39		1014.78
P40429	Q6NVV1;P40429	RL13A_HUMAN	RPL13A	11452.7	13724.3	12495	13091.9	17016.5	16857.4	13233.6	12227.4	13627.6
P40616	P40616	ARL1_HUMAN	ARL 1.00	3744.89		9722.17	10316.5		1748.85	3253.23	8794	5773.55
P40692	P40692	MLH1_HUMAN	MLH1				900.866	845.026	919.552	942.142	1344.36	910.099
P40763	P40763	STAT3_HUMAN	STAT3	3268.61	3689.96	3871.87	5095.83	4484.85	4478.52	2620.6	1181.35	2379.3
P40818	P40818	UBP8_HUMAN	USP8	1593.21	2265.46	1420.81	4153.24			1179.32		1251.54
P40925	P40925	MDHC_HUMAN	MDH1	10925.8	14261.8	13492.6	12872.4	4180.96	19046.4	9371.37	6767.76	9888.93
P40926	P40926	MDHM_HUMAN	MDH2	16938.5	7228.08	16000.5	12855.4	8245.42	17088.8	12487	14828.2	11476.9
P40937	P40937	RFC5_HUMAN	RFC5	3762.03	3897.59	4366.18	8662.88	5328.67	4852.07	15217.5	18337.6	18812.7
P40938	P40938	RFC3_HUMAN	RFC3	3423.79	4955.41	2276.5	5515.95	5554.44	4961.69	9026.47	7378.24	9575.21
P40939	P40939	ECHA_HUMAN	HADHA	7790.18	5800.15	6636.04	6666.98	5736.06	5729.83	8861.77	7215.35	8148.15
P41091	Q2VIR3;P41091	IF2G_HUMAN	EIF2S3	4292.77	3764.89	3778.77	9799.13	13327.8	15820.6	10926	9260.58	10281.6
P41162	P41162	ETV3_HUMAN	ETV3				3711.65	1917.1	2401.8	5153.34	4426.54	3570.94
P41208	Q12798;P41208	CETN2_HUMAN	CETN2		12311			5114.81				
P41227	P41227	NAA10_HUMAN	NAA10					9394.65	6259.33	9609.18	5218.05	8271.92
P41240	P41240	CSK_HUMAN	CSK		1743.6	1458.46		10510.6	11421.9	8279.99	2166.53	9538.99
P41247	P41247	PLPL4_HUMAN	PNPLA4				201.472	999.353	478.625	924.27		1470.09
P41250	P41250	GARS_HUMAN	GARS1	7411.66	4208.72	6577.57	8830.38	8988.24	14414.1	5321.5	3058.45	5699.76
P41252	P41252	SYIC_HUMAN	IARS1	15380.4	17826.2	19030.9	34562.2	21220.2	22999.5	17607.9	22091	19004.9
P41743	P41743	KPCI_HUMAN	PRKCI				2629.8	5441.87	6611.77	3180.03	2951.6	2942.48
P42025	P42025	ACTY_HUMAN	ACTR1B			1737.91		1361.12	2187.7	632.544		
P42126	P42126	ECI1_HUMAN	ECI1	3349.17		4451.75	2870.74		5621.18	4649.76	3908.97	5221.34
P42166	P42166	LAP2A_HUMAN	TMPO	19183.8	16022.3	15666.9	31020.4	32632.4	20788.5	10629.9	18870.4	13666.1
P42224	P42224	STAT1_HUMAN	STAT1	32279.6	37419.1	38598.1	13290.3	9674.36	12641.9	9560.4	9535.98	9858.8
P42226	P42226	STAT6_HUMAN	STAT6	2958.04	3756.29	3222.91	4526.39	2944.86	4265.14	2834.92	1847.57	2655.15
P42229	P42229	STA5A_HUMAN	STAT5A									
P42285	P42285	MTREX_HUMAN	MTREX				1132.4	6769.6	3834.74	3036.87	2624.22	3317.38
P42330	P42330	AK1C3_HUMAN	AKR1C3	3914.77		3216.82				2024.97		1733.7
P42345	P42345	MTOR_HUMAN	MTOR	3545.54	2757.66	3274.3	4291.07	4295.57	4393.66	3265.35	7458.98	4744.38
P42356	P42356	PI4KA_HUMAN	PI4KA			1644.54	2923.72	5967.17	5627.39	5645.12	2273.27	5164.35
P42357	P42357	HUTH_HUMAN	HAL			5394.42	5620.35		2865.89	1455.5	5683.49	2347.3
P42566	P42566	EPS15_HUMAN	EPS15		457.092	1697.02	2279.44	4999.29	4672.45	2187.62		1532.6
P42677	P42677;Q71UM5	RS27_HUMAN	RPS27	25966.1	31585.1	27126.6	35805.4	23965.4	25637.5	39703.7	45205.2	37467.5
P42694	P42694	HELZ_HUMAN	HELZ		770.123			5367.51	2223.53	1359.5	495.453	
P42695	P42695	CNDD3_HUMAN	NCAPD3				587.989			866.704	1733.32	1442.75
P42704	P42704	LPPRC_HUMAN	LRPPRC	8098.65	11136.4	10497.1	13331.3	10477.1	12016.3	18205.1	16127.2	18540
P42765	P42765	THIM_HUMAN	ACAA2	4489.08		4617.37	1885.57	1779.74	4067.89	2645.03	2172.63	3046.51
P42766	P42766	RL35_HUMAN	RPL35	19687.5	19301.4	11735.3	20816.2	49401.3	18690.6	16789.8	19430.5	13819
P42771	P42771	CDN2A_HUMAN	CDKN2A	4337.01	2353.39	4372.15			802.588			
P42785	P42785	PCP_HUMAN	PRCP				720.911	3237.65	5037.24	4402.4	1053.89	2810.72
P42892	P42892	ECE1_HUMAN	ECE1	3304.54		1779.66	1843.71	1781.12	2069.57	895.903	5208.48	1746.39
P43034	P43034	LIS1_HUMAN	PAFAH1B1	2478.87		2705.33	1788.3	2363.19	4400.69	3932.04	2585.95	3481.97
P43121	P43121	MUC18_HUMAN	MCAM				15989.5	34543	33033.9	6327.06	4393.95	7609.73
P43243	P43243	MATR3_HUMAN	MATR3	6534.96	8717.22	6949.09	5247.78	6544.73	2706.11	4523.93	5347.27	4779.3
P43246	P43246	MSH2_HUMAN	MSH2	19622.5	28278.7	6623.34	18774.1	16536.5	8445.61	9695.02	18775.5	10591.6
P43250	P43250	GRK6_HUMAN	GRK6							2226.36		2962.48
P43304	P43304	GPDM_HUMAN	GPD2	5708.45	3491.55	5856.71	3007.11	3015.03	2351.02	2944.62	2481.87	2997.44
P43307	P43307	SSRA_HUMAN	SSR1					2267.68	2448.37	1158.61	1313.47	3656.75
P43362	P43362	MAGA9_HUMAN	MAGEA9B				2600.15	2011.33	1952.08			
P43405	P43405	KSYK_HUMAN	SYK							8449.8	6961.28	8427.59
P43487	P43487	RANG_HUMAN	RANBP1				3157.41	1864.3	3855.86	2446.24		2682.78
P43490	P43490	NAMPT_HUMAN	NAMPT	18941.1	11983	22578.2	9858.76	11552.6	31128.6	7146.17	4297.72	8495.22
P43686	P43686	PRS6B_HUMAN	PSMC4	19698.4	16756.1	20811.1	22065.1	22723	24508.6	22714	20074.4	22519
P43699	P52952;Q9H2Z4;P43699	NKX21_HUMAN	NKX2-1			4345.05			1498.58			
P45880	P45880	VDAC2_HUMAN	VDAC2	21174.1	16794	19616.9	3988.55	4260.47	4397.77	8101.52	7861.11	7993.29
P45954	P45954	ACDSB_HUMAN	ACADSB	2968.95		3328.8	1294.8	1039.4	1525.55	3150.45	5118.95	3776.71
P45974	P45974	UBP5_HUMAN	USP5	3040.02		7112.44	6524.11	5658.73	10835.9	6851.53	3295.27	6566.75
P46013	P46013	KI67_HUMAN	MKI67							2497.11		2369.84
P46020	P46020	KPB1_HUMAN	PHKA1	15786.6				5132.84	3773.41	3525.21		3690.11
P46060	P46060	RAGP1_HUMAN	RANGAP1	4201.47		5393.01	6733.39	4163.57	5026.23	3970.93	4683.54	3801.37
P46063	P46063	RECQ1_HUMAN	RECQL	15419.8	24096.4	3289.58	12227.8	13461.6	7628.52	3971.3	18113.8	4779.09
P46087	P46087	NOP2_HUMAN	NOP2	5631.51	4146.86	4349.22	1556.82		1652.9	3801.12	5324	5049.59
P46109	P46109	CRKL_HUMAN	CRKL					2078.55	3343			
P46379	P46379	BAG6_HUMAN	BAG6	6784.69	6805.28	8419.38	6808.06	8052.55	10912.9	7264.55	4614.76	8242.7
P46439	P46439	GSTM5_HUMAN	GSTM5				3677.81					
P46459	P46459	NSF_HUMAN	NSF	18635.9	16041.2	17606.5	22003.8	12894	12203.4	12795.7	17316.9	13928.8
P46734	P46734	MP2K3_HUMAN	MAP2K3			1989.8	2785.35	3162.9	2084.45	1042.9	2726.84	2051.33
P46776	P46776	RL27A_HUMAN	RPL27A	58575.1	53500.8	58557	83437.6	80556.5	45411.8	44165.1	112346	54588.5
P46777	P46777	RL5_HUMAN	RPL5	22809.3	19501	29459.6	41072.1	66531.6	92338.8	52335.2	30346.9	45398.4
P46778	P46778	RL21_HUMAN	RPL21	9488.2	10048.6	11088.3	13832.4	18371.2	17921.2	15120.6	11146.7	17739
P46779	P46779	RL28_HUMAN	RPL28	25279	18689.6	24821.8	37564.9	22867.1	20085.8	21135	36442.9	25218
P46781	P46781	RS9_HUMAN	RPS9	73581.6	78801.9	69792.1	84839.4	127985	95691.1	87952.9	64406.4	97564.5
P46782	P46782	RS5_HUMAN	RPS5	23852.8	35580.1	30451.5	26079.1	48007.6	40690.4	34552.9	28171.4	34188.8
P46783	Q9NQ39;P46783	RS10_HUMAN	RPS10	36276.9	38677.6	36766.8	42789.3	122975	67059.6	70127	40853.6	65507.9
P46821	P46821	MAP1B_HUMAN	MAP1B		13735.2	3725.65	11180.5	10766.8	12863.9			
P46926	P46926;Q8TDQ7	GNPI1_HUMAN	GNPDA1			3025.59			3216.57	1296.57		
P46934	P46934	NEDD4_HUMAN	NEDD4	945.653	895.375	1363.97	1222.29	1471.31	2572.38	807.122		665.208
P46937	P46937	YAP1_HUMAN	YAP1					240.877	621.761			
P46940	P46940	IQGA1_HUMAN	IQGAP1	13975.2	12708.7	12001.6	19673	13910.9	13681.1	8699.54	7685.92	9025.78
P46977	P46977	STT3A_HUMAN	STT3A	6834.48	9717.11	7020.21	6362.09	6345.86	6431.72	6030.46	5077.03	6535.46
P47755	P47755	CAZA2_HUMAN	CAPZA2	4668.27	4651.98	3274.52	9396.3	26417.4	20367.9	8054.71	2167.84	9937.57
P47756	P47756	CAPZB_HUMAN	CAPZB	17186.5	13701.9	23323	27265.1	57405.7	47097.9	39251	17817	31416
P47813	P47813	IF1AX_HUMAN	EIF1AX				1559.84	1560.56	2658.79	1519.55	2379.89	1407.23
P47895	P47895	AL1A3_HUMAN	ALDH1A3		5774.78	5806.27		4529.44		6337.48	7156.33	5301.64
P47897	P47897	SYQ_HUMAN	QARS1	7536.04	6294.77	7830.28	12342.7	7323.16	8958.08	4615.61	5170.85	6029.04
P47914	P47914	RL29_HUMAN	RPL29	10213.6	6674.52	5785.59	13059.1	8070.98	4149.56	5077.44	14949.8	4377.49
P47929	P47929	LEG7_HUMAN	LGALS7B	3268.19		5014.86	2944.94	3499.2	3602.72	2736.26	3858.97	2564.67
P47974	P47974	TISD_HUMAN	ZFP36L2	7246.77	7999.42	6735.61	3415.63	4995.46	3211.27	8322.51	5536.24	10882.6
P48047	P48047	ATPO_HUMAN	ATP5PO	9097.92	6873.38	7306.15	3711.25		2671.81	5080.47	4168.67	3609.47
P48147	P48147	PPCE_HUMAN	PREP				2409.24	4520.1	6244.35	2181.99		3165.55
P48163	P48163	MAOX_HUMAN	ME1									
P48444	P48444	COPD_HUMAN	ARCN1	10054.4	8345.38	9565.03	17239.5	20621.2	23190.8	15563.3	14372.5	12867.6
P48506	P48506	GSH1_HUMAN	GCLC						3315.91	2184.99		1438.36
P48507	P48507	GSH0_HUMAN	GCLM		1152.27	2080.39		836.267	2659.83	833.914		835.483
P48556	P48556	PSMD8_HUMAN	PSMD8	7506.55	5640.27	6730.47	8501.11	4206.26	6537.12	5734.36	7392.45	8328.61
P48594	P48594	SPB4_HUMAN	SERPINB4	3425.97		5932.74	2970.31		2526.9	1734.34	5193.51	4754.94
P48634	P48634	PRC2A_HUMAN	PRRC2A				1841.72	2684.48	1687.38	886.101		688.301
P48637	P48637	GSHB_HUMAN	GSS			1647.58	1484.14	1638.43	2843.23	1790.1	4750.13	2593.19
P48643	P48643	TCPE_HUMAN	CCT5	10232.7	9280.62	11184.5	14517.8	21140.6	29672.6	25121.6	10771.4	28695.8
P48651	P48651	PTSS1_HUMAN	PTDSS1							1494.6	2470.93	2747.53
P48668	P48668	K2C6C_HUMAN	KRT6C			8744.69					8904.51	5378.1
P48681	P48681	NEST_HUMAN	NES	26967.1	31086.8	23850.8	5603.3	11883.1	9773.42	84014	11780.1	83471.9
P48723	P48723	HSP13_HUMAN	HSPA13			574.142	1249.05	1168.38	2213.44	656.331		
P48729	P48729;Q8N752	KC1A_HUMAN	CSNK1A1	3739.95	5976.04	7092.3	3858.69	18608.1	14916.8	25058.9	3431.75	27282.8
P48735	P48735	IDHP_HUMAN	IDH2	6658.18	8677.16	9391.19	5589.35	4894.99	6221.03	13749.3	16352.7	14938.3
P48739	P48739	PIPNB_HUMAN	PITPNB			3428.37	2363.33	2086.49	6761.55	2602.26	1241.02	2394.78
P48960	P48960	AGRE5_HUMAN	ADGRE5		7650.94	4942.2	7237.11	4885.89	5771.75	7083.94	6403.23	5460.05
P49005	P49005	DPOD2_HUMAN	POLD2		1878.1		1377.92	4558.99	3435.74	4498.47	2535.92	4695.84
P49006	P49006	MRP_HUMAN	MARCKSL1				12818.9	6784.4	10776.6	137871	19393.9	100248
P49023	P49023	PAXI_HUMAN	PXN	4017.06		2864.46	2915.28		1306.76	1694.2	2199.13	1760.07
P49184	P49184	DNSL1_HUMAN	DNASE1L1				1571.18			5301.56	1185.97	4881.14
P49189	P49189	AL9A1_HUMAN	ALDH9A1	10946.6		11491.7	6866.49	5580.42	8869.21	6121.96	11366.9	6072.49
P49207	P49207	RL34_HUMAN	RPL34	11023.4	12093.9	6873.13	7688.84	18488	11896	9592.79	8944.39	9862.55
P49247	P49247	RPIA_HUMAN	RPIA				628.02	967.94	1598.1	1024.66	446.025	806.049
P49257	P49257	LMAN1_HUMAN	LMAN1	4988.46	4341.89	3805.56	2683.77	6168.79	6053.49	6076.11	1397.52	4922.11
P49321	P49321	NASP_HUMAN	NASP	3512.97	1478.86	3205.98	4831.35	3986.36	11370.8	4841.65	1508.45	4637.32
P49327	P49327	FAS_HUMAN	FASN	9469.87	6958.51	9367.78	14488.9	10832	11368.9	18760.6	14696.6	16982.6
P49368	P49368	TCPG_HUMAN	CCT3	14677.5	9561.03	14615.3	30150.7	23534.6	38540	39347	30895.3	39817.7
P49406	P49406	RM19_HUMAN	MRPL19			2636.33				2664.58		3486.33
P49411	P49411	EFTU_HUMAN	TUFM	40508.8	35469.6	40663	68844.1	54706.6	54357	49704.8	59768.1	50716.4
P49419	P49419	AL7A1_HUMAN	ALDH7A1	11008.8	6561.83	9401.49	5636.33	8016.43	8340.88	6872.37	5470.6	6838.7
P49458	P49458	SRP09_HUMAN	SRP9			3130.76	3636.5	2627.36	2072.41	2060.87	3490.6	2228.56
P49585	P49585;Q9Y5K3	PCY1A_HUMAN	PCYT1A					2443.95		1566.85		
P49588	P49588	SYAC_HUMAN	AARS1	6945.04	1785.89	5155.2	5315.15	5812.6	6872.52	6564.3	4644.23	5429.7
P49589	P49589	SYCC_HUMAN	CARS1	14511.6	12886.2	14725.8	12659.1	15271.7	12258.1	6783.13	7428.45	6655.9
P49591	P49591	SYSC_HUMAN	SARS1	5223.21		5640.79	6768.27	11065.5	14554.8	6881.01	2683.49	4804.64
P49593	P49593	PPM1F_HUMAN	PPM1F	1672.29		2043.32	1018.22		1545.31	1036.53		1021.46
P49642	P49642	PRI1_HUMAN	PRIM1				2358.22	2546.48	2641.86	4146.99	3674.79	3076.58
P49643	P49643	PRI2_HUMAN	PRIM2					1898.07	1163.24	2258.86		5020.74
P49720	P49720	PSB3_HUMAN	PSMB3	2718.12	1246.32	3981.41	1013.18	1816.46	4478.99	2763.89	1206.84	4285.06
P49721	P49721	PSB2_HUMAN	PSMB2			6609.93	3881.32	12614	15703.4	7955.84	4619.76	10775.8
P49736	P49736	MCM2_HUMAN	MCM2			8546.74	1977.97	3698.16	5193.33	6724.28	3423.05	7047.68
P49748	P49748	ACADV_HUMAN	ACADVL	10088		8972.8	5925.43	6040.22	7814.69	8428.45	11963.9	9428.57
P49750	P49750	YLPM1_HUMAN	YLPM1					2250.08				
P49755	P49755	TMEDA_HUMAN	TMED10		11501.8	14125.1	4182.03	6787.15	8047.74	6384.81	5580.09	8195.52
P49756	P49756	RBM25_HUMAN	RBM25	3352.92	3828.27	3261.44	3413	3128.54	2261.53	3845.4	4595.74	3063.38
P49770	P49770	EI2BB_HUMAN	EIF2B2		991.493		3162.02	2163.86	2227.68	2152.59	3212.94	2321.48
P49773	P49773	HINT1_HUMAN	HINT1						1206.89		857.479	
P49790	P49790	NU153_HUMAN	NUP153				1435.81	2883.04	2012.39	6081.15		
P49792	A6NKT7;O14715;Q7Z3J3;P0DJD1;P49792;P0DJD0;Q	RBP2_HUMAN	RANBP2	2756.47		2394.43	4897.08	3339.69	4082.25	5197.24	6389.7	5927.68

P53582	P53582	MAP11_HUMAN	METAP1									
P53597	P53597	SUCA_HUMAN	SUCLG1		9800.06	6361.15	5355.26		3885.38	6366.7	8352.6	5975.5
P53618	P53618	COPB_HUMAN	COPB1	33496.6	34259.7	41992.5	69340.8	78235.7	74891.9	61837.8	60831.3	57251.3
P53621	P53621	COPA_HUMAN	COPA	38564.2	43112.4	34505.1	41965.8	38939.4	36502	44595.8	40382.3	43431.5
P53634	P53634	CATC_HUMAN	CTSC	9647.01	5584.49	18843.8	4793.44	2691.24	8442.88	7814.33	8746.77	9572.6
P53801	P53801	PTTG_HUMAN	PTTG1IP		902.38			856.318	1783.57	863.386		1355.06
P53985	P53985	MOT1_HUMAN	SLC16A1		2525.1	3602.21	4518.78	3355.64	3211.31	19389.1	9022.49	15378.1
P53990	P53990	IST1_HUMAN	IST1			2052.85		5175.19	4641.61	5016.25	2387.01	3855.52
P53992	P53992	SC24C_HUMAN	SEC24C	9303	7903.21	8068.29	14375	10722	16431.2	10878.3	4682.44	8324.97
P53999	P53999	TCP4_HUMAN	SUB1	43587.3	50866.6	3798.46	53255.4	46956.1	8326.93	8775.1	26611.9	7619.18
P54098	P54098	DPOG1_HUMAN	POLG	13485.7	20413		4326.29	3258.27	1724.58	1777.14	3545.15	2386.25
P54132	P54132	BLM_HUMAN	BLM		4781.27		1806.72	2474.06			1751.42	1163.83
P54136	P54136	SYRC_HUMAN	RARS1	23166.5	26020.6	23965.6	48528.8	26206.5	32558.1	18597.1	22345.5	23819.3
P54289	P54289	CA2D1_HUMAN	CACNA2D1									
P54577	P54577	SYYC_HUMAN	YARS1			4283.83	5942.98	7027.38	15752.5	6908.69	3135.15	7458.55
P54578	P54578	UBP14_HUMAN	USP14				1266.79	935.532	1593.89	1198.72		
P54619	P54619	AAKG1_HUMAN	PRKAG1					4580.44	4071.12	725.078		
P54652	P54652	HSP72_HUMAN	HSPA2	4677.55		4927.11		1739.35		1646.89	7458.32	
P54687	P54687	BCAT1_HUMAN	BCAT1				1422.7		3948.07			
P54709	P54709	AT1B3_HUMAN	ATP1B3		3398.97	6036.53	4952.81	6193.53	9786.6	20621	8377.35	21197.6
P54727	P54727	RD23B_HUMAN	RAD23B	11432.1	20503.5	4349.24	3688.12	6183.53	9827.62	5346.61	4366.76	4880.71
P54803	P54803	GALC_HUMAN	GALC									
P54819	P54819	KAD2_HUMAN	AK2	4005.81		4818.35	2545.42	2793.19	8547.84	6508.88	2211.1	5627.03
P54886	P54886	P5CS_HUMAN	ALDH18A1	9465.14	12844.9	7941.04	10385	9694.31	9272.6	20622.3	25512.7	27995.3
P54920	P54920	SNAA_HUMAN	NAPA					4487.39	3214.24	2489.91		2824.17
P55010	P55010	IF5_HUMAN	EIF5				2671.73	1964.57	2991.11	2516.2	2462.58	1785.9
P55011	P55011	S12A2_HUMAN	SLC12A2					1965.06	1560.27	17778.3	6873.71	11901.7
P55036	P55036	PSMD4_HUMAN	PSMD4			6854.71	7264.3	6566.68	10444.3	6927.4	3355.16	6963.81
P55039	P55039	DRG2_HUMAN	DRG2				4852.52			5645.85	3591.26	5064.45
P55060	P55060	XPO2_HUMAN	CSE1L	11559.2	12974.1	13201.8	36717.3	20331.3	16478.5	22237.4	37398.1	20269.1
P55061	P55061	BI1_HUMAN	TMBIM6	2315.1				1576.2	1891.81	1769.28		2027.95
P55072	P55072	TERA_HUMAN	VCP	15836	6655.35	16626.3	11358.5	20234.9	30951	24726.5	6793.01	18914.7
P55081	P55081	MFAP1_HUMAN	MFAP1				3419.2	5563.69	1801.06		2453.73	
P55084	P55084	ECHB_HUMAN	HADHB	21056.7	16487.6	16076.7	9535.33	12080.5	15062.7	24100.3	20961.6	15560.3
P55145	P55145	MANF_HUMAN	MANF	4071.15		1526.02	1812.6	2384.76	2481.54	968.157		1375.38
P55209	P55209	NP1L1_HUMAN	NAP1L1	9083.53	4847.66	9313.21	23370.2	14900.1	25268.5	11384.1	7345.47	14400.6
P55263	P55263	ADK_HUMAN	ADK					497.294	1868.8	1583.01		1053.67
P55265	P55265	DSRAD_HUMAN	ADAR	2309.6	8655.47	8746.47	1694.16	6481.68	3801.92	1812.92		1385.63
P55327	P55327	TPD52_HUMAN	TPD52							1567.28	4241.91	1964.12
P55735	P55735	SEC13_HUMAN	SEC13			7269.14					7744.06	
P55769	P55769	NH2L1_HUMAN	SNU13	2338.46	1921		2933.18	2215.26	2909.31	2484.99	2155.33	1798.58
P55786	A6NEC2;P55786	PSA_HUMAN	NPEPPS	13180.2	13701.8	13153.8	18225.6	12001.3	15728.9	8316.61	8352.62	7316.39
P55795	P55795	HNRH2_HUMAN	HNRNPH2	4191.6	2946.88	2086.35	3580.1	5273.68	3094.65	1846.52	2611.05	2495.29
P55809	P55809;Q9BYC2	SCOT1_HUMAN	OXCT1	7230.97		5524.55	3766.78	2326.97	6229.12	10779.3	7220.33	9113.8
P55884	P55884	EIF3B_HUMAN	EIF3B	8653.72	5571.11	8286.43	11511.5	9215.63	15655.2	13395.9	7210.8	12044.6
P56134	P56134	ATPK_HUMAN	ATP5MF	6818.59	7138.37	4892.9	2554.23	10875.9	4746.07	9134.3	3934.84	5275.98
P56192	P56192	SYMC_HUMAN	MARS1	15276.4	19891.9	17995.3	28832.9	36842.3	31627.6	29094.5	18496.6	21184.7
P56270	P56270	MAZ_HUMAN	MAZ					2493.04	1405.62			714.897
P56385	P56385	ATP5I_HUMAN	ATP5ME	3518.94						711.572	2328.68	
P56524	P56524	HDAC4_HUMAN	HDAC4						833.325			
P56537	P56537	IF6_HUMAN	EIF6	4794.47		7830.54	11217.1	12652.4	14308.6	12292.4	11364.3	11418.7
P56556	P56556	NDUA6_HUMAN	NDUFA6	4626.35			2166.44	2764.95	2208.33	4514.81	6865.63	5143.28
P56945	P56945	BCAR1_HUMAN	BCAR1				2728.19	3562.53	3454.09	3726.16	2092.49	3367.02
P57053	P57053;Q99879;Q5QNW6;P62807;O60814;Q99877;Q9	H2BFS_HUMAN	H2BC12L									
P57088	P57088	TMM33_HUMAN	TMEM33	8949	10136	10093.8	10273.9		4801.09	7696.51	9189.39	8925.08
P57678	P57678	GEMI4_HUMAN	GEMIN4				2674.64	3997.21	2946.81	4188.87	3532.13	4554.97
P57740	P57740	NU107_HUMAN	NUP107				1374.22	2891.1	2578.5	2839.04	801.568	1844.43
P57764	P57764	GSDMD_HUMAN	GSDMD				4993.65		3178.63	2672.04	3180.81	3192.84
P58107	P58107	EPIPL_HUMAN	EPPK1	14000.5	36723.7	12580.2		20716.3	14727.6	17433.7		19511.9
P58546	P58546	MTPN_HUMAN	MTPN				5504.37		3878.92		6766.43	3128.35
P59190	P59190	RAB15_HUMAN	RAB15				11341.4			11797.5	15291.3	15997.3
P59665;P59666	P59666;P59665	DEF1_HUMAN;DEF3_HUMAN	DEFA1B;DEFA3	19086.1	16845.1	103977	9194.3	5808.77	5182.99	5333.93	8967.15	9400.09
P59768	P59768	GBG2_HUMAN	GNG2							13283.1	4871.64	14321.6
P59998	P59998	ARPC4_HUMAN	ARPC4	8035.51	4783.23	10229.8	7302.42	5070.37	7755.85	4865.73	5522.78	6547.12
P60033	P60033	CD81_HUMAN	CD81					2708.27	2434.86	689.156		
P60174	P60174	TPIS_HUMAN	TPI1	33626.4	7721.19	45444.5	18972.7	18415.5	68535.6	33038.3	13114.3	32513.9
P60228	P60228	EIF3E_HUMAN	EIF3E	6489.45	3705.53	4865.4	9102.88	13105.3	16372.8	16356.7	5232.29	14839.6
P60468	P60468	SC61B_HUMAN	SEC61B				4006.6	2821.8	1914.12	2007.9	4391.64	2210.29
P60660	P60660	MYL6_HUMAN	MYL6	79726.4	56836.5	58289.3	269860	83569.2	71523.5	71503.2	196753	61573.4
P60709;P63261	P60709;P63261	ACTB_HUMAN;ACTG_HUMAN	ACTB;ACTG1	##########	893966	998383	1.30666E+06	2.23496E+06	1.29191E+06	1.66209E+06	954849	1.14118E+06
P60842	P60842	IF4A1_HUMAN	EIF4A1	40758.1	27858.1	49110.3	69413.4	78560	125983	114274	65316.3	91482.5
P60866	P60866	RS20_HUMAN	RPS20	114872	108579	110203	42615.3	115953	74983.5	72839.9	49376.2	66096
P60891	P21108;P60891;P11908	PRPS1_HUMAN	PRPS1	7587.86		7245.08	7825.3	5786.23	7838.32	9099.59	7757.3	9377.56
P60900	P60900	PSA6_HUMAN	PSMA6	4563.17	5594.69	11691.5	4887.14	11541.9	20454.8	13816.7	1866.6	9994.3
P60903	P60903	S10AA_HUMAN	S100A10	9629.05	9965.02	7567.4	7459.81	7891.42	11287.9	7436.63	6087.5	6352.72

[illegible]

Q 07021	Q07021	C1QBP_HUMAN	C1QBP			2713.15	4097.46	2539.29	5297.04	4251.67	4222.42	3686.28
Q 07065	Q07065	CKAP4_HUMAN	CKAP4	20422.6	14887.5	18296.6	5887.24	9945.38	8065.35	4603.53	1777.63	5023.59
Q 07157	Q07157	ZO1_HUMAN	TJP1	8136.8	7827.84	3425.09	9441.64	20957.7	15264	10755.4	8294.99	11668.5
Q 07352	Q07352	TISB_HUMAN	ZFP36L1	5121.32	4820.6	3750.41		2391.52		2573.79	1171.71	2325.84
Q 07666	Q07666;Q5VWX1	KHDR1_HUMAN	KHDRBS1	16046.5	17691.1	15781.2	13004.8	51840.9	17957.8	19159.9	13922.9	21111.1
Q 07812	Q07812	BAX_HUMAN	BAX	6511.01				5556.49	6267.02	7482.32		3670.83
Q 07820	Q07820	MCL1_HUMAN	MCL1				3173.78				699.92	545.306
Q 07864	Q07864	DPOE1_HUMAN	POLE						1305.19	2378.62	2487.68	2214.17
Q 07866	Q07866	KLC1_HUMAN	KLC1		4845.67	3730.86	6097.15	4648.46	6121.19	4246.58	2706.12	2835.16
Q 07954	Q07954	LRP1_HUMAN	LRP1	4943.67		4598.47	3211.52	3025.09	3509.25		1636.93	
Q 07955	Q07955	SRSF1_HUMAN	SRSF1	27251.3	48738.4	26016.1	13618.3	39511.1	19602.7	12913.4	19397.4	12797.8
Q 07960	Q07960	RHG01_HUMAN	ARHGAP1	7285.24		7608.67	6124.27	4078.65	6114.91	5465.28	4214.15	5492.9
Q 07973	Q07973	CP24A_HUMAN	CYP24A1						6771.75			
Q 08043	Q08043	ACTN3_HUMAN	ACTN3	5098.76			5767.36	5903.73		6104.1		9485.94
Q 08188	Q08188	TGM3_HUMAN	TGM3	16900.1	13003.5	27457.1	12191.9	4578.73	7596.21	9844.71	11065.8	16966.2
Q 08209	P16298;P48454;Q08209	PP2BA_HUMAN	PPP3CA			4480.91	2849.3		2324.41	2929.75		1933.38
Q 08211	Q08211	DHX9_HUMAN	DHX9	7280.4	10428.3	6083.66	8334.46	24852.6	11356.7	9431.86	5621.46	8426.76
Q 08257	Q08257	QOR_HUMAN	CRYZ	6325.4		4550.84	6511.61	4668.84	11169.3	5303.18	4120.91	6728.47
Q 08378	Q08378	GOGA3_HUMAN	GOLGA3	1114.35	1432.39		1436.78	2942.3	2506.16	1365.95		1318.45
Q 08379	Q08379	GOGA2_HUMAN	GOLGA2					3849.99	2153.23	3505.33	1372.46	3266.91
Q 08380	Q08380	LG3BP_HUMAN	LGALS3BP	14288	15535.7	14844.2	24757.6	17759.1	11639.3	17076.9	32306.6	21354.8
Q 08554	Q08554	DSC1_HUMAN	DSC1	25452.1	21191.4	33034.5	18283	8047.59	10460.1	9889.68	19895.9	17195.2
Q 08752	Q08752	PPID_HUMAN	PPID				1638.11	3281.24	1977.22	2424.54	1205.34	2422.82
Q 08945	Q08945	SSRP1_HUMAN	SSRP1	12021.4	16910	7755.43	10652.3	12516.5	9803.98	10008.5	11993.6	9904.7
Q 08AD1	Q08AD1	CAMP2_HUMAN	CAMSAP2			808.759	1175.85	2186.77	2325.01	415.411		417.652
Q 08AE8	Q08AE8	SPIR1_HUMAN	SPIRE1					4235.76	2552.89			
Q 08AF3	Q08AF3	SLFN5_HUMAN	SLFN5	4554.62	2767.69	4043.41	2536.14	4005.43	2447.93			
Q 08AM6	Q08AM6	VAC14_HUMAN	VAC14					7069.84	1526.98	3104.07	1049.26	2711.32
Q 08J23	Q08J23	NSUN2_HUMAN	NSUN2	7588.14	3796.62	6205.39	7827.39	10289.5	9453.96	9338.31	6582.12	8950.86
Q 09028	Q09028	RBBP4_HUMAN	RBBP4	3947.34			4200.97			1940.48	5043.44	5464.02
Q 09161	Q09161	NCBP1_HUMAN	NCBP1	4621.11	2621.92		3913.69	3549.65	2658.4	2626.43	1612.61	2979.39
Q 09328	Q09328	MGT5A_HUMAN	MGAT5							1902.21	1713.9	1500.32
[illegible]	[illegible]	[illegible]	[illegible]	[illegible]	[illegible]	[illegible]	[illegible]	[illegible]	[illegible]	[illegible]	[illegible]	[illegible]

Q 16595	Q16595	FRDA_HUMAN	FXN									
Q 16610	Q16610	ECM1_HUMAN	ECM1	6902.1	4695.18	7458.99	3644.9	1458.9	1753.51	2112.92	3910.18	3852.64
Q 16629	Q16629	SRSF7_HUMAN	SRSF7	1510.57	1538.67		784.084	4432.54	1060.24	747.34	1373.64	1041.32
Q 16630	Q16630	CPSF6_HUMAN	CPSF6	5949.35	11356	2687.45	3980.22	66825.1	7272.97	5189.27	3281.51	4969.88
Q 16637	Q16637	SMN_HUMAN	SMN2				2913.8	10589	4913.59	4334.04	7368.36	5686.21
Q 16643	Q16643	DREB_HUMAN	DBN1	4486.53		3538.96	3556.28	11407	7464.91	25044.6	4251.76	10706.1
Q 16656	Q16656	NRF1_HUMAN	NRF1	173674	81273		3231.27	22727.2	42361.3		15437.5	
Q 16658	Q16658	FSCN1_HUMAN	FSCN1	5869.92		8758.54	5548.81	4376.68	10873.4	5155.36		4618.26
Q 16666	Q16666	IF16_HUMAN	IFI16	7626.38	5478.4	5586.62	4173.25	8654.32	5789.89			
Q 16678	Q16678	CP1B1_HUMAN	CYP1B1									
Q 16698	Q16698	DECR_HUMAN	DECR1	8517.32	11074	10394	4184.46	4036.98	2373.09	2834.95	5797.47	3624.92
Q 16718	Q16718	NDUA5_HUMAN	NDUFA5				560.427	741.025	570.467	1832.11	844.296	1090.3
Q 16719	Q16719	KYNU_HUMAN	KYNU	2657.3	794.026	3982.24						
Q 16739	Q16739	CEGT_HUMAN	UGCG				1420.88	1737.05	2440.97			
Q 16762	Q16762	THTR_HUMAN	TST							2226.51		2633.47
Q 16795	Q16795	NDUA9_HUMAN	NDUFA9					5276.65	3631.01	15547.6	6656.72	13381.7
Q 16822	Q16822	PCKGM_HUMAN	PCK2	7683.67	6555.19	7488.63	5478.02	1530.48	2933.34	3254.65	4691.46	4192.56
Q 16850	Q16850	CP51A_HUMAN	CYP51A1			3140.03		2263.19	4445.62	2965.79	1775.44	4034.28
Q 16851	Q16851	UGPA_HUMAN	UGP2	5892.55		5918.7	7312.43	4268.79	3892.35	2970.71	3858.27	2905.14
Q 16881	Q9NNW7;Q16881	TRXR1_HUMAN	TXNRD1	5399.99	4229.17	7420.18	2988.63	4298.19	9356.45	2142.4	1165	2244.49
Q 16891	Q16891	MIC60_HUMAN	IMMT	34587	44493.8	28817.1	3709.97	11675.4	7369.75	7304.1	2763.15	9084.53
Q 1KMD3	Q1KMD3	HNRL2_HUMAN	HNRNPUL2	3188.42		4035.03	1532	3275.22	2746.87	3696.41	2385.32	3442.45
Q 27J81	Q27J81	INF2_HUMAN	INF2	12351.1	8803.23	8228.38	4260.43	6724.16	3155.45	6300.73	7968.63	9108.3
Q 29RF7	Q29RF7	PDS5A_HUMAN	PDS5A	6008.8	6071.83	4073.04	6216.94	5420.25	5010.27	10712.8	11682.8	7721.34
Q 2KHR3	Q2KHR3	QSER1_HUMAN	QSER1				1312.6	1033.79	635.219	1626.08	1546.06	1331
Q 2M1P5	Q2M1P5	KIF7_HUMAN	KIF7					14389	10088.9			
Q 2M3D2	Q2M3D2	EX3L2_HUMAN	EXOC3L2				5401.07	8734.59	5490.96			
Q 2NKX8	Q2NKX8	ERC6L_HUMAN	ERCC6L				1735.77	3744.03	2517.98	4181.07	1913.22	3842.98
Q 2NL82	Q2NL82	TSR1_HUMAN	TSR1				10953.3	28229.4	13893.1	11094.3	9579.59	9749.36
Q 2TAL8	Q2TAL8	QRIC1_HUMAN	QRICH1			1843.63	1530.29	1282.99	1751.55	2371.87		1670.55
Q 2TAY7	Q2TAY7	SMU1_HUMAN	SMU1			2609.84	3222.74		3058.45	6853.68		1949.31
Q 2TB90	Q2TB90	HKDC1_HUMAN	HKDC1							9514.43	17978.6	7837.1
Q 2V2M9	Q2V2M9	FHOD3_HUMAN	FHOD3	27466	22980.2		6173.83	7417.21				
Q 2VPK5	Q2VPK5	CTU2_HUMAN	CTU2				3486.68			4639.2	7096.27	3379.81
Q 32MZ4	Q32MZ4	LRRF1_HUMAN	LRRFIP1				2882.38		2200.77	1156	2823.29	1451.4
Q 32NB8	Q32NB8	PGPS1_HUMAN	PGS1	2009.38	824.863	1569.4	1721.89	2450.77	2091.59	2613.33	1513.98	3405.62
Q 32P28	Q32P28	P3H1_HUMAN	P3H1				1851.38	3339.9	3114.16	1067.61		2379.39
Q 3KQU3	Q3KQU3	MA7D1_HUMAN	MAP7D1	4914.63	5721.24	3167.03	3800.89	6579.47	5658.78	4091.76	3155.26	2867.55
Q 3LXA3	Q3LXA3	TKFC_HUMAN	TKFC			2197.49	1507.74	1348.6	2693.14	3807.62	2640.49	2973.12
Q 3MHD2	Q3MHD2	LSM12_HUMAN	LSM 12:00				1996.22	6839.32	2393.32	1048.79		1160.56
Q 3MIP1	Q3MIP1	IPIL2_HUMAN	ITPRIPL2								383.163	
Q 3MIT2	Q3MIT2	PUS10_HUMAN	PUS10									
Q 3SXY8	Q3SXY8	AR13B_HUMAN	ARL13B							2801.78	970.003	2877.63
Q 3SY69	Q3SY69	AL1L2_HUMAN	ALDH1L2	8325.08	6410.32	7300.15	9637.82	3019.58	2749.4		1178.08	
Q 3SY84	Q3SY84	K2C71_HUMAN	KRT71	2633.28		3953.76	4281.26	1530.31	3040.97		1371.14	5018.12
Q 3V6T2	A6NC98;Q3V6T2	GRDN_HUMAN	CCDC88A	4283.38			2638.52	2351.11	2134.73	3821.99	2200.17	4268.93
Q 3ZCQ 8	Q3ZCQ8	TIM50_HUMAN	TIMM50	17122.5	26598.3	16148.4	24350.8	9964.65	9326.66	14128.5	31758.9	15777.8
Q 460N5	Q460N5	PAR14_HUMAN	PARP14	6725.53	5262.54	3659.51	2449.19	3298.02	2024.02	1841.19	6034.15	1504.66
Q 4G0T1	Q4G0T1	SRCRM_HUMAN	SCART1					14606.5	23061.8	23977.4		38107.5
Q 4KMP7	Q4KMP7	TB10B_HUMAN	TBC1D10B		1513.23			4450.28	2161.71	2926.76		
Q 4KMQ 2	Q4KMQ2	ANO6_HUMAN	ANO6			726.745		1372.76	1129.82	1252.47		1038.33
Q 4V339;Q 5JTY5;Q 5RIA9;Q 8IUF	Q8IUF1;Q5RIA9;Q9BRT8;Q4V339;Q5JTY5	ZNG1A_HUMAN;ZNG1B_HUMAN;ZNG1C_H	ZNG1A;ZNG1B;ZNG1C;ZNG1E;ZNG1F						2224.18	2203.41	2199.37	2096.29
Q 52LJ0	Q52LJ0	FA98B_HUMAN	FAM98B	3583.34		2315.64	2595.13	6167.52	5775.54	5592.31	2721.17	4589.67
Q 53EP0	Q53EP0	FND3B_HUMAN	FNDC3B	8434.27	7727.33	6523.55	4337.94	3268.08	4627.32	1815.5		2453.64
Q 53EU6	Q53EU6	GPAT3_HUMAN	GPAT3				3110.44	2815.5	2884.21	1317.92	1760.57	2268.3
Q 53EZ4	Q53EZ4	CEP55_HUMAN	CEP55					5150.96	3430.81	6647.7	2289.39	6226.3
Q 53GQ 0	Q53GQ0	DHB12_HUMAN	HSD17B12	11543.6	14739.7	10352.4	11522.9	14166	11698.5	22726.1	22182.7	23293.4
Q 53H12	Q53H12	AGK_HUMAN	AGK	4441.49	3735.6	2944.15				2840.9	1760.94	3638.57
Q 53H96	Q53H96	P5CR3_HUMAN	PYCR3								1077.84	
Q 53RT3	Q53RT3	APRV1_HUMAN	ASPRV1	4484.63	4102.09	4840.64	2742.72			1037.17	7019.63	
Q 53S08;Q 9H0N0	Q9H0N0;Q53S08	RAB6C_HUMAN;RAB6D_HUMAN	RAB6C;RAB6D					913.836	1071.52	1674.61	1165.67	1007.92
Q 562E7	Q562E7	WDR81_HUMAN	WDR81	2018.85			1668.33	1550.04	1569.99	3417.39	2356.55	2972.78
Q 562R1	Q562R1	ACTBL_HUMAN	ACTBL2	253340	345610	219683	352152	616459	196834	405363	315619	308957
Q 56VL3	Q56VL3	OCAD2_HUMAN	OCIAD2							2289.47	830.376	3092.38
Q 58EX7	Q58EX7	PKHG4_HUMAN	PLEKHG4			1288.13	1082.74	2160.34	1158.62	1561.72	1636.08	
Q 58FF3	Q58FF3	ENPLL_HUMAN	HSP90B2P									658.577
Q 58FF8	Q58FF8	H90B2_HUMAN	HSP90AB2P					2855.61	4957.03	1284.91	902.889	924.576
Q 5BJD5	Q5BJD5	TM41B_HUMAN	TMEM41B				1392.5	1380.05	1056.33	4621.43	2199.58	5129.06
Q 5D862	Q5D862	FILA2_HUMAN	FLG2	29485.8	24246.7	45204.9	23064.4	5436.24	13688.2	23880.3	19312	29121.9
Q 5EB52	Q5EB52	MEST_HUMAN	MEST								5505.72	
Q 5EBL8	Q5EBL8	PDZ11_HUMAN	PDZD11	2604.91	5479.45	1976.17	843.28	4005.04	4451.3	1735.14		1894.95
Q 5GLZ8	Q5GLZ8	HERC4_HUMAN	HERC4	5858.56	8178.12	5994.21	1184.07	1173.1	1391.36	2568.23	778.686	2134.09
Q 5H9R7	Q5H9R7	PP6R3_HUMAN	PPP6R3		2168.02		3884.29	4625.5	7324.27	3678.9	3430.84	5053.3
Q 5HYK3	Q5HYK3	COQ5_HUMAN	COQ5					3231.47	2842.85	3532.59	2448.32	4599.91
Q 5JPH6	Q5JPH6	SYEM_HUMAN	EARS2	3546.02	5050.58	4676.64	2275.8	2881.52	2916.83	3405.68	2680.12	4179.27
Q 5JRX3	Q5JRX3	PREP_HUMAN	PITRM1	675.549		1182.06	768.044		866.966			
Q 5JSZ5	Q5JSZ5	PRC2B_HUMAN	PRRC2B	3361.12			1714.32	5369.43	3409.28	843.877		3299.63
Q 5JTH9	Q5JTH9	RRP12_HUMAN	RRP12				2056.63	3747.29	3330.73	1045.29	1490.1	2421.84
Q 5JTV8	Q5JTV8	TOIP1_HUMAN	TOR1AIP1	2643.14				2566.69	3822.62	5230.35	2215.53	4537.08
Q 5JTZ9	Q5JTZ9	SYAM_HUMAN	AARS2		2558.74	3891.87	3324.51	2123.11	2762.63	4564.26	4379.18	3410.97
Q 5JVF3	Q5JVF3	PCID2_HUMAN	PCID2		2178.24		644.374	3570.06	2074.7	835.427	977.809	867.235
Q 5JWF2	Q5JWF2	GNAS1_HUMAN	GNAS	6213.72		11421	14118.8	24273.9	23096.2	47326.1	11251.4	42144.4
Q 5M775	Q5M775	CYTSB_HUMAN	SPECC1				2411.29	5466.76	1938.51	2039.38	342.518	2153.33
Q 5SRE5	Q5SRE5	NU188_HUMAN	NUP188				3964.11	2800.03	3186.5	3040.9	5472.92	2982.21
Q 5SSJ5	Q5SSJ5	HP1B3_HUMAN	HP1BP3	6498.99	5739.07	3924.18		2024.48	2059.95		2160.84	
Q 5ST30	Q5ST30	SYVM_HUMAN	VARS2						829.921	1297.06		941.997
Q 5SW79	Q5SW79	CE170_HUMAN	CEP170	2900.06	3048.06	2064.33	3897.93	4822.03	2865.85	1201.93	1456.89	841.941
Q 5SY16	Q5SY16	NOL9_HUMAN	NOL9					3088.6	949.291	870.276		
Q 5SZB4	Q5SZB4	CI050_HUMAN	C9orf50							6352.27		
Q 5T0B9	Q5T0B9	ZN362_HUMAN	ZNF362						11797.7	3043.3		
Q 5T160	Q5T160	SYRM_HUMAN	RARS2	4496.71	4615.82	3959.71	2300.47	3237.59	2476.41	2094.53	2578.22	3847.49
Q 5T1M5	Q5T1M5	FKB15_HUMAN	FKBP15			371.748	1605.33	1689.82	1109.42	1058.21	1294.96	917.579
Q 5T447	Q5T447	HECD3_HUMAN	HECTD3				1946.17	3333.63	1270.68	2004.73	1218.98	2748.18
Q 5T4S7	Q5T4S7	UBR4_HUMAN	UBR4		3451.54	5366.83	4645.71	2728.22	2576.9	3868.97	5612.47	3463.96
Q 5T5P2	Q5T5P2	SKT_HUMAN	KIAA1217	6287.44			5876.46	7782.34	5816.8	3530.38	2261.45	4173.12
Q 5T6F2	Q5T6F2	UBAP2_HUMAN	UBAP2				4614.12	2281.65	2747.28	2990.06	3557.85	2862.62
Q 5T6V5	Q5T6V5	QNG1_HUMAN	QNG1				1862.11		1532.17			
Q 5T749	Q5T749	KPRP_HUMAN	KPRP	77636.2	61107.7	115034	50446.5	21719.1	30793	52918.8	33718.2	70201.1
Q 5T750	Q5T750	XP32_HUMAN	XP32	22945.4	17123.3	35608.7	16588.6	4842	4207.7	9083.27	13187.7	18911
Q 5T8P6	Q5T8P6	RBM26_HUMAN	RBM26	1659.75	1480.2	1279.37	1453.28	2887.98	1134.02	1489.09	2253.14	1545.19
Q 5T9A4	Q5T9A4	ATD3B_HUMAN	ATAD3B	6860.18	8679.38	4064.13		2406.2		7310.45	1286.88	7959.64
Q 5TA45	Q5TA45	INT11_HUMAN	INTS11				1541.86	5377.83	3672.69	5629.11	6397.38	5420.48
Q 5TEC6	Q5TEC6;P84243;P68431;Q71DI3;Q16695;Q6NXT2	H37_HUMAN	H3-7	312529	311259	193871	154288	291448	150696	53868	83331.4	42303.4
Q 5UIP0	Q5UIP0	RIF1_HUMAN	RIF1					627.9	588.634	3058.57	1157.42	1332.25
Q 5VIR6	Q5VIR6	VPS53_HUMAN	VPS53	2434.67		1598.34	2336.86	2134.94	796.637	2234.33	3713.75	2251.09
Q 5VSL9	Q5VSL9	STRP1_HUMAN	STRIP1				2340.59	2348.4	2428.19	2275.52	1586.21	3781.3
Q 5VT25	Q5VT25	MRCKA_HUMAN	CDC42BPA				2240.2	3066.41	2257.31	2704.61	2417.72	1901.2
Q 5VUA4	Q5VUA4	ZN318_HUMAN	ZNF318		824.345			1201.97	674.224	985.352		774.128
Q 5VV42	Q5VV42	CDKAL_HUMAN	CDKAL1							1591.59	1882.76	1522.92
Q 5VYK3	Q5VYK3	ECM29_HUMAN	ECPAS	6985.9	6231.87	6273.87	10871.6	7460.79	7908.87	9107.54	9464.73	9125.64
Q 5VYS8	Q5VYS8	TUT7_HUMAN	TUT7	3558.34		1784.42	1470.05			1319.25		2147.56
Q 5VZE5	Q5VZE5	NAA35_HUMAN	NAA35				1276.13	1914.81	1135.9	1249.63	712.685	1353.2
Q 5XPI4	Q5XPI4	RN123_HUMAN	RNF123						1129.92	802.336		1017.79
Q 63HN8	Q63HN8	RN213_HUMAN	RNF213			1947.1	2023.9	1504.18	1493.37	1271.16	2351.67	1753.4
Q 63ZY3	Q63ZY3	KANK2_HUMAN	KANK2	9898.61	10086.3	8195.13	5509.12	4508.63	3953.45	2964.83	4699.01	3671.4
Q 643R3	Q643R3	LPCT4_HUMAN	LPCAT4		1199.74		1320.04	9295.88	3690.62	2877.07	1090.79	8322.57
Q 658Y4	Q658Y4	F91A1_HUMAN	FAM91A1				2090.37		1585.37	2147.15	2328.24	2736.31
Q 66K74	Q66K74	MAP1S_HUMAN	MAP1S	2830.02	1989.22	2563.87	3306.94	1972.48	3392.31	2840.83	1765.65	3221.04
Q 68CQ 7	Q68CQ7	GL8D1_HUMAN	GLT8D1		2747.42	1541.55		1994.29	1268.34	2625.29	787.491	2598.76
Q 68CZ2	Q63HR2;Q68CZ2;Q9HBL0	TENS3_HUMAN	TNS3			5923	3408.17	6107.66	1896.12	9293.78	9526.27	9005.7
Q 69YL0	Q69YL0	NCAS2_HUMAN	NCBP2AS2					3482.39	665.376	837.621		
Q 69YQ 0	Q69YQ0	CYTSA_HUMAN	SPECC1L	6087.56		8660.76	4567.99	26529.8	7924.16	2973.79		3458.71
Q 6AI08	Q6AI08	HEAT6_HUMAN	HEATR6					2504.09	1848.46	696.358		
Q 6AWC2	Q6AWC2	WWC2_HUMAN	WWC2				718.264	1251.06	996.543			750.278
Q 6DD88	Q6DD88	ATLA3_HUMAN	ATL3	9668.57	9273.45	9956.62	8997	7915.64	6252.4	2964.4	3451.41	2475.48
Q 6DKJ4	Q6DKJ4	NXN_HUMAN	NXN				732.985	1774.48	1817.83	739.081		2062.2
Q 6IA86	Q6IA86	ELP2_HUMAN	ELP2					1739.61	2059.54	1231.83		740.842
Q 6IAA8	Q6IAA8	LTOR1_HUMAN	LAMTOR1	4666.38	5343.49	3883.43	10041.8	16810.1	21773.9	49458.8	17307	39105.7
Q 6IBS0	Q6IBS0	TWF2_HUMAN	TWF2				1310.14	2471.75	1985.34	1137.02		1470.58
Q 6IQ 23	Q6IQ23	PKHA7_HUMAN	PLEKHA7				1554.74	8024.45	4336.75	5604.94	4782.34	7411.63
Q 6JQ N1	Q6JQN1	ACD10_HUMAN	ACAD10					996.127	1185.15	2112.12	1496.09	2356.06
Q 6KB66	Q6KB66	K2C80_HUMAN	KRT80	9185.45	12801.9	17743.7	11462.4	41415.7	24811.2	41256.3	15663.1	41248.9
Q 6L8Q 7	Q6L8Q7	PDE12_HUMAN	PDE12				2444.51		1647.82	1119.65	1313.96	1843.8
Q 6MZP7	Q6MZP7	LIN54_HUMAN	LIN54	1382.95			647.987	3594.96	1012.57			
Q 6NSI4	Q6NSI4	RADX_HUMAN	RADX								2283.79	
Q 6NSJ5	Q6NSJ5	LRC8E_HUMAN	LRRC8E				1389.3	2701.92	2038.53	1006.66		1090.21
Q 6NTF9	Q6NTF9	RHBD2_HUMAN	RHBDD2							1332.33	418.442	1463.17
Q 6NUM9	Q6NUM9	RETST_HUMAN	RETSAT	1882.32		1590.31	1993.75	1773.04	1728.53	1270.81	1525.09	1518.42
Q 6NUQ 4	Q6NUQ4	TM214_HUMAN	TMEM214	5771.96	5435.72	3262.61	3572.47			2096.43	3243.77	2169.95
Q 6NXE6	Q6NXE6	ARMC6_HUMAN	ARMC6	4578.77	4129.83	4280.37	7363.33	5794.46	5638.77	7344.42	9830.37	8272.39
Q 6NXG1	Q6NXG1	ESRP1_HUMAN	ESRP1							2900.45	4442.03	
Q 6NXR4	Q6NXR4	TTI2_HUMAN	TTI2				2914.99	1363.29	857.093	1389.11	2230.55	2281.86
Q 6NYC8	Q6NYC8	PPR18_HUMAN	PPP1R18	3973.21	4765.14	2509.79		4739.45	3883.12	3979.11		
Q 6NZ67;Q 6P582	Q6NZ67;Q6P582	MZT2A_HUMAN;MZT2B_HUMAN	MZT2A;MZT2B					847.783	720.746			419.804
Q 6NZI2	Q6NZI2	CAVN1_HUMAN	CAVIN1	8572.08	6088.42	8804.55	12096.4	12702.8	11773.1	11338.6	6217.5	10054.3
Q 6NZY4	Q6NZY4	ZCHC8_HUMAN	ZCCHC8					1385.36	834.949	679.489		
Q 6P087	Q6P087	RUSD3_HUMAN	RPUSD3		897.619			1016.96	1042.3	1988.34	1085.45	1639.98
Q 6P158	Q6P158	DHX57_HUMAN	DHX57					6513.28	1888.53			
Q 6P1J9	Q6P1J9	CDC73_HUMAN	CDC73					2178.5	1570.3	1506.08	980.828	1071.97
Q 6P1L8	Q6P1L8	RM14_HUMAN	MRPL14	4537.11	3216.21	5279.27	3572.14	2111.81	1963.2	2928.58	6412.33	2557.99
Q 6P1M3	Q6P1M3	L2GL2_HUMAN	LLGL2							1999.79	1026.93	1225.93
Q 6P1N0	Q6P1N0	C2D1A_HUMAN	CC2D1A	2691.26	3371.8	2527.41	2747.57	1562.86	1779.06	1928.4	951.812	1891
Q 6P2C8	Q6P2C8	MED27_HUMAN	MED27				1433.4	1871.52	1743.21	1079.01		411.554
Q 6P2E9	Q6P2E9	EDC4_HUMAN	EDC4	22886.6			4412.29	11550.9	6862.37	9280.43	6307.01	7837.55
Q 6P2H3	Q6P2H3	CEP85_HUMAN	CEP85		2068.16		1943.78	1818.67	1963.24	4050.92	1414.9	3835.95
Q 6P2Q 9	Q6P2Q9	PRP8_HUMAN	PRPF8		5820.41		5397.65	12024.9	6834.61	8336.47	10045.8	7453.88
Q 6P3W7	Q6P3W7	SCYL2_HUMAN	SCYL2			1566.76	1499.75	2726.31	2893.94	1412.42	1155.08	1564.08
Q 6P3X3	Q6P3X3	TTC27_HUMAN	TTC27				3602.53	7436.85	4930.63	6714.43	2828.52	5289.67
Q 6P4A8	Q6P4A8	PLBL1_HUMAN	PLBD1	3983.83		5799.3	2701.49		2766.08	1874.97	3410.82	2554.86
Q 6P5R6	Q6P5R6	RL22L_HUMAN	RPL22L1	7387.61	9907.79	9486.25	22332.2	53413.8	31519.6	2001.83	7404.05	1160.63
Q 6P996	Q6P996	PDXD1_HUMAN	PDXDC1				6192.8	6361.22	3771.14	4814.99	9131.06	5338.57
Q 6P9B6	Q6P9B6	MEAK7_HUMAN	MEAK7				922.188	6965.47	7177.71	2405.71		2281.19
Q 6P9B9	Q6P9B9	INT5_HUMAN	INTS5				4952.84	2196.99	1153.5	2614.29	8179.95	
Q 6PD62	Q6PD62	CTR9_HUMAN	CTR9				1094.36	2041.98	1688.65	1953.67	985.389	1581.94
Q 6PD74	Q6PD74	AAGAB_HUMAN	AAGAB					2876.04	3938.84	3690.71		733.35
Q 6PGP7	Q6PGP7	SKI3_HUMAN	SKIC3	4370.6	4543.4	4380.83	4622.22	6068.5	4063.44	4673.18	4531.87	3115.5
Q 6PI48	Q6PI48	SYDM_HUMAN	DARS2	2063.91	2967.25	1406.95	1401.64	4752	7309.35	2844.13	2591	2722.62
Q 6PIU2	Q6PIU2	NCEH1_HUMAN	NCEH1	6614.92	3790.44	6229.78	4188.33	2784.33	2848.18	7782.68	6590.1	7703.48
Q 6PJG2	Q6PJG2	MDEAS_HUMAN	MIDEAS					4809.44	2130.72	1266.09		
Q 6PJG6	Q6PJG6	BRAT1_HUMAN	BRAT1				3275.54	2354.73	1159.45	2840.37	2729.55	3935.06
Q 6PKG0	Q6PKG0;Q659C4	LARP1_HUMAN	LARP1	12856.9	14866.2	7958.06	8826.25	48714.4	15134.2	10337.3	6661.7	9596.08
Q 6PUV4	Q6PUV4	CPLX2_HUMAN	CPLX2									
Q 6Q EF8	Q6QEF8	CORO6_HUMAN	CORO6				2864.93	7016.39	4112.31			
Q 6R327	Q6R327	RICTR_HUMAN	RICTOR	2130.78	2187.22	1119.57	2194.54	2123.13	2020.05	2528.83	2230.86	3266.83
Q 6RFH5	Q6RFH5	WDR74_HUMAN	WDR74					2781.76	1494.65	3210.13	2012.72	2890.93
Q 6UB35	Q6UB35	C1TM_HUMAN	MTHFD1L			7471.2	8119.3		8918.56	12219.5	12576.1	12735.7
Q 6UN15	Q6UN15	FIP1_HUMAN	FIP1L1	7692.02		5458.04	5044.11	3653.08	3064.92	3320.27	7852.71	3316.79
Q 6UWJ1	Q6UWJ1	TMCO3_HUMAN	TMCO3			8344.87		13505.6		13585	8178.15	13767.8
Q 6UWP8	Q6UWP8	SBSN_HUMAN	SBSN	8877.94	6517.15	17106.4	7028.25	10852.2	7190.95	6950.24	8536.71	9384.76
Q 6UX53	Q6UX53	TMT1B_HUMAN	TMT1B						843.656	3807.25	4978.53	4164.05
Q 6VY07	Q6VY07	PACS1_HUMAN	PACS1				759.599	1825.49	2417.2	256.211	1097.89	612.338
Q 6W2J9	Q6W2J9	BCOR_HUMAN	BCOR									
Q 6WCQ 1	Q6WCQ1	MPRIP_HUMAN	MPRIP	24606	24919.5	20798.9	73066.7	91229.8	61013.6	10568.3	10225.7	9930.99
Q 6XE24	Q6XE24	RBMS3_HUMAN	RBMS3	43829.9	30291.1		12126.8	8820.68	748.817			
Q 6XQ N6	Q6XQN6	PNCB_HUMAN	NAPRT	1551.09					993.947	1484.8	2265.83	965.49
Q 6Y7W6	Q6Y7W6	GGYF2_HUMAN	GIGYF2	3964.96	3438.81	3900.01	3091.72	5000.71	3395.83	2584.11	3567.47	2616.85
Q 6YHK3	Q6YHK3	CD109_HUMAN	CD109	3584.49		1798.74	2368.17	2574.9	2127.4	2560.74	2064.37	3588.85
Q 6YN16	Q6YN16	HSDL2_HUMAN	HSDL2			1759.17	4346.42	10951.7	5226.68	4240.45	5127.67	4139.57
Q 6ZMP0	Q6ZMP0	THSD4_HUMAN	THSD4									
Q 6ZN17	Q6ZN17	LN28B_HUMAN	LIN28B				9821.75	23841.9	17240.9	3721.42		
Q 6ZN28	Q6ZN28	MACC1_HUMAN	MACC1				1157.94			2451.84	1211.24	2119.57
Q 6ZNB6	Q6ZNB6	NFXL1_HUMAN	NFXL1	826.397	974.039		1134.64	352.106	511.682	2151.43	1684.26	2152.28
Q 6ZRV2	Q6ZRV2	FA83H_HUMAN	FAM83H	2129.1	6487.83	2782.75	1541.13	6299.46	5815.7	13475.1	2661.06	14676.9
Q 6ZS17	Q6ZS17	RIPR1_HUMAN	RIPOR1	2583.35			4249.87	5128.94	5674.87	4472.57	2466.99	3902.53
Q 6ZSR9	Q6ZSR9	YJ005_HUMAN						1544.63	1281.35			
Q 6ZTW0	Q6ZTW0	TPGS1_HUMAN	TPGS1							2618.86		
Q 6ZVD8	Q6ZVD8	PHLP2_HUMAN	PHLPP2	353768	342339	268583	303835	82266.4	84578.1	160183	311727	144702
Q 6ZVX7	Q6ZVX7	FBX50_HUMAN	NCCRP1	10028.6		13367.8	7613.91		4557.96	5522.72	9749.15	9002.19
Q 6ZW31	Q6ZW31	SYDE1_HUMAN	SYDE1	3153.81	2297.01	2071.21	2726.33					
Q 70CQ 2	Q70CQ2	UBP34_HUMAN	USP34	79996.8	77070.4	99183.8	47174.4	7751.29	8199.19	13219.9	64596.5	19535.4
Q 70J99	Q70J99	UN13D_HUMAN	UNC13D				9717.14	10670.7	11497			689.093
Q 70SY1	Q70SY1	CR3L2_HUMAN	CREB3L2									
Q 71RC2	Q71RC2	LARP4_HUMAN	LARP4	3404.22	4808.78		3659.74	12041.8	6441.11	4413.86	5345.08	6004.3
Q 71SY5	Q71SY5	MED25_HUMAN	MED25	2892.9		2893.83	3805.12	793.723	1423.78	3125.79	4591.23	3452.62
Q 71UM5	Q71UM5	RS27L_HUMAN	RPS27L	10308.7	11906.5	10310.4	16307.5	16542.3	12284.5	7234.31	10770.4	7944.41
Q 75N03	Q75N03	HAKAI_HUMAN	CBLL1	1484.26	2320.18	2095.42		3345.91	2342.86	2930.07	804.511	2782.31
Q 75Q N2	Q75QN2	INT8_HUMAN	INTS8								1982.7	
Q 7KZF4	Q7KZF4	SND1_HUMAN	SND1	7848.39	4625.85	8195.99	12853.4	13979.3	16696.6	12955.3	6988.35	11220.2
Q 7KZI7	Q7KZI7	MARK2_HUMAN	MARK2				1659.02	2563	1480.79	3144.79	2676.1	2593.66
Q 7L014	Q7L014	DDX46_HUMAN	DDX46	4892.41	4730.05	2423.61	3701.73	3295.51	2881.04	2934.73		3465.48
Q 7L0Y3	Q7L0Y3	TM10C_HUMAN	TRMT10C				2482.09	3906.47	3132.59	2681.4	1624.66	3912.57
Q 7L1Q 6	Q7L1Q6	5MP2_HUMAN	BZW1	11341.6	996.127	7047.39	2755.82	3059.79	5722.53	3297.93	3939.92	4046.4
Q 7L2E3	Q7L2E3	DHX30_HUMAN	DHX30	6788.23	6845.87	6235.93	4526.76	12056.1	6730.82	6397.96	4951.97	4741.32
Q 7L2H7	Q7L2H7	EIF3M_HUMAN	EIF3M				1985.52	6092.36	7759.36	5175.54	1284.69	5796.02
Q 7L3T8	Q7L3T8	SYPM_HUMAN	PARS2		1577.95		1262.95	3378.81	1898.18	3136.4	2509.23	2448.89
Q 7L576	Q7L576	CYFP1_HUMAN	CYFIP1	5986.96	8639.23	4084.74	8240.49	8494.92	8073.74	4478.39	4554.8	3519.49
Q 7L592	Q7L592	NDUF7_HUMAN	NDUFAF7	3061.48			2042.14			777.242	5696.75	990.325
Q 7L5N1	Q7L5N1	CSN6_HUMAN	COPS6	957.011		1122.55	2317.97		2881.4	2918.28		1945.66
Q 7L8L6	Q7L8L6	FAKD5_HUMAN	FASTKD5				6833.16	5072.97	3243.26	11001.6	10638	10477.4
Q 7L9B9	Q7L9B9	EEPD1_HUMAN	EEPD1				2114.2	10855.2	3751.52	45667.1	14256.7	48524.2
Q 7RTP6	Q7RTP6	MICA3_HUMAN	MICAL3				1413.91	14186.1	6163.31	1767.32		
Q 7RTS7	Q7RTS7	K2C74_HUMAN	KRT74	25951.3	18892.6	44866.9	20314.9	77799.3	57327.3	40582.8	31202	43299.3
Q 7Z2E3	Q7Z2E3	APTX_HUMAN	APTX		2744.84		2019.53	4237.91	1872			844.975
Q 7Z2K8	Q7Z2K8	GRIN1_HUMAN	GPRIN1				2266.79	2808.52	2620.64	8059.28	3478.55	7055.84
Q 7Z2W4	Q7Z2W4	ZCCHV_HUMAN	ZC3HAV1	4647.92	6495.95	7543.55	13355.2	10314.7	5932.32	3475.03	6086.85	4089.13
Q 7Z2Y5	Q7Z2Y5	NRK_HUMAN	NRK				1410.91	3133.69	2177.87			
Q 7Z3B4	Q7Z3B4	NUP54_HUMAN	NUP54				2846.21			781.607	455.952	
Q 7Z3U7	Q7Z3U7	MON2_HUMAN	MON2	5888.56		4171.01	7772.8	7049.36	6110.87	5864.5	4014.72	5309.33
Q 7Z3Y8	Q7Z3Y8;Q7Z3Z0	K1C27_HUMAN	KRT27			8514.06	4036.33		4780.49		2376.76	6810.2
Q 7Z417	Q7Z417	NUFP2_HUMAN	NUFIP2	6670.45		6586.11	11526.5	16536.1	4952.31	3360.94	5444.38	4476.61
Q 7Z422	Q7Z422	SZRD1_HUMAN	SZRD1						672.779	1104.26	713.362	1214.97
Q 7Z434	Q7Z434	MAVS_HUMAN	MAVS	3158.21	3155.55	3047.35		928.066	2207.28	2040.2		2986.05
Q 7Z460	Q7Z460	CLAP1_HUMAN	CLASP1			899.726	998.526	876.793	1100.23	1124.56	2726.61	1089.53
Q 7Z478	Q7Z478	DHX29_HUMAN	DHX29			1078.54		3260.94	1685.51	1118.97		
Q 7Z494	Q7Z494	NPHP3_HUMAN	NPHP3					1964.01	1229.28			
Q 7Z4H7	Q7Z4H7	HAUS6_HUMAN	HAUS6				4624.85	2481.99	2159.89	2131.93	1802.47	2200.87
Q 7Z4H8	Q7Z4H8	PLGT3_HUMAN	POGLUT3					3782.76	3286.9			
Q 7Z4Q 2	Q7Z4Q2	HEAT3_HUMAN	HEATR3	3375.75	4184.87	2916.98	8363.45	13766.5	6079.71	11512.1	14221.2	8947.13
Q 7Z4S6	Q7Z4S6	KI21A_HUMAN	KIF21A				686.089	1171.06	1017.03	1443.76	826.982	1152.38
Q 7Z4W1	Q7Z4W1	DCXR_HUMAN	DCXR			8183.51	2307.85	838.313	7060.85	8651.54	6982.59	8783.91
Q 7Z5G4	Q7Z5G4	GOGA7_HUMAN	GOLGA7			1983.88			3814.34	12684.9	2735.41	7746.39
Q 7Z5L9	Q7Z5L9	I2BP2_HUMAN	IRF2BP2							2182.95	1624.93	2168.35
Q 7Z628	Q7Z628	ARHG8_HUMAN	NET1							1222.13		2041.44
Q 7Z6I8	Q7Z6I8	CE024_HUMAN	C5orf24				3204	5514.08	2659			
Q 7Z6J0	Q7Z6J0	UBE3D_HUMAN	UBE3D						584.115	1164.78		
Q 7Z6W7	Q7Z6W7	DNJB7_HUMAN	DNAJB7	377625	234649	240279	10579	45632.5	27096.5	36076.3	14736.5	44844.6
Q 7Z6Z7	Q7Z6Z7	HUWE1_HUMAN	HUWE1				3360.98	2044.5	987.293	4759.07	4637.26	5213.38
Q 7Z739	Q7Z739;Q9BYJ9	YTHD3_HUMAN	YTHDF3				3465.11	8626.15	4275.96	2652.38	3203.77	3740.52
Q 7Z794	Q7Z794	K2C1B_HUMAN	KRT77	25925.4	25257	47215.6	20537.7	5209.98	12044	6420.3	21645.2	27009.6
Q 7Z7A3	Q7Z7A3	CTU1_HUMAN	CTU1				11895	9406.41	3429.89	5474.45	9925.69	7883.32
Q 86SE5	Q86SE5	RALYL_HUMAN	RALYL				6937.98	1879.93	977.066	2446.89	6848.13	5279.71
Q 86SQ 0	Q86SQ0	PHLB2_HUMAN	PHLDB2	6876.1	8172.13	4888.39	4180.36	8338.83	4168.06		1344.52	
Q 86T24	Q86T24	KAISO_HUMAN	ZBTB33		847.944					608.099	1270.59	1024.15
Q 86TG7	Q86TG7	PEG10_HUMAN	PEG10				863.847	2599.65	1027.57	2107.86	3567.24	1890.09
Q 86TV6	Q86TV6	TTC7B_HUMAN	TTC7B				1849.28	3378.99	3214.07	5083.85		5231.89
Q 86U38	Q86U38	NOP9_HUMAN	NOP9		6323.69		1687.63	5721.61		2444.61	1408.49	2767.14
Q 86U90	Q86U90	YRDC_HUMAN	YRDC				3322.21	2174.17	2203.54	1892.02		1911.42
Q 86UE4	Q86UE4	LYRIC_HUMAN	MTDH	5125.41	4561.82	4411.85	3463.51	2893.84	3399.76	5675.06	3039.06	5798.92
Q 86UE8;Q 9UKI8	Q9UKI8;Q86UE8	TLK1_HUMAN;TLK2_HUMAN	TLK1;TLK2					1075.39	791.529	477.968		510.292
Q 86UK7	Q86UK7	ZN598_HUMAN	ZNF598					1227	1300.97			
Q 86UP2	Q86UP2	KTN1_HUMAN	KTN1	3942.46			2913.76	3695.44	2976.5	3358.59	1804.43	3214.66
Q 86US8	Q86US8	EST1A_HUMAN	SMG6					1103.12		1261.43		
Q 86UU1	Q86UU1	PHLB3_HUMAN	PHLDB3		721.049		1163.07	2701.62	1479.59	894.071	532.11	987.094
Q 86UW9	Q86UW9	DTX2_HUMAN	DTX2					1151.37	598.35	974.857		1018.86
Q 86UY6	Q86UY6	NAA40_HUMAN	NAA40					2633.27	2385.59	2344.69		1837.6
Q 86V48	Q86V48	LUZP1_HUMAN	LUZP1		3923.41		3163.79	5397.87	4449.19			1455.68
Q 86V81	Q86V81	THOC4_HUMAN	ALYREF	13247.8	10723.6	16305.6	18721.7	37303.2	25709.4	21673.5	16047.8	17244.2
Q 86VI3	Q86VI3	IQGA3_HUMAN	IQGAP3	1610.37	4898.2		6352.28	6155.46	4763.01	3931.7	3754.96	4586.86
Q 86VN1	Q86VN1	VPS36_HUMAN	VPS36	3014.16		2312.58	2681.64	1633.12	1175.94	1330.64	497.521	1317.73
Q 86VP6	Q86VP6	CAND1_HUMAN	CAND1	18063.1	10345.5	17084.2	35326.8	30383.2	27164.1	26984.7	19877.8	25164.2
Q 86VR2	Q86VR2	RETR3_HUMAN	RETREG3				1204.73	4146.48	2843.59	2362.71		
Q 86VS8	Q86VS8	HOOK3_HUMAN	HOOK3	55451.4	34071.5	37887.2	49904.3	18590.9	14483.9	16057.1	32775.6	15634.9
Q 86VY4	Q86VY4	TSYL5_HUMAN	TSPYL5									
Q 86W42	Q86W42	THOC6_HUMAN	THOC6					1332.69	637.236	412.977		891.903
Q 86W92	Q86W92;Q8ND30	LIPB1_HUMAN	PPFIBP1			5500.38	6633.25	5429.7	4074.14	5642.98	5208.53	6511.83
Q 86WB0	Q86WB0	ZC3C1_HUMAN	ZC3HC1				410.296	595.148	452.047	938.278	375.723	832.069
Q 86WJ1	Q86WJ1	CHD1L_HUMAN	CHD1L	7866.87	13198.1	3695.88	7113.58	9516.72	4104.41	5061.06	6009.8	4787.78
Q 86X29	Q86X29	LSR_HUMAN	LSR				1904.44	1616.18	3329.31	20280.7	4882.89	14748
Q 86X55	Q86X55	CARM1_HUMAN	CARM1	3342.97		2818.47	1592.7	1817.05	1558.52	3053.8	3171.35	2617.42
Q 86XI2	Q86XI2	CNDG2_HUMAN	NCAPG2		1299.64		1902.08	2057.84	1207.98	4099.33	3029	4054.7
Q 86XP3	Q86XP3	DDX42_HUMAN	DDX42				1907.64	6841.94	7674.99	7357.24	696.788	7049.66
Q 86XZ4	Q86XZ4	SPAS2_HUMAN	SPATS2				1505.71	1247.07	1208.16	1822.69	1652.97	1992.64
Q 86Y07	Q86Y07	VRK2_HUMAN	VRK2		2595.08			2110.97	1222.47	2061.72		897.006
Q 86Y39	Q86Y39	NDUAB_HUMAN	NDUFA11	2958.02	2447.07		1925.69			3683.18	1304.11	1411.2
Q 86Y56	Q86Y56	DAAF5_HUMAN	DNAAF5	8525.37	4107.82		8405.4	6816.73	7482.92	14359.6	9627.91	15155.8
Q 86Y79	Q86Y79	PTH_HUMAN	PTRH1	5464.7	5693	5303.12				1615.76		3017.49
Q 86Y82	Q86Y82	STX12_HUMAN	STX12	3974.6	2792.39	2903.61	1408.11	2175.15	3698.55	2969.33		2388.01
Q 86YP4	Q86YP4	P66A_HUMAN	GATAD2A				2422.12	521.557	636.415	1131.96	2114.93	890.054
Q 86YQ 8	Q9HCH3;Q86YQ8;Q8IYJ1	CPNE8_HUMAN	CPNE8				458.648	2089.46	1986.29			
Q 86YT6	Q86YT6	MIB1_HUMAN	MIB1					2365.93	1763.08	1559.48		1907.08
Q 86YV9	Q86YV9	HPS6_HUMAN	HPS6						1978.29	6117.03	5004.04	4239.9
Q 86YW0	Q86YW0	PLCZ1_HUMAN	PLCZ1		7834.25	3788.86		6811.06	2875.55		1977.93	3849.44
Q 86YZ3	Q86YZ3	HORN_HUMAN	HRNR	151845	95918.7	187715	136718	37387	46333.6	71166.1	83917.2	113881
Q 8IUC1	Q8IUC1	KR111_HUMAN	KRTAP11-1									
Q 8IUC4	Q8IUC4;A8MT19	RHPN2_HUMAN	RHPN2		10337.8		7109.89	6768.26	5828.89			
Q 8IUD2	Q8IUD2	RB6I2_HUMAN	ERC1	3348.56	3677.21	2157.16	2928.39	3167.28	2209.7	1046.39		879.386
Q 8IUE6	Q96QV6;P16104;Q8IUE6	H2A2B_HUMAN	H2AC21	10263.1	12870.6	9885.55	11487.1	8072.09	5816.92			
Q 8IUR7	Q8IUR7	ARMC8_HUMAN	ARMC8				5771.17			1121.13	5317.59	2118.91
Q 8IUX4	Q8IUX4	ABC3F_HUMAN	APOBEC3F		2715.39			4335.2	1694.7			2369.88
Q 8IV08	Q8IV08	PLD3_HUMAN	PLD3		5238.1			1993.34	2208.67		733.045	237.641
Q 8IV36	Q8IV36	HID1_HUMAN	HID1									
Q 8IV63	Q8IV63	VRK3_HUMAN	VRK3		4231.29		10187.2	4087.24	3782.67	1079.4	2870.06	
Q 8IVF2	Q8IVF2	AHNK2_HUMAN	AHNAK2					1226				
Q 8IVF7	Q8IVF7	FMNL3_HUMAN	FMNL3	3348.84		1987.15	6351.96	4825.62	4933.17	3993.49	6432.31	3131.28
Q 8IVL6	Q8IVL6	P3H3_HUMAN	P3H3		1576.71	1267.32	729.434	5744.24	4755.5	1432.59	1024.27	
Q 8IVM0	Q8IVM0	CCD50_HUMAN	CCDC50						2164.35			
Q 8IVS2	Q8IVS2	FABD_HUMAN	MCAT			2817.68	1138.11		994.41	3474.58	2765.03	3018.11
Q 8IVT2	Q8IVT2	MISP_HUMAN	MISP		11313.7	5034.63			1800.13	12147.3	3091.14	9146.63
Q 8IVT5	Q8IVT5	KSR1_HUMAN	KSR1					2311.27				
Q 8IVW6	Q8IVW6	ARI3B_HUMAN	ARID3B				698.674	7082.07	2030.85			
Q 8IW19	Q8IW19	APLF_HUMAN	APLF	8757.43	10318.8		15229.3					
Q 8IW75	Q8IW75	SPA12_HUMAN	SERPINA12	4719.72	3843.61	5380.7	3195.71	2482.82	2919.35	2325.35		3060.53
Q 8IWE2	Q8IWE2	NXP20_HUMAN	FAM114A1	2420.44	1189.32	1795.03	536.992	926.722	1797.18			
Q 8IWE4	Q8IWE4	DCNL3_HUMAN	DCUN1D3					3987.95	3245.74	7567.52	7457.75	8959.72
Q 8IWN7	Q8IWN7	RP1L1_HUMAN	RP1L1	7351.67	5497.41	8435.39	13715.4		2375.01	8685.96	17786.9	9452.28
Q 8IWS0	Q8IWS0	PHF6_HUMAN	PHF6					7068.83	5273.46	12141.9	7042.03	14247
Q 8IWT6	Q8IWT6	LRC8A_HUMAN	LRRC8A				1158.11	1939.56	3427.93	3963.73	1799.84	2166.01
Q 8IWU2	Q8IWU2	LMTK2_HUMAN	LMTK2				1938.89	3773.27	1916.15	5617.76	1774.79	5037.8
Q 8IWV7	Q8IWV7	UBR1_HUMAN	UBR1	853.718	3709.44		1464.99	1780.39	2698.44	689.74		790.943
Q 8IWX8	Q8IWX8	CHERP_HUMAN	CHERP				2333.51	4741.36	2421.28	2528.57	2593.72	2261.64
Q 8IWZ3	Q8IWZ3	ANKH1_HUMAN	ANKHD1				6246.41	5180.11	2495.73	1484.38	5923.18	1902.37
Q 8IX12	Q8IX12	CCAR1_HUMAN	CCAR1				1298.57	849.946	533.991	828.732	795.632	674.259
Q 8IXB1	Q8IXB1	DJC10_HUMAN	DNAJC10				2222.74	2273.86	1855.8	2781.52	1497.27	2704.69
Q 8IXH7	Q8IXH7	NELFD_HUMAN	NELFCD					4966.6	2534.6	5090.46		9076.33
Q 8IXM3	Q8IXM3	RM41_HUMAN	MRPL41		5316.56		2566		2636.05	3584.86	2355.02	
Q 8IXS6	Q8IXS6;C9JA33	PALM2_HUMAN	PALM2					1891.79	1710.74	16334	2675.75	14901.8
Q 8IXS8	Q8IXS8	HYCC2_HUMAN	HYCC2							3518.73		3379.32
Q 8IY17	Q8IY17	PLPL6_HUMAN	PNPLA6	4122.5	5548.87	3902.72	2391.64	6496.27	3903.13	5952.96	2821.05	6882.93
Q 8IY21	Q8IY21	DDX60_HUMAN	DDX60				867.557	2035.16	2482.98			
Q 8IY22	Q8IY22	CMIP_HUMAN	CMIP				1780.28		1927.59	6061.59	3368.49	6918.93
Q 8IY67	Q8IY67	RAVR1_HUMAN	RAVER1	4305.72	8563.77	5647.33	7585.96	6121.95	10939.4	11142.3	4738.43	12561.3
Q 8IY81	Q8IY81	SPB1_HUMAN	FTSJ3					5540.81	1430.24	482.178		447.487
Q 8IYB3	Q8IYB3	SRRM1_HUMAN	SRRM1	1693.36	2868.4	569.35	813.957	4658.04	1292.96	1393.88	1567.6	925.813
Q 8IYB8	Q8IYB8	SUV3_HUMAN	SUPV3L1				1978.16	1267.19	2038.16	2474.06	2327.4	2874.15
Q 8IYI6	Q8IYI6	EXOC8_HUMAN	EXOC8		951.188	1591.27	1460.14	1370.84	1225.32	1238.81	965.54	1233.11
Q 8IYT3	Q8IYT3	CC170_HUMAN	CCDC170				35041.4	29635	68161.5	263411	24380.9	196565
Q 8IZS2	Q8IZS2	CHSS2_HUMAN	CHPF	3649.17	1285.72	2715.36	2126.33	1623.8	1234.4	9462.96	6643.1	11968

Q 8IZ69	Q8IZ69	TRM2A_HUMAN	TRMT2A				5987.43	4621.76	1513.46	758.987	7742.79	1695.87
Q 8IZ83	Q8IZ83	A16A1_HUMAN	ALDH16A1	1352.1	1583.96	919.365	2676.58	1719.64	1785.95	1092.91	440.733	1403.23
Q 8IZH2	Q8IZH2	XRN1_HUMAN	XRN1	13767.7	21168.1		5327.68	5157.91	3022.9	3210.31	2355.28	4374.59
Q 8IZL8	Q8IZL8	PELP1_HUMAN	PELP1			1220.94	5263.82	9749.89	3307.96	1275.05	495.65	1490.37
Q 8IZP0	Q9NYB9;Q8IZP0	ABI1_HUMAN	ABI1			5774.49	3504.28	9759.97	7713.26	6438.74		6724.27
Q 8IZV5	Q8IZV5	RDH10_HUMAN	RDH10	1840.1	1887.98	1717.62			672.599	4885.73	2827.63	5093.49
Q 8IZW8	Q8IZW8	TENS4_HUMAN	TNS4							3769.86	1577	3594.89
Q 8N0X7	Q8N0X7	SPART_HUMAN	SPART				1851.64	2169.77	1611.63			
Q 8N0Z3	Q8N0Z3	SPICE_HUMAN	SPICE1					1627				
Q 8N122	Q8N122	RPTOR_HUMAN	RPTOR		3575.42	2811.79	4283.87	2490.14	2303.79	4796.94	6540.5	4563.73
Q 8N163	Q8N163	CCAR2_HUMAN	CCAR2	2934.52	2796.94	4968.22	5211.39	11134.2	10811.3	16901.7	9361.12	12799.9
Q 8N183	Q8N183	NDUF2_HUMAN	NDUFAF2				2608.55			7315.53	5191.24	4551.22
Q 8N1B4	Q8N1B4	VPS52_HUMAN	VPS52			2206.37	2315.11		1294.1	2083.62	764.753	2405.23
Q 8N1F7	Q8N1F7	NUP93_HUMAN	NUP93		5938.96	4663.87	12206	10112.7	7755.15	14787.8	13982.3	14967.7
Q 8N1G4	Q8N1G4	LRC47_HUMAN	LRRC47	4526.4	3024.59	4076.02	5113.66	5413.28	7751.86	3327.62	2564.06	2784
Q 8N1N4	Q8N1N4	K2C78_HUMAN	KRT78	87681.1	65749.8	114765	69888.1	31504.8	35897.4	44951.5	68818	63450.5
Q 8N201	Q8N201	INT1_HUMAN	INTS1	3608.29		2732.67	4119.17	11744.5	2622.63	4786.53	7689.8	4668.83
Q 8N283	Q8N283	ANR35_HUMAN	ANKRD35				2868.35			3302.93		
Q 8N335	Q8N335	GPD1L_HUMAN	GPD1L					1925.59				
Q 8N392	Q8N392	RHG18_HUMAN	ARHGAP18				986.804	1512.55	2058.6	4718.73		1866.7
Q 8N3C0	Q8N3C0	ASCC3_HUMAN	ASCC3	15249.9	11532.4	2167.36	13266.6	11357.2	6257	6327.21	13750.4	6136.76
Q 8N3E9	Q8N3E9	PLCD3_HUMAN	PLCD3				3902.8	3113.27	3241.74	11852.7	5158.7	12681.2
Q 8N3R9	Q8N3R9	PALS1_HUMAN	PALS1					3385.82	1330.13	1770.4		1470.89
Q 8N3V7	Q8N3V7	SYNPO_HUMAN	SYNPO	14359.7	6748.5	12266.4						
Q 8N488	Q8N488	RYBP_HUMAN	RYBP				1884.63					
Q 8N4C8	Q8N4C8	MINK1_HUMAN	MINK1			1791.69	897.074	1151.75	1622.38	1117.38	2045.93	1744.88
Q 8N4V1	Q8N4V1	EMC5_HUMAN	MMGT1	3486.91	3260.87	2472.51	969.818	1204.08	1509.04	5540.81	1645.12	5822.23
Q 8N4X5	Q8N4X5	AFIL2_HUMAN	AFAP1L2				2153.48	2684.32	2958.8			
Q 8N556	Q8N556	AFAP1_HUMAN	AFAP1	1540.47		2215.23	1330.03	591.248	709.148	1039.6	1024.86	755.256
Q 8N5K1	Q8N5K1	CISD2_HUMAN	CISD2		5679.76			4867.53	4965.45	7764.92		9323.86
Q 8N5M9	Q8N5M9	JAGN1_HUMAN	JAGN1					2946.92	3109.92	3672.05		3516.67
Q 8N680	Q8N680	ZBTB2_HUMAN	ZBTB2		4327.71			567.074				
Q 8N684	Q8N684	CPSF7_HUMAN	CPSF7				9182.58	69175.8	12024.1	6472.91	7158.36	5642.93
Q 8N6L1	Q8N6L1	KTAP2_HUMAN	KRTCAP2	2014.01			966		427.57	1468.08	1858.05	772.029
Q 8N6R0	Q8N6R0	EFNMT_HUMAN	METTL13		6643.68	5462.05	6925.41	5725.96	5381.29	5800.64	7357.96	5220.17
Q 8N6T3	Q8N6T3	ARFG1_HUMAN	ARFGAP1				5203.44	3085.14	4081.09	1244.39		1199.38
Q 8N766	Q8N766	EMC1_HUMAN	EMC1				1052.62	1195.84	1170.49	1679.97	1204.18	1529
Q 8N7H5	Q8N7H5	PAF1_HUMAN	PAF1				2339.7	3526.07	4457.55	2675.6	1949.28	
Q 8N7R7	Q8N7R7	CCYL1_HUMAN	CCNYL1				4760.87	5909.67	4104.95	6721.1	6268.42	5386.11
Q 8N884	Q8N884	CGAS_HUMAN	CGAS		935.565		7315.28	30899.8	16396.8			
Q 8N8S7	Q8N8S7	ENAH_HUMAN	ENAH	1433.42		1448.27	378.378	1154.35	1894.21	287.632		201.297
Q 8N9B5	Q8N9B5	JMY_HUMAN	JMY					1737.85	1572.04	2704.07		1188.44
Q 8N9N2	Q8N9N2	ASCC1_HUMAN	ASCC1	8802.17	4012.18		3829.46	2363.1	1117.28	1239.12	3843.6	
Q 8NB37	Q8NB37	GALD1_HUMAN	GATD1						1483.84	793.192		
Q 8NB90	Q8NB90	SPAT5_HUMAN	SPATA5		3694.32		4567.31	4027.35	2754.49	2142.62	2429.64	2168.6
Q 8NBF2	Q8NBF2	NHLC2_HUMAN	NHLRC2			2739.18	1715.98	2890.39	3266.15	1651.07		1440.96
Q 8NBJ5	Q8NBJ5	GT251_HUMAN	COLGALT1				3663.86	5070.75	6109.18			
Q 8NBJ7	Q8NBJ7	SUMF2_HUMAN	SUMF2	3845.18	1591.12	2805.29	2950.82	1734.52	2259.34	1769.45	1057.38	2213.89
Q 8NBN7	Q8NBN7	RDH13_HUMAN	RDH13				830.59	1211.85	1423.13	1686.33	780.258	1403.45
Q 8NBQ5	Q8NBQ5	DHB11_HUMAN	HSD17B11	1386.39	2473.12	1697.84	604.674	3520.74	3986.25	5113.19	1504.15	1769.7
Q 8NBS9	Q8NBS9	TXND5_HUMAN	TXNDC5	8018.17		6709.22	2934.1	14058	21182.4	7669.89	2831.27	6089.66
Q 8NBU5	Q8NBU5	ATAD1_HUMAN	ATAD1					4964.87	3256.84	2485.26		2637.47
Q 8NBX0	Q8NBX0	SCPDL_HUMAN	SCCPDH	4382.64	3049.43	5987.33	2716.22	6511.84	6534.59	5367.76	2120.64	4698.95
Q 8NC51	Q8NC51	PAIRB_HUMAN	SERBP1	18771.4	15674.5	14693	28346.8	43090.1	36787	24603.2	20967.1	23062.3
Q 8NC56	Q8NC56	LEMD2_HUMAN	LEMD2	3868.03	1859.29		1362.85	4203.9	2333.3	2473.45		2252.73
Q 8NCA5	Q8NCA5	FA98A_HUMAN	FAM98A	7780.48	12249	5824.21	4448	31847.7	12294.5	6542.45	3071.84	5811.73
Q 8NCN5	Q8NCN5	PDPR_HUMAN	PDPR	1984.73			2410.13	2938.51	3825.71	1142.4		
Q 8NCR9	Q8NCR9	CLRN3_HUMAN	CLRN3						1623.56	22106.6	9747.66	24671.6
Q 8NCW5	Q8NCW5	NNRE_HUMAN	NAXE					334.025	745.227			
Q 8NCY6	Q8NCY6	MSD4_HUMAN	MSANTD4		2959.69			1638.34	884.603			
Q 8ND56	Q8ND56	LS14A_HUMAN	LSM14A	2323.15		2522.33	933.58	3648.82	1555.94		717.708	878.569
Q 8ND76	Q8ND76	CCNY_HUMAN	CCNY				5603.06	3617.11	4973.32	18948	6913.2	19270.4
Q 8NDI1	Q8NDI1	EHBP1_HUMAN	EHBP1						891.809	1855.73		1653.59
Q 8NDN9	Q8NDN9	RCBT1_HUMAN	RCBTB1		4377.73					6853.83		6738.94
Q 8NDT2	Q8NDT2	RB15B_HUMAN	RBM15B			4008.68	12907.3	32214.7	22847.7	23224.6	15122.3	33637.2
Q 8NDV7	Q8NDV7	TNR6A_HUMAN	TNRC6A		1260.65		2665.35	16309.2	3143.74			3094.1
Q 8NE71	Q8NE71	ABCF1_HUMAN	ABCF1				3707.26	4479.86	4407.67	3991.82	4767.01	4903.08
Q 8NE86	Q8NE86	MCU_HUMAN	MCU	1381.88		1719.32	1186.18	4752.36	3065.51	5776.5	1306.99	4104.96
Q 8NEB9	Q8NEB9	PK3C3_HUMAN	PIK3C3	3858.36		3441.36	5427.08	8995.11	7304.71	2378.64	2578.9	1591.56
Q 8NEU8	Q8NEU8	DP13B_HUMAN	APPL2				1651.85	2237.68	2062.33	1153.73		1007.43
Q 8NEV8	Q8NEV8	EXPH5_HUMAN	EXPH5			2585.69		7379.19	7372.66	3700.66		3000.67
Q 8NEX9	Q8NEX9	DR9C7_HUMAN	SDR9C7	6076.18	5987.7	10152.2	1517.08	2472.87	4834	3792.45	3675.19	5526.58
Q 8NEZ5	Q8NEZ5	FBX22_HUMAN	FBXO22					4120.06	3092.32	1424.92		
Q 8NF37	Q8NF37	PCAT1_HUMAN	LPCAT1			4419.18	2238.33			4960.94	5462.99	5345.21
Q 8NFH3	Q8NFH3	NUP43_HUMAN	NUP43							357.786		
Q 8NFH5	Q8NFH5	NUP35_HUMAN	NUP35				955.097	1051.47	1038.51	1219.49		1582.53
Q 8NFJ5	Q8NFJ5	RAI3_HUMAN	GPRC5A	15331.1				6846.33	4027.36	10804.2	2708.44	10085.3
Q 8NFT2	Q8NFT2	STEA2_HUMAN	STEAP2		890.735			659.109		1416.66		1889.89
Q 8NFV4	Q8NFV4	ABHDB_HUMAN	ABHD11						1513.21	2885.83	954.391	3107.13
Q 8NFW8	Q8NFW8	NEUA_HUMAN	CMAS					1474.41	1471.33	1724.93		2335.63
Q 8NFZ0	Q8NFZ0	FBH1_HUMAN	FBH1	45879.9	82058.8		8699.85	15921.1	3966.18	4200.19	18721.7	
Q 8NG31	Q8NG31	KNL1_HUMAN	KNL1			27296.2	10482	7048.08	5016.26	7231.63	15272.2	14192.3
Q 8NHG7	Q8NHG7	SVIP_HUMAN	SVIP					5986.84	4636.43	18811	1355.63	11389.7
Q 8NHH9	Q8NHH9	ATLA2_HUMAN	ATL2				629.086	1459.1	755.808	734.799		1228.66
Q 8NHM4	Q8NHM4	TRY6_HUMAN	PRSS3P2	4883.57	10656.4	23575	5143.95	5893.31	10191.2	1577.2		2579.87
Q 8NHM5	Q8NHM5	KDM2B_HUMAN	KDM2B					3561.46				
Q 8NHQ9	Q8NHQ9	DDX55_HUMAN	DDX55					7014.41	1724.22	1367.63		
Q 8NI27	Q8NI27	THOC2_HUMAN	THOC2					5787.41	2513.82	6037.83	4177.5	4753.77
Q 8NI35	Q8NI35	INADL_HUMAN	PATJ							5412.28	986.054	4593.81
Q 8NI36	Q8NI36	WDR36_HUMAN	WDR36					944.006				
Q 8NI60	Q8NI60	COQ8A_HUMAN	COQ8A	27504.4			12343.5			4488.67	10713.8	3701.32
Q 8TAD7	Q8TAD7	OCC1_HUMAN	OCC1				14790.5	53749.1	69575.4	55075.3	11630.7	76544.6
Q 8TAG9	Q8TAG9	EXOC6_HUMAN	EXOC6			3302.2	2933.69	2185.6	2066.51	4238.44	4576.65	4979.82
Q 8TAM2	Q8TAM2	TTC8_HUMAN	TTC8							2073.62		3470
Q 8TB72	Q8TB72	PUM2_HUMAN	PUM2					11763.9	1878.72	1211.04		
Q 8TBC4	Q8TBC4	UBA3_HUMAN	UBA3						1959.65	566.338		913.161
Q 8TBP5	Q8TBP5	F174A_HUMAN	FAM174A		24980.5	23425	32655.7				31434	
Q 8TC07	Q8TC07	TBC15_HUMAN	TBC1D15	4606.74	1796.86	4439.35	5578.36	3155.61	2624.09	2170.78	6038.62	5442.23
Q 8TC12	Q8TC12	RDH11_HUMAN	RDH11	5404.3	6094.47	7824.33	2894.58	2487.72	4200.98	5470.55	1979.9	4781.02
Q 8TCA0	Q8TCA0	LRC20_HUMAN	LRRC20						1734.26			
Q 8TCB0	Q8TCB0	IFI44_HUMAN	IFI44				4344.69	2477.07	4921.33			
Q 8TCG1	Q8TCG1	CIP2A_HUMAN	CIP2A				3122.77	3582.29	2578.53	4422.56	2822.92	5648.26
Q 8TCJ2	Q8TCJ2	STT3B_HUMAN	STT3B		4391.33	3453.11	2288.91	3011.1	1917.97	5286.58	2812.96	5573
Q 8TCS8	Q8TCS8	PNPT1_HUMAN	PNPT1	2258.17	1212.15		2141.83	3783.95	6524.33	3539.67	3313.79	3626.76
Q 8TCT8	Q8TCT8	SPP2A_HUMAN	SPPL2A				1115.31	3116.67	2481.95	1990.35		1038.1
Q 8TCT9	Q8TCT9	HM13_HUMAN	HM13			1661.71	4971.29		3836.09	5665.56	7925.06	8536.18
Q 8TD19	Q8TD19	NEK9_HUMAN	NEK9	4983.43	2910.64	3178.81	3270.35	2522.07	2318.6	1328.57	2818.18	1786.8
Q 8TD57	Q8TD57	DYH3_HUMAN	DNAH3			18900.1			15722.3	18368		
Q 8TDL5	Q8TDL5	BPIB1_HUMAN	BPIFB1			5871.69		6469.37		1903.71		
Q 8TDX7	Q9HC98;Q8TDX7	NEK7_HUMAN	NEK7		1029.19	1859.49	1670.04	1831.3	3366.93	2114.7	511.139	2149.44
Q 8TDZ2	Q8TDZ2	MICA1_HUMAN	MICAL1				527.962	1255.8	831.399	412.141		768.419
Q 8TE02	Q8TE02	ELP5_HUMAN	ELP5					1613.18	1629.86	3353.8	1794.61	2602.45
Q 8TE73	Q8TE73	DYH5_HUMAN	DNAH5		18390.8	7878.33	60051.1	28686.2	30922	26878.8	21336.7	
Q 8TEA8	Q8TEA8	DTD1_HUMAN	DTD1									1454.89
Q 8TED1	Q8TED1	GPX8_HUMAN	GPX8	3897.18	9483.09	4062.46	2226.4		2599.97		1897.41	
Q 8TEL6	Q8TEL6	TP4AP_HUMAN	TRPC4AP			1059.8		1277.75	774.793	1013.03		1158.94
Q 8TEM1	Q8TEM1	PO210_HUMAN	NUP210					2696.37		5856.37	7897.88	5538.18
Q 8TEQ6	Q8TEQ6	GEMI5_HUMAN	GEMIN5				8735.28	11204	10846	7302.52	4262.47	7358.07
Q 8TER5	Q8TER5	ARH40_HUMAN	ARHGEF40				3251.02					1426.78
Q 8TEW0	Q8TEW0	PARD3_HUMAN	PARD3		2917.62			2682.82	1692.74	1256.89		
Q 8TEX9	Q8TEX9	IPO4_HUMAN	IPO4		5456.2	4718.75	4736.93	14233	12805.8	15071.7	19615.5	24628.2
Q 8TF66	Q8TF66	LRC15_HUMAN	LRRC15				2451.67					
Q 8TF68	Q8TF68	ZN384_HUMAN	ZNF384	8877.87	4569.77		922.905	2998.03	1328.39			
Q 8TF72	Q8TF72	SHRM3_HUMAN	SHROOM3		256.535		188.494	1011.94	527.503	203.832		
Q 8WTT2	Q8WTT2	NOC3L_HUMAN	NOC3L					2649		673.532		
Q 8WTW3	Q8WTW3	COG1_HUMAN	COG1	3093.61			4604.9	4661.13	5484.89	3612.02	4123.39	3970
Q 8WUF5	Q8WUF5	IASPP_HUMAN	PPP1R13L		3518.48	4721.35	2509.69	2794.72	2941.2	1316.81	986.016	1276.7
Q 8WUI4	Q8WUI4	HDAC7_HUMAN	HDAC7				1872.87	3449.46	4124.36	1669.98		1977.3
Q 8WUJ3	Q8WUJ3	CEMIP_HUMAN	CEMIP	5236.86	3658.97	7321.48				5951.94	4968.33	9256.6
Q 8WUK0	Q8WUK0	PTPM1_HUMAN	PTPMT1	182061	183231	209192		3535.01	2371.11	6836.34	4148.06	8139.1
Q 8WUM0	Q8WUM0	NU133_HUMAN	NUP133				1566.27	3275.16	2504.46	1966.88		1030.24
Q 8WUM4	Q8WUM4	PDC6I_HUMAN	PDCD6IP	11874.1	9236.45	14337.6	10527.1	8548.57	11079.7	11834.2	6984.84	9048.22
Q 8WUW1	Q8WUW1	BRK1_HUMAN	BRK1				843.725	675.656	1531.45		456.159	451.022
Q 8WV24	Q8WV24	PHLA1_HUMAN	PHLDA1			3061.7	1727.66		1448.81	4578.53	2907.21	4681.78
Q 8WV41	Q8WV41	SNX33_HUMAN	SNX33	2463.31	4029.18	1947.75	1891.13		1897.49			1138.72
Q 8WV74	Q8WV74	NUDT8_HUMAN	NUDT8								1149.46	1396.3
Q 8WVB6	Q8WVB6	CTF18_HUMAN	CHTF18		317.922		1617.98	3298.68	2141.5	3248.41	1791.72	1997.15
Q 8WVC6	Q8WVC6	DCAKD_HUMAN	DCAKD			5034.62	3339.67	1950.15	2340.6	3157.43	4309.92	4672.45
Q 8WVM8	Q8WVM8	SCFD1_HUMAN	SCFD1	5022.46	5083.19	3518.42	4922.33	2693.98	3369.74	2774.83	2305.56	3392.76
Q 8WVV4	Q8WVV4	POF1B_HUMAN	POF1B	5215.99		4232.61	3691.81	2252.24	2052.03	14310.7	8153.25	12556.4
Q 8WVV9	Q8WVV9	HNRLL_HUMAN	HNRNPLL					3350.46	3080.96	3077.57		2839.51
Q 8WVX9	Q8WVX9	FACR1_HUMAN	FAR1	3368.77			3362.51			4813.17	6869.37	3085.19
Q 8WVY7	Q8WVY7	UBCP1_HUMAN	UBLCP1					1163.5	1679.09	375.019		
Q 8WW12	Q8WW12	PCNP_HUMAN	PCNP	1452.57			930.059	799.357	2083.89	938.901	588.645	843.773
Q 8WW33	Q8WW33	GTSF1_HUMAN	GTSF1				5198.31		6576.24			
Q 8WWI1	Q8WWI1	LMO7_HUMAN	LMO7	13500.8	15186.7	14485.1	2309.28	6883.65	2733.93	6986.48	11481	7518.63
Q 8WWI5	Q8WWI5	CTL1_HUMAN	SLC44A1					2906.49	3963.43	1195.42		790.468
Q 8WWM7	Q8WWM7	ATX2L_HUMAN	ATXN2L	7156.31	6890	5738.01	13298.3	46726.8	14401.8	7011.2	5374.76	7277.76
Q 8WWZ7	Q8WWZ7	ABCA5_HUMAN	ABCA5				1733.17	3739.52		4060.59		4159.4
Q 8WX92	Q8WX92	NELFB_HUMAN	NELFB				1516.87	1857.34	2418.52	860.317	566.261	1831.29
Q 8WX93	Q8WX93	PALLD_HUMAN	PALLD	4075.88	4151.13	2864.73	2874.3	4827.76	5382.2			
Q 8WX94;Q 9NX02	Q9NX02;Q8WX94	NALP2_HUMAN;NALP7_HUMAN	NLRP2;NLRP7									
Q 8WXE1	Q8WXE1	ATRIP_HUMAN	ATRIP				1864.58				4733.77	
Q 8WXE9	Q8WXE9	STON2_HUMAN	STON2	1566.7		2683.28	162725	18672.9	52109.2	552312	120842	476457
Q 8WXF1	Q8WXF1	PSPC1_HUMAN	PSPC1	2785.43		3795.7	2285.31	66261.1	9213.68	4369.15	2170.42	4647.86
Q 8WXG6	Q8WXG6	MADD_HUMAN	MADD				421.71		1407.97	1421.18		1777.47
Q 8WXI7	Q8WXI7	MUC16_HUMAN	MUC16		2400.8			11897.8	14931.5	8848.2	2800.62	3167.16
Q 8WXI9	Q8WXI9	P66B_HUMAN	GATAD2B	743.805	680.06	229.814	2641.81		345.282	1224.11	2209.49	1451.06
Q 8WXR4	Q8WXR4	MYO3B_HUMAN	MYO3B		23425.4	9110.06	16412.6	6785.49	9685.75	55268.5	8179.4	
Q 8WXW3	Q8WXW3	PIBF1_HUMAN	PIBF1					998.47	1337.46	1395.62		2739.44
Q 8WXX5	Q8WXX5	DNJC9_HUMAN	DNAJC9	5398.79	7631.21	5760.53	6415.18	9621.47	5376.32	4944.47	4532.65	4356.71
Q 8WY22	Q8WY22	BRI3B_HUMAN	BRI3BP								832.352	
Q 8WYL5	Q8WYL5	SSH1_HUMAN	SSH1	830.693	1272.34	864.219		2872.52	1945.42	1205.28		
Q 8WZ42	Q8WZ42	TITIN_HUMAN	TTN		16565.2	4446.17	8201.53		2094.95		15912	1920.69
Q 8WZA9	Q8WZA9	IRGQ_HUMAN	IRGQ					3748.98	4467.61	1060.03		1382.43
Q 92466	Q92466	DDB2_HUMAN	DDB2		12736.7		4863.72	11121.1	5457.53	1621.17	2978.07	2594.7
Q 92485	Q92485	ASM3B_HUMAN	SMPDL3B				1898.52	2313.05	1510.1	4942.01	1514.77	6325.95
Q 92499	Q92499	DDX1_HUMAN	DDX1			3546.43	7269.99	24150.4	8691.63	3650.82	4692.45	4205.58
Q 92504	Q92504	S39A7_HUMAN	SLC39A7	10486.5	10086.1	13029	3807.62	5553.28	3874.74	6960.28	4055.07	6649.25
Q 92506	Q92506	DHB8_HUMAN	HSD17B8				1240.26			1263.11		
Q 92522	Q92522	H1X_HUMAN	H1-10	3338.34	1658.55	2306.15	1118.78	1609.54		2226.37	2631.36	2622.38
Q 92526	Q92526	TCPW_HUMAN	CCT6B	11980.3	4856.98	13328	25708.8	12447.8	30969.2	14664.6		27230.1
Q 92530	Q92530	PSMF1_HUMAN	PSMF1			2250.83	1917.26	2180.47	3948.72	9007.93	2946.26	7527.94
Q 92536	Q92536	YLAT2_HUMAN	SLC7A6							1513.16	338.252	1063.17
Q 92538	Q92538	GBF1_HUMAN	GBF1	3404.92	3515.49	3400.25	2033.79	1947.84	1797.78	2863.51	2195.24	2839.69
Q 92541	Q92541	RTF1_HUMAN	RTF1					183.016	357.406	260.344		264.723
Q 92542	Q92542	NICA_HUMAN	NCSTN	6652.56		8927.05	7771.13	6314.81	6937.88	3121.25	4681.99	2533.42
Q 92546	Q92546	RGP1_HUMAN	RGP1		511.755			951.563	1001.73	446.562		471.987
Q 92552	Q92552	RT27_HUMAN	MRPS27	4604.22	4940.33	4541.9	3223.67	7855.7	6717.83	4736.96	4628.83	3904.8
Q 92575	Q92575	UBXN4_HUMAN	UBXN4			2257.83	4339.96	8929.98	1326.77	2896.2	4103.75	2499.22
Q 92597	Q92597	NDRG1_HUMAN	NDRG1	14168.8	19147.4	21000.9	16936.3		18409.8			
Q 92598	Q92598	HS105_HUMAN	HSPH1	3859.07		6519.38	11328.9	10114.2	20019.9	10441	6881.12	11548.9
Q 92599	Q92599	SEPT8_HUMAN	SEPTIN8				2416.34	1594.33	2544.43	990.06		1047.54
Q 92600	Q92600	CNOT9_HUMAN	CNOT9				1267.38	2699.63	1636.36	2616.35	3109.9	2345.37
Q 92609	Q92609	TBCD5_HUMAN	TBC1D5	4769.93	2244	4188.4	2994.04			2517.55	3857.83	1666.23
Q 92614	Q92614	MY18A_HUMAN	MYO18A	4185.06			8307.88	8855.12	5579.48	10018.8	7586.3	7680.56
Q 92615	Q92615	LAR4B_HUMAN	LARP4B	5634.68	4290.88	5745.74	5827.94	15356.9	7504.83	10847	10711.5	11882.7
Q 92616	Q92616	GCN1_HUMAN	GCN1	24396.4	23074	18942.4	43201.9	30879.2	27774	33897	39301.4	33826
Q 92620	Q92620	PRP16_HUMAN	DHX38					4370.2	2978.11	5831.56	4415.47	3261.15
Q 92621	Q92621	NU205_HUMAN	NUP205		3122.1	3460.42	3870.19	3461.89	2975.23	5411.26	8722.91	4805.78
Q 92643	Q92643	GPI8_HUMAN	PIGK		1657.21		641.418	3074.14	1551.29	3914.88	1468.43	4114.98
Q 92673	Q92673	SORL_HUMAN	SORL1				984.437				2095.65	
Q 92688	Q92688	AN32B_HUMAN	ANP32B	4559.34		5794.43	4824.5	3062.68	8532.12	5244.11	2305.95	5235.93
Q 92734	Q92734	TFG_HUMAN	TFG				2215.05	7208	5953.33	3916.21		5475.03
Q 92766	Q92766	RREB1_HUMAN	RREB1				64.7892	1329.34	415.614	492.257		592.578
Q 92769	Q92769	HDAC2_HUMAN	HDAC2					3009.74	2231.72	3259.09		3210.53
Q 92783	Q92783	STAM1_HUMAN	STAM				2359.46	2225.45	1876.46			1299.89
Q 92786	Q92786	PROX1_HUMAN	PROX1							1563.53	822.8	1603.04
Q 92796	Q92796	DLG3_HUMAN	DLG3					914.441	379.142	1787.63		846.521
Q 92797	Q92797	SYMPK_HUMAN	SYMPK					4028.2	1383.77	3270.08		4753.14
Q 92804	Q92804	RBP56_HUMAN	TAF15	2788.7	6640.59	1616.03	2042.69	146829	64153.8	8412.79	1169.76	3258.77
Q 92820	Q92820	GGH_HUMAN	GGH	5588.25	4001.69	7523.53	2659.76	1929.84	5240.35	5038.49	3900.91	5952.6
Q 92828	Q92828	COR2A_HUMAN	CORO2A				22191	41341.2	16184.1	15881.5	7164.23	10779.4
Q 92841	Q92841	DDX17_HUMAN	DDX17	12014.4	11856.7	13543.3	9827.47	13231.3	8316.93	4658.7	5942.29	4851.78
Q 92878	Q92878	RAD50_HUMAN	RAD50		2563.33	2842.38	3005.52	3712.33	3001.94	2082.62	1915.83	2158.42
Q 92882	Q92882	OSTF1_HUMAN	OSTF1					877.898	1849.58	2049.61		2654.69
Q 92888	Q92888	ARHG1_HUMAN	ARHGEF1	7114.19	5646.21	6094.31	9218.4	10830	9197.62	3469.53		4053.66
Q 92890	Q92890	UFD1_HUMAN	UFD1	3921.93	1816	3840.94		3069.52	5547.7	1877.45		2500.51
Q 92896	Q92896	GSLG1_HUMAN	GLG1						1499.8	3138.54	2918.13	3662.22
Q 92900	Q92900	RENT1_HUMAN	UPF1	13878.8	14419.8	5673.17	8857.74	23333.3	11047.9	7438.09	5359.21	8217.33
Q 92905	Q92905	CSN5_HUMAN	COPS5				2220.68	2742.54	5213.22	2539.08	1389.57	2498.55
Q 92922	Q92922	SMRC1_HUMAN	SMARCC1					15715.1	11711.1	17581.4		17958.4
Q 92930	Q92930	RAB8B_HUMAN	RAB8B					2016.31	1808.63	1419.4		750.775
Q 92945	Q92945	FUBP2_HUMAN	KHSRP	11065.9	7978.79	10206.5	12770.7	9194.83	12134.4	10098.2	6134.76	8977.37
Q 92947	Q92947	GCDH_HUMAN	GCDH	6468.32	6484.05	10197.5	6104.78	4317.28	5563.7	10554.7	10778.3	11653.1
Q 92973	Q92973	TNPO1_HUMAN	TNPO1	13470	17425.7	15310	17108.3	10768.7	14934.8	12827	8918.25	11099.3
Q 92974	Q92974	ARHG2_HUMAN	ARHGEF2	4831.59	6381.53	6496.2	6252.52	6783.27	8911.74	3423.95		4077.06
Q 92979	Q92979	NEP1_HUMAN	EMG1	6234.61	5919.92	6037.86	2462.2	4714.17	5761.13	3541.15	1318.54	4050.87
Q 92989	Q92989	CLP1_HUMAN	CLP 1				1974.33			1537.99	5199.69	
Q 92990	Q92990	GLMN_HUMAN	GLMN				5175.84	6443.76	7199.26	3423.06	4303.02	3583.89
Q 93008	O00507;Q93008	USP9X_HUMAN	USP9X	2947.33	3340.85	3411.67	3730.11	4687.73	3587.94	3047.04	3631.93	2850.26
Q 93009	Q93009	UBP7_HUMAN	USP7	3227.73			3907.44	16843.4	11598.8	10569.7	5775.29	12608.9
Q 93034	Q93034	CUL5_HUMAN	CUL5				1538.18	1816.16	1879.27	1088.35		1596.67
Q 93050	Q93050	VPP1_HUMAN	ATP6V0A1					5967.19	5031.07	2948.97		2885.63
Q 93052	Q93052	LPP_HUMAN	LPP	1555.87		1266.86	2090.59	678.975	1291.25		984.053	
Q 93062	Q93062	RBPMS_HUMAN	RBPMS	5080.03	3419.17	4937.19	2909	5550.39	3444.67	915.442	1073.55	581.733
Q 93074	Q93074	MED12_HUMAN	MED12		955.568			1785.09	893.966	3511.88	2217.64	1738.84
Q 93100	Q93100	KPBB_HUMAN	PHKB					685.524	721.177	872.359		446.146
Q 969F2	Q969F2	NKD2_HUMAN	NKD2							1412.6		1593.92
Q 969F9	Q969F9	HPS3_HUMAN	HPS3						1072.41	1802.13	1696.77	1812.34
Q 969G5	Q969G5	CAVN3_HUMAN	CAVIN3				1748.12	1554.77	1481.51	3104.1		2592.5
Q 969G9	Q969G9	NKD1_HUMAN	NKD1							9064.73	10611.3	8389.62
Q 969H8	Q969H8	MYDGF_HUMAN	MYDGF	2468.3		3202.76	1917.57	4096.56	5144.19			1991.72
Q 969L2	Q969L2	MAL2_HUMAN	MAL2			5551.68	4943.36				5488.19	
Q 969M3	Q969M3	YIPF5_HUMAN	YIPF5	1801.55		2635.09	2761.4		1109.74	2158.12	1604.94	3012.62
Q 969S9	Q969S9	RRF2M_HUMAN	GFM2	2759.82		3592.9	3040.97	1453.71	2199.46	2049.61	522.018	1867.44
Q 969U7	Q969U7	PSMG2_HUMAN	PSMG2				492.375	1435.56	3126.16	1647.83		1815.21
Q 969V3	Q969V3	NCLN_HUMAN	NCLN	4530.2	3371.5	3755.07	2707.29	1867.12	1338.97	3110.6	3967.63	2445.98
Q 969X5	Q969X5	ERGI1_HUMAN	ERGIC1	5365.37	4856.04	9393.01	3719.04	2767.29	3331.37	3611.64	1482.59	3986.98
Q 969Z0	Q969Z0	FAKD4_HUMAN	TBRG4		7808.4	4117.64	7036.81	7744.33	6545.05	9948.15	12897.8	11399.7
Q 96A22	Q96A22	CK052_HUMAN	C11orf52						1438.46	6387.95		5627.09
Q 96A32	Q96A32	MYL11_HUMAN	MYL11									
Q 96A33	Q96A33	CCD47_HUMAN	CCDC47			6822.97	2830.55	3943.05	3046.71	6455.42	4070.7	6056.74
Q 96A49	Q96A49	SYAP1_HUMAN	SYAP1				589.491	1322.92	2270.28	2112.55	937.978	2455.84
Q 96A65	Q96A65	EXOC4_HUMAN	EXOC4	2816.13	4389.75	3363.12	3820.04	4870.71	3355.14	5079.68	4745	4126.28
Q 96AA3	Q96AA3	RFT1_HUMAN	RFT1			5594.99	3961.28			3931.09	7233.22	4294.93
Q 96AB3	Q96AB3	ISOC2_HUMAN	ISOC2				953.979	1736.44	765.086	1380.68	2390.76	2130.7
Q 96AC1	Q96AC1	FERM2_HUMAN	FERMT2			760.045	5212.82	713.485	773.883			
Q 96AE4	Q96AE4	FUBP1_HUMAN	FUBP1	6564.06		9150.89	6837.06	6152.14	8791.21	6849.47	8835.91	6819.74

Q96N66	Q96N66	MBOA7_HUMAN	MBOAT7		1960.2	981.151	764.019	2476.1	3051.11	1186.12		
Q96N67	Q96N67	DOCK7_HUMAN	DOCK7	60355.7	72207.9	56375.4	67437	47107.6	31063.5	51340.9	79200.4	61040.4
Q96NE9	Q96NE9	FRMD6_HUMAN	FRMD6	1967.73	2528.69	2062.95	806.001					519.66
Q96P11	Q96P11	NSUN5_HUMAN	NSUN5	4409.16	9153.29	4839.51	1940.12	6319.55	2042.49	5886.38	5612.75	5691.05
Q96P47	Q96P47	AGAP3_HUMAN	AGAP3					639.802		634.361	1065.94	612.912
Q96P48	Q96P48	ARAP1_HUMAN	ARAP1	1335.91		2973.94	3380.03	3914.17	6555.91	3285.41	2894.56	4876.38
Q96P63	Q96P63	SPB12_HUMAN	SERPINB12	32233.9	21268.5	54196.6	31013.6	4098.43	11224.7	9828.36	33833.6	23105.1
Q96P70	Q96P70	IPO9_HUMAN	IPO9	4642.3	6598.72	5559.91	6380.68	7699.23	6478.58	6659.07	6432.52	8731.11
Q96PC5	Q96PC5	MIA2_HUMAN	MIA2				1304.77	6089.17	4722.6	3954.6	1198.14	2111.22
Q96PK6	Q96PK6	RBM14_HUMAN	RBM14	22408.9	68154.8	19781.4	22370.8	87200.6	12561.3	11760.3	18535.6	15619.2
Q96PU5	Q96PU5	NED4L_HUMAN	NEDD4L				1113.58	490.253	958.133	1304.24	523.623	999.82
Q96PU8	Q96PU8	QKI_HUMAN	QKI	5165.01	3653.28	4957.68	6186.99	8120.1	5186.34	1761.51	1154.44	2197.6
Q96PV6	Q96PV6	LENG8_HUMAN	LENG8	6543.76	16012	3387.11	2074.52	11230.7	1600.07	1045.07		1213.2
Q96PY5	Q96PY5	FMNL2_HUMAN	FMNL2	4799.79	7821.87	8531.33	10743.7	44912.5	25226.4	45611.5	8577.51	39419.5
Q96Q11	Q96Q11	TRNT1_HUMAN	TRNT1				1590.76		1702.72	2627.37	1763.41	1612
Q96QA5	Q96QA5	GSDMA_HUMAN	GSDMA	15070.5	3092.39	11948.6	5601.01	37940.3	5409.42	3942.35	4133.1	5615.96
Q96QC0	Q96QC0	PP1RA_HUMAN	PPP1R10				449.87	1329.93	619.262	1371.75	270.409	886.532
Q96QD8	Q96QD8	S38A2_HUMAN	SLC38A2	2343.91	5900.28	4058.74	1052.51	3332.29	2234.52	2963.66		3715.71
Q96QK1	Q96QK1	VPS35_HUMAN	VPS35	3713.72	6844.26	4600.07	2444.71	3906.01	9814.73	3300.15	1967.6	3370.23
Q96QR8	Q9UJV8;Q96QR8	PURB_HUMAN	PURB	37600.1	78644.6	22043.7	29885.3	28451.6	13402	15865.2	17127.7	19892.8
Q96QU8	Q96QU8	XPO6_HUMAN	XPO6	4569.44	3674.72	4158.51	3111.56	1895.01	3466.85	5131.84	5190.7	4198.11
Q96RF0	Q96RF0	SNX18_HUMAN	SNX18	5601.62	3284.86	4363.06		5270.36	4186.28			
Q96RN5	Q96RN5	MED15_HUMAN	MED15					1825.43	1409.06	2158.06		2383.85
Q96RP9	Q96RP9	EFGM_HUMAN	GFM1			2390.01	2972.41	3766.91	3566.72	2659.12	2335.98	3217.95
Q96RQ3	Q96RQ3	MCCA_HUMAN	MCCC1	850938	803036	819732	591547	205670	159985	257288	719283	296687
Q96RQ9	Q96RQ9	OXLA_HUMAN	IL4I1					1583.1	1177.51			
Q96RS0	Q96RS0	TGS1_HUMAN	TGS1				2332.67			2401.82	4021.74	1942.02
Q96RT1	Q96RT1	ERBIN_HUMAN	ERBIN				4373.14	9956.78	7108.45	8446.89	2133.91	9935.66
Q96RT7	Q96RT7	GCP6_HUMAN	TUBGCP6			400.364	1343.83	2235.83	1405.23	1508.86	990.838	1286.95
Q96RT8	Q96RT8	GCP5_HUMAN	TUBGCP5					352.231	635.045	788.172		799.688
Q96S52	Q96S52	PIGS_HUMAN	PIGS	2837.92	2709	4088.78	1791.99	4784.98	3494.54	2972.74	1852.75	4390.84
Q96S55	Q96S55	WRIP1_HUMAN	WRNIP1		2996.57	1341.14	3882.96	4958.8	4989.78	6439.92	5778.71	6363.23
Q96S59	Q96S59	RANB9_HUMAN	RANBP9					1095.58	1993.37	1818.03		1623.42
Q96S97	Q96S97	MYADM_HUMAN	MYADM	3899.46	2276.56	6420.69	14251.5	2783.76	5047.31	10687.2	9108.2	13584.1
Q96SB4	Q96SB4	SRPK1_HUMAN	SRPK1				2276.84	2842.14	1796.01	2170	2978.01	2262.53
Q96SI9	Q96SI9	STRBP_HUMAN	STRBP		2692.61			6325	4033.7	5662.6	2626.9	
Q96SL4	Q96SL4	GPX7_HUMAN	GPX7									
Q96SN8	Q96SN8	CK5P2_HUMAN	CDK5RAP2	6521.95	7718.69	3012.38		3718.78	2724.16	1370.19		1761.25
Q96SQ9	Q96SQ9	CP2S1_HUMAN	CYP2S1				2363.15	4713.75	5077.98	7106.18	3622.87	5361.41
Q96ST3	Q96ST3	SIN3A_HUMAN	SIN3A			1070.53	970.522	1297.65	1190.98	1179.69		838.51
Q96SU4	Q96SU4	OSBL9_HUMAN	OSBPL9				1774.61	8669.28	11481.2	3544.79		
Q96SZ6	Q96SZ6	CK5P1_HUMAN	CDK5RAP1	2273.31			2775.41	8122.01	4131.87	7559.61	7152.53	7354.45
Q96T23	Q96T23	RSF1_HUMAN	RSF1				869.74	2665.49	1201.32	173.498		592.202
Q96T51	Q96T51	RUFY1_HUMAN	RUFY1				760.635	2227.07	1575.17	1705.44		
Q96T59	Q96T59	CDRTF_HUMAN	CDRT15	5742.8	7033.92	4046.5	4478.85	23724.7	11418.5	7908.38	2687.36	7174.82
Q96T60	Q96T60	PNKP_HUMAN	PNKP	5122.73	5166		6838.48	22852.5	3179.88	1797.85	762.659	847.297
Q96T76	Q96T76	MMS19_HUMAN	MMS19	2216.95	3388.2	2697.57	4284.24	6022.61	7254.11	2882.63	2937.9	3031.4
Q96T88	Q96T88	UHRF1_HUMAN	UHRF1				3350.56	2639.53	1756.21	2242.44	2816.67	2646.23
Q96TA1	Q96TA1	NIBA2_HUMAN	NIBAN2	23950.5	7246.5	10302.4	7425.07	9740.92	10037	4479.65	4937.08	4940.67
Q96TA2	Q96TA2	YMEL1_HUMAN	YME1L1					3833.95			5221.57	
Q96TC7	Q96TC7	RMD3_HUMAN	RMDN3							30173.1		41172.4
Q99081	Q99081	HTF4_HUMAN	TCF12					1798.77				
Q99436	Q99436	PSB7_HUMAN	PSMB7	2436.18		2194.95	1137.59	2840.01	8302.03	3145.69	533.658	3702.11
Q99439	Q99439	CNN2_HUMAN	CNN2	11095.4	9216.61	9429.83	15702.7	10457.2	5119.97	5438.44	8190.34	5423.41
Q99442	Q99442	SEC62_HUMAN	SEC62	2383.02			1399.17	1941.47	2420.71	3884.5	1339.74	3450.89
Q99459	Q99459	CDC5L_HUMAN	CDC5L			2082.44	3094	6291.36	1984.26	2767.82	1233.34	3514.61
Q99460	Q99460	PSMD1_HUMAN	PSMD1	7957.35	6863.23	10238	12725.4	13304.8	15873.6	11152.5	9609.83	11315.2
Q99470	Q99470	SDF2_HUMAN	SDF2					3457.88	3549.78	368.437		
Q99471	Q99471	PFD5_HUMAN	PFDN5						4733.93	790.339		1263.42
Q99497	Q99497	PARK7_HUMAN	PARK7	3787.62		4265.59	1900.19	1730.5	5901.65	2291.06	2254.32	2498.64
Q99536	Q99536	VAT1_HUMAN	VAT1	16412.5	7960.63	19267.3	15222.7	12324	31401.1	9510.8	7382.68	7366.9
Q99538	Q99538	LGMN_HUMAN	LGMN					1406.27	2594.89	822.163		2255.11
Q99543	Q99543	DNJC2_HUMAN	DNAJC2					3189.6	4254.33	2416.94		2556.42
Q99567	Q99567	NUP88_HUMAN	NUP88				3167.97	5647.43	5462.97	6824.45	1744.09	8387.65
Q99569	Q99569	PKP4_HUMAN	PKP4	1523.48				2859.54	1833.67	3810.48	738.186	3759.15
Q99570	Q99570	PI3R4_HUMAN	PIK3R4				5307.81	2863.13	3495.29	4824.77	4595.99	3926.52
Q99575	Q99575	POP1_HUMAN	POP1		2699.51		2988.67	5864.94	4774.67	4823.35	2876.79	4431.47
Q99584	Q99584	S10AD_HUMAN	S100A13	5483.38		3595.79	4470.73	2197.9	6587.04	3370.2	3796.76	2928.88
Q99615	Q99615	DNJC7_HUMAN	DNAJC7				2673.12	4894.55	4232.41	2568.64		3652.01
Q99623	Q99623	PHB2_HUMAN	PHB2	6875.7	5564.45	6017.43	4754.46	14133.8	9044.59	15466.7	8090.24	15174.4
Q99627	Q99627	CSN8_HUMAN	COPS8			4467.82	2339.65	1562.48	3026.48	2600.4		2068.79
Q99643	Q99643	C560_HUMAN	SDHC		1829.09					2454.22	1043.56	1431.9
Q99653	Q99653	CHP1_HUMAN	CHP1	5437.95	6392.7	6294.97	6696.72	6130.31	7598.35	7472.52	7198.33	6412.67
Q99661	Q99661	KIF2C_HUMAN	KIF2C				7628.47		3531.38	6197.02	5293.88	5224.56
Q99700	Q99700	ATX2_HUMAN	ATXN2	7967.14	6845.61		3766.18	12529.2	3366.87	2039.44	2904.77	2364.65
Q99707	Q99707	METH_HUMAN	MTR				6401.53		2224.08	2948.99	5603.25	2940.33
Q99714	Q99714	HCD2_HUMAN	HSD17B10	5105.43	1891.96	5905.61	3691.2		5245.53	6550.72	6835.79	12136.3
Q99729	Q99729	ROAA_HUMAN	HNRNPAB	7068.87	3988.91	1724.39	4768.82	11739	8564.16	5244.75	2717.64	5995.57
Q99733	Q99733	NP1L4_HUMAN	NAP1L4	6230.44		5682.02	5531.95	3124.74	7718.92	5459.89	2405.61	6770.94
Q99747	Q99747	SNAG_HUMAN	NAPG			1631.55	1701.97	844.737	919.84	1070.14		614.379
Q99755	Q99755	PI51A_HUMAN	PIP5K1A	4346.09	5667.06	3157.03	3047.05	8312.1	3870.82	1438.86		1450.91
Q99798	Q99798	ACON_HUMAN	ACO2			3741.34	3279.6	4803.78	6129.44	2924.44	2465.32	2885.39
Q99811	P54821;Q99811	PRRX2_HUMAN	PRRX2									
Q99816	Q99816	TS101_HUMAN	TSG101	8705		8139.34	13760.6	11173.5	14224.1	20377.3	6760.79	16788.8
Q99829	Q99829	CPNE1_HUMAN	CPNE1	5273.01	3510.87	3800.94	2057.97	6896.3	12866.4	6223.53	2822.68	4813.31
Q99832	Q99832	TCPH_HUMAN	CCT7	8811.4	7717.15	9165.48	15084.9	15746.2	21973.6	25790.2	12581.2	22865.5
Q99836	Q99836	MYD88_HUMAN	MYD88	7805.93	8683.95	7012.08	5575.1		6103.01	5324.53	4034.25	5821
Q99848	Q99848	EBP2_HUMAN	EBNA1BP2		1848.56		1028.56	2539.95	1108.33	1454		989.541
Q99856	Q99856	ARI3A_HUMAN	ARID3A				1050.97	2047.12	956.389			
Q99873	Q99873	ANM1_HUMAN	PRMT1	2587.57		2063.76	2566.49	3550.91	6449.63	3365.27	1747.29	4075.97
Q99958	Q99958	FOXC2_HUMAN	FOXC2									
Q99959	Q99959	PKP2_HUMAN	PKP2				2068.98	7287.06	3326.41	6571	1888.91	7264.18
Q9BPW8	Q9BPW8	NIPS1_HUMAN	NIPSNAP1		7271.59	8555.17	4617.79	5748.5	4745.66	3683.09		4618.1
Q9BPX3	Q9BPX3	CND3_HUMAN	NCAPG	2963.72	2539.23	2744.84	2170.69	5329.88	4679.89	3229.79	4529.5	3406.5
Q9BPX5	Q9BPX5	ARP5L_HUMAN	ARPC5L	6805.97		7353.54	7395.76	6828.63	2554.04	5754.74	7039.79	5319.8
Q9BPX6	Q9BPX6	MICU1_HUMAN	MICU1							1419.06		1537.35
Q9BQ13	Q9BQ13	KCD14_HUMAN	KCTD14							1767.76	2316.22	2200.99
Q9BQ39	Q9BQ39	DDX50_HUMAN	DDX50		2511.78	2049.95		3999.05	1195.33	3956.01	2630.17	4989.38
Q9BQ67	Q9BQ67	GRWD1_HUMAN	GRWD1				4746.99	4999.51	1913.84	1415.78	3400.16	2111.17
Q9BQ69	Q9BQ69	MACD1_HUMAN	MACROD1							3477.61		3821.09
Q9BQ70	Q9BQ70	TCF25_HUMAN	TCF25	1511.76	1409.57	1070.01				861.034		1170.38
Q9BQA1	Q9BQA1	MEP50_HUMAN	WDR77				1028.52		1633.02	952.864	2884.25	
Q9BQC3	Q9BQC3	DPH2_HUMAN	DPH2				1817.54	1841.06	2783.85	1829.77	882.981	1789.42
Q9BQE3	P68366;P68363;Q9BQE3;Q71U36;A6NHL2	TBA1C_HUMAN	TUBA1C	648857	578516	627288	799376	527215	476319	788648	937399	927101
Q9BQE5	Q9BQE5	APOL2_HUMAN	APOL2				7941.52	10187.8	7125.03	2713.52	2106.45	3270.19
Q9BQG0	Q9BQG0	MBB1A_HUMAN	MYBBP1A	1976.75	1763.82		753.205	3295.92	3227.56	4772.28	4779.22	3045.27
Q9BQP7	Q9BQP7	MGME1_HUMAN	MGME1	882.886	2643.56	3108.37	1919.25	2426.29	4835.12	2631.7	1627.7	3261.84
Q9BR76	Q9BR76	COR1B_HUMAN	CORO1B	7221.5	6873.05	7648.6	11685.1	51624.9	6930.92	12615.8	12330.5	6669.33
Q9BRA2	Q9BRA2	TXD17_HUMAN	TXNDC17	2097.28		1146	1532.29	799.566	2862.54	2491.28	1518.05	1904.44
Q9BRG1	Q9BRG1	VPS25_HUMAN	VPS25				1077.3	913.23	1604.05	1337.46	1013.98	1782.28
Q9BRJ6	Q9BRJ6	CG050_HUMAN	C7orf50	1943.78	2121.88	1895.34	1652.87	4292.69	4791.68	5017.79	3908.46	5913.06
Q9BRK4	Q9BRK4;O60513	LZTS2_HUMAN	LZTS2	6618.76	7639.16	6706.95	2252.49	3004.98	2261.88	3210.93		2315.05
Q9BRK5	Q9BRK5	CAB45_HUMAN	SDF4			3729.86	2924.02		1905.38	4141.44	5090.64	5462.53
Q9BRP4	Q9BRP4	PAAF1_HUMAN	PAAF1		2473.68		5563.8	5451.99	10457	7292.58	3557.52	7141.32
Q9BRP8	Q9BRP8	PYM1_HUMAN	PYM1	2357.47		2955.25	3496.67	3247.3	4851.21	4909.22	2885.22	3935.78
Q9BRQ6	Q9BRQ6	MIC25_HUMAN	CHCHD6	2650.65	4190.59	3307.84			459.984			597.14
Q9BRQ8	Q9BRQ8	FSP1_HUMAN	AIFM2				2479.53		1479.42	5738.75	2250.86	4697.93
Q9BRR6	Q9BRR6	ADPGK_HUMAN	ADPGK					1560.33	1071.02			436.493
Q9BRT6	Q9BRT6	LLPH_HUMAN	LLPH	3958.52	6372.67	3551.24	1791.4	14289.6	6592.53	2054.74	1532.02	1622.34
Q9BRX2	Q9BRX2	PELO_HUMAN	PELO		2648.58			1825.03	2042.01	1607.41	2310.91	2249.25
Q9BS26	Q9BS26	ERP44_HUMAN	ERP44					2288.31	3107.62	1740.62		1005.5
Q9BSD7	Q9BSD7	NTPCR_HUMAN	NTPCR	10319.3	7716.34	10698	10946.2	7404.81	5637.4	5017.13	6219.72	5686.12
Q9BSH4	Q9BSH4	TACO1_HUMAN	TACO1	983.421		1498.94	581.463	1864.12	2638.54	1145.52		785.565
Q9BSJ2	Q9BSJ2	GCP2_HUMAN	TUBGCP2	3068.52	2544.05	4006.06	4317.26	4398.79	4711.74	6429.12	4810.52	6860.55
Q9BSJ8	Q9BSJ8	ESYT1_HUMAN	ESYT1	4752.6		6491.51	10205.7	7884.5	8441.97	16064.8	10609.5	17378.2
Q9BST9	Q9BST9	RTKN_HUMAN	RTKN							2489.9	2906.23	3728.4
Q9BSV6	Q9BSV6	SEN34_HUMAN	TSEN34					1242.11	586.076	789.632		
Q9BT22	Q9BT22	ALG1_HUMAN	ALG1	7932.31	9849.89	11040.8	5576.08	4894.28	5945.16	10409.2	11564.4	12459.4
Q9BT40	Q9BT40	INP5K_HUMAN	INPP5K				1004.97	1406.08	1197.42	2343.57		2554.1
Q9BT78	Q9BT78	CSN4_HUMAN	COPS4	4457.15		7423.64	5814.56	3713.16	5016.62	5998.17	4170.56	5677.59
Q9BTD8	Q9BTD8	RBM42_HUMAN	RBM42	2108.78				2895.37	2255.43	1070.64		1495.48
Q9BTE3	Q9BTE3	MCMBP_HUMAN	MCMBP				962.859	1562.24	2290.23	1632.18	850.195	1985.07
Q9BTE6	Q9BTE6	AASD1_HUMAN	AARSD1						2177.55			756.741
Q9BTT0	Q9BTT0	AN32E_HUMAN	ANP32E			3807.84	3113.05		4205.43	2570.74	1374.78	2890.35
Q9BTT6	Q9BTT6	LRRC1_HUMAN	LRRC1					5151.18	3999.02	7309.9	1764.7	4143.79
Q9BTU6	Q9BTU6	P4K2A_HUMAN	PI4K2A	3412.43		3545.29	3461.15	5435.54	3983.37	5521.09	3180.27	4071.91
Q9BTV4	Q9BTV4	TMM43_HUMAN	TMEM43	3648.37	3749.98	4525.75	2726.25	2879.18	2766.03	3139.94	2210.63	4390.26
Q9BTW9	Q9BTW9	TBCD_HUMAN	TBCD	6216.7	4037.52	5169.83	8397.38	4445.15	6068	4929.48	11536	6352.03
Q9BTX1	Q9BTX1	NDC1_HUMAN	NDC1				1453.18	881.742	539.807	1595.94	962.749	1080.42
Q9BTY7	Q9BTY7	HGH1_HUMAN	HGH1			1759.35	4482.15			4840.76	4567.98	5205.88
Q9BUB7	Q9BUB7	TMM70_HUMAN	TMEM70						1516.69	2495.69	2881.9	2829.32
Q9BUF5	Q9BUF5;Q13509	TBB6_HUMAN	TUBB6	190529	101736	181636	195293	54230.8	53393.8	49350.1	55369.8	38265.2
Q9BUH6	Q9BUH6	PAXX_HUMAN	PAXX	8831.32	5804.32		8068.07	6177.66	11517.6	3872.37	3727.19	2078.73
Q9BUI4	Q9BUI4	RPC3_HUMAN	POLR3C	5574.36	5482.88		650.895	1195.05	739.598	604.956		467.12
Q9BUJ2	Q9BUJ2	HNRL1_HUMAN	HNRNPUL1	15471.7	21728.3	18266.9	5353.39	28665.7	10700.4	9749.53	7823.64	9583.48
Q9BUK6	Q9BUK6	MSTO1_HUMAN	MSTO1				1388.46	5058.85	1304.62	2085.37	965.27	2118.1
Q9BUL8	Q9BUL8	PDC10_HUMAN	PDCD10				2905.7	2133.59	2561.72	1858.32	1240.78	1144.58
Q9BUN8	Q9BUN8	DERL1_HUMAN	DERL1		5471.27	2677.43	3041.73			3936.5	1453.34	4092.59
Q9BUQ8	Q9BUQ8	DDX23_HUMAN	DDX23		2474.75		1466.48	2738.3	1769.85	7014.64	4172.95	6370
Q9BV40	Q9BV40	VAMP8_HUMAN	VAMP8				3880.06	3982.59	3894.51	7350.31		6342.94
Q9BV44	Q9BV44	THUM3_HUMAN	THUMPD3	4046.41			3943.6			1752.73	3057.36	
Q9BV68	Q9BV68	RN126_HUMAN	RNF126				3461.35			5011.88	4885.28	5878.3
Q9BV86	Q9BV86	NTM1A_HUMAN	NTMT1					2041.75	3520.59	1215.84	1097.89	
Q9BVC4	Q9BVC4	LST8_HUMAN	MLST8					644.558	1123.02	2293.92	1261.93	1611.44
Q9BVG4	Q9BVG4	PBDC1_HUMAN	PBDC1			14645	6520.53	5301.78	8193.28	3870.01	4816.87	4309.95
Q9BVG8	Q9BVG8	KIFC3_HUMAN	KIFC3		1433.32			2225.76	713.292	1931.13		2228.24
Q9BVI4	Q9BVI4	NOC4L_HUMAN	NOC4L	2159.2			1934.14	2696.18	2025.46	4247.61	2853.41	3978.41
Q9BVK6	Q9BVK6	TMED9_HUMAN	TMED9			9743.97	134818	10938.1	13020.4	20596.5	4108.51	17231.7
Q9BVL2	Q9BVL2	NUP58_HUMAN	NUP58				438.193	1317.81	2110.07	1016.27		
Q9BVP2	Q9BVP2	GNL3_HUMAN	GNL3	5805.41			4595.87		4814.48	7416.7	4292.15	7102.69
Q9BVQ7	Q9BVQ7	SPA5L_HUMAN	SPATA5L1	4845.45	4868.37	3022.77	5453.01	7676.89	3417.38	4766.49	3037.84	3729.32
Q9BVS4	Q9BVS4	RIOK2_HUMAN	RIOK2					2440.37	834.86			
Q9BW04	Q9BW04	SARG_HUMAN	SARG								779.511	528.99
Q9BW19	Q9BW19	KIFC1_HUMAN	KIFC1				2634.72	2404.34	1813.38	2017.56	2798.04	2021.13
Q9BW83	Q9BW83	IFT27_HUMAN	IFT27	3162.17	1396.16	3822.75				1651.82		1881.19
Q9BW92	Q9BW92	SYTM_HUMAN	TARS2	3702.14		3538.01	7833.44	5646.13	5034.06	8732	9096.81	9522.18
Q9BWF3	Q9BWF3;Q9BQ04	RBM4_HUMAN	RBM4	6431.29	6167.04	8212.92	4887.02			3527.93	6834.03	1861.55
Q9BWH6	Q9BWH6	RPAP1_HUMAN	RPAP1			3087.29	6178.46	4696.1	5644.8	5862.02	4247.2	5309.03
Q9BWJ5	Q9BWJ5	SF3B5_HUMAN	SF3B5				1480.64	797.148	668.462	689.635	1484.77	
Q9BWS9	Q9BWS9	CHID1_HUMAN	CHID1					968.533	3207.64	2677.49		
Q9BX10	Q9BX10	GTPB2_HUMAN	GTPBP2				2745.65	6374.2	3544.65	2312.67		3410.26
Q9BX40	Q9BX40	LS14B_HUMAN	LSM14B		3980.89	3543.03	2351.24		1918.09	3306.78		
Q9BX46	Q9H0Z9;Q9BX46	RBM24_HUMAN	RBM24				1449.69	5012.38	1701.34			
Q9BX68	Q9BX68	HINT2_HUMAN	HINT2			1481.25			2389.33	522.058		451.772
Q9BXB5	Q9BXB4;Q9BXB5	OSB10_HUMAN	OSBPL10		868.657		3338.78	3317.53	3872.35	3821.84	2206.4	2272.97
Q9BXJ9	Q9BXJ9	NAA15_HUMAN	NAA15	5671.35		3778.53	6287.67	5850.12	6571.14	4142.79		3479.28
Q9BXP2	Q9BXP2	S12A9_HUMAN	SLC12A9					5955.17	4073.37	1813.87		
Q9BXP5	Q9BXP5	SRRT_HUMAN	SRRT			1300.25		6921.17	5094.85	4840.93		3073.7
Q9BXS5	Q9BXS5	AP1M1_HUMAN	AP1M1	9452.84	16482.8	9862.65	8823.87	7972.85	10771.4	11983	6398.07	12004.7
Q9BXS6	Q9BXS6	NUSAP_HUMAN	NUSAP1							776.279	1503.49	856.332
Q9BXW6	Q9BXW6	OSBL1_HUMAN	OSBPL1A	10907	2958.37	13398.5	25714		11956.5	9864.76	24485.3	19842.8
Q9BXW7	Q9BXW7	HDHD5_HUMAN	HDHD5			7679.31	2000.61		2563.47	3675.12	1792.16	2592.22
Q9BXW9	Q9BXW9	FACD2_HUMAN	FANCD2				390.33	2287.95	931.943	1651.18	1170.29	2378.45
Q9BY44	Q9BY44	EIF2A_HUMAN	EIF2A	3827.58		3369.54	5974.98	3187.66	3684.34	2856.65	4080.63	2987
Q9BY77	Q9BY77	PDIP3_HUMAN	POLDIP3	8610.14	8361.62	11022.1	5728.02	7573.59	5960.98	3776.34	11982.2	6370.12
Q9BY89	Q9BY89	K1671_HUMAN	KIAA1671					694.612		813.331		
Q9BYD2	Q9BYD2	RM09_HUMAN	MRPL9	1572.36	1185.81	1664.94	1130.75	491.664	618.816	1544.24	1109.14	1644.46
Q9BYD3	Q9BYD3	RM04_HUMAN	MRPL4						2941.68	4332.67	2094.25	4020.52
Q9BYI3	Q9BYI3	HYCCI_HUMAN	HYCC1					6084.85	3320.37	1767.24		2687.24
Q9BYJ1	Q9BYJ1	LOXE3_HUMAN	ALOXE3	9896.78	12536.8	14728.5	4085.36		5506.36	4043.54	4338.17	6833.81
Q9BYK8	Q9BYK8	HELZ2_HUMAN	HELZ2					3901.17	1716			
Q9BYX7	Q6S8J3;P0CG38;Q9BYX7;P0CG39	ACTBM_HUMAN	POTEKP	22280.2	13642	28117.5	16099.4	24188.9	20609	26599	4392.29	31545.2
Q9BZE1	Q9BZE1	RM37_HUMAN	MRPL37	5066.76				3987.89	5233.41	6010.52	4428.04	4442.29
Q9BZE4	Q9BZE4	GTPB4_HUMAN	GTPBP4	8717.56		7190.19	6346.71		7238.54	6551.41	8044.48	5834.57
Q9BZF1	Q9BZF1	OSBL8_HUMAN	OSBPL8		2658.93	2028.3	1335.31	2999.18	3278.22	6371.59	2138.36	5948.94
Q9BZF9	Q9BZF9	UACA_HUMAN	UACA	3246.33	11008	8564.3	46350	101091	53793.3	8111.59	6194.36	6538.96
Q9BZH6	Q9BZH6	WDR11_HUMAN	WDR11					1645.23	1293.5	1237.87		1673.48
Q9BZI7	Q9BZI7	REN3B_HUMAN	UPF3B					928.003	571.784	1050.12	790.422	
Q9BZJ0	Q9BZJ0	CRNL1_HUMAN	CRNKL1				3318.79				5154.9	
Q9BZL4	Q9BZL4	PP12C_HUMAN	PPP1R12C				778.882	3164.04	1535.59			
Q9BZL6	Q9BZL6	KPCD2_HUMAN	PRKD2	976.106	1260.71	1421.4	2592.43	3644.98	4341.78	5508.9	1952.69	4892.96
Q9BZM5	Q6H3X3;Q9BZM5;Q5VY80	ULBP2_HUMAN	ULBP2					2360.76		5519.27	5957.62	5116.49
Q9BZQ8	Q9BZQ8	NIBA1_HUMAN	NIBAN1	5006.28		6579.35	3342.22	1615.48	2071.96			
Q9BZX2	Q9BZX2	UCK2_HUMAN	UCK2		4771.48	2813.51	9137.19	6522.9	9705.06	13462.8	14115.9	14238.2
Q9BZZ5	Q9BZZ5	API5_HUMAN	API5	6803.1		5945.01	6390.39	2916.2	6961.35	6813.39	4667.48	7867.49
Q9C005	Q9C005	DPY30_HUMAN	DPY30				8525.03	7951.42	3951.27	4521.08	6993.55	4599.4
Q9C037	Q9C037	TRIM4_HUMAN	TRIM4	3205.53	3755.42	3779.53	1895.53			2608.55	2424.72	2188.81
Q9C075	Q9C075	K1C23_HUMAN	KRT23			7593.62	4805.82		2464.37	14980.2	7233.85	15915.7
Q9C0B5	Q9C0B5	ZDHC5_HUMAN	ZDHHC5			2276.95	3385.86	5737.44	4147.1	13184.5	4469.6	11538.3
Q9C0B7	Q9C0B7	TNG6_HUMAN	TANGO6				3727.3	3092.01	2251.79	4644.88	8887.48	3541.03
Q9C0C2	Q9C0C2	TB182_HUMAN	TNKS1BP1	15098.6	23181.4	12913	7753.52	11450.8	6783.78	4804.21	6377.55	6376.43
Q9C0C9	Q9C0C9	UBE2O_HUMAN	UBE2O				3826.45	4182.76	3914.32	1612.76		3459.38
Q9C0E2	Q9C0E2	XPO4_HUMAN	XPO4				3133.66	3495.14		2484.32	3010.76	2641.98
Q9GZL7	Q9GZL7	WDR12_HUMAN	WDR12						1296.14	1252.59		1500.26
Q9GZS3	Q9GZS3	SKI8_HUMAN	SKIC8				5334.35	2886	5380.93	6930.14	5027.39	6599.75
Q9GZT3	Q9GZT3	SLIRP_HUMAN	SLIRP	6098.19	5020.98	5109.44	8108.28	6574.48	6486.8	13591.8	17983.5	9265.68
Q9GZT8	Q9GZT8	NIF3L_HUMAN	NIF3L1					2398.54	3746.07	2021.15		1555.98
Q9GZV6	Q9GZV6	NTAL_HUMAN	LAT2				1293.3	2031.01	1360.33	4111.5	1077.82	5449.21
Q9GZZ1	Q9GZZ1	NAA50_HUMAN	NAA50	5142.55		5858.64	1821.23	3339.94	7063.22	3693.14		2438.27
Q9GZZ8	Q9GZZ8	LACRT_HUMAN	LACRT								13812.4	
Q9GZZ9	Q9GZZ9	UBA5_HUMAN	UBA5				6619.37	9583.54	3283.22	4556.89	6864.67	4645.45
Q9H019	Q9H019	MFR1L_HUMAN	MTFR1L	8604.44	6065.28	6108.55	2938.89	3093.73	2881.7	2764.97	1620.98	3281.15
Q9H0A0	Q9H0A0	NAT10_HUMAN	NAT10	1480.55		1924.16	1064.45	530.406	642.363	2969.49	3068.59	3509.32
Q9H0D6	Q9H0D6	XRN2_HUMAN	XRN2	25140.1	38351	7767.3	8652.65	15682.1	8508.84	9957.59	10654.2	10658.2
Q9H0E2	Q9H0E2	TOLIP_HUMAN	TOLLIP					3269.52	3321.66	3712.64		1501.17
Q9H0H5	Q9H0H5	RGAP1_HUMAN	RACGAP1		2946.15	3252.62		7337.93	3731.31	1278.47	1307.43	1714.6
Q9H0K1	Q9H0K1	SIK2_HUMAN	SIK2					1413.17	1054.84	910.645		
Q9H0P0	Q9H0P0	5NT3A_HUMAN	NT5C3A					2785.93	664.438	4476.4		2070.63
Q9H0R4	Q9H0R4	HDHD2_HUMAN	HDHD2			392.491			1205.15			
Q9H0S4	Q9H0S4	DDX47_HUMAN	DDX47	4526.97	3703.27	2256.1	4197.41	3864.5	4315.95	6161.74	6861.75	4220.66
Q9H0U3	Q9H0U3	MAGT1_HUMAN	MAGT1				962.138		1560.43	6408.69		3071.16
Q9H0U4	Q92928;Q9H0U4	RAB1B_HUMAN	RAB1B	36531.4	30203.9	38812	29494.2	91133.4	38770.7	71804.9	26111.4	63375
Q9H0W8	Q9H0W8	SMG9_HUMAN	SMG9	3190.85		2490.72	3760.85	3035.34	3185.19	2300.19	3580.67	2500.59
Q9H0W9	Q9H0W9	CK054_HUMAN	C11orf54			3597.56	2320.98	2550.44	4255.68	3010.76	2287.73	3944.56
Q9H147	Q9H147	TDIF1_HUMAN	DNTTIP1				806.662	1358.42	1098.8	709.321		
Q9H1A4	Q9H1A4	APC1_HUMAN	ANAPC1	1268.19			1008.95	1462.91	1097.04	1741.89	1074.83	2016.01
Q9H1D9	Q9H1D9	RPC6_HUMAN	POLR3F				2245.07					
Q9H1E1	Q9H1E1	RNAS7_HUMAN	RNASE7	15374.2	18664	27048.8	8460.55	4411.12	9646.46	6838.84	7817.8	13873
Q9H1E3	Q9H1E3	NUCKS_HUMAN	NUCKS1				3527.73				3275.73	
Q9H1K4;Q9H936	Q9H1K4;Q9H936	GHC1_HUMAN;GHC2_HUMAN	SLC25A18;SLC25A22				1989.02			1369.06	3988.79	
Q9H1Y0	Q9H1Y0	ATG5_HUMAN	ATG5				986.386		1678.9	1229	712.296	1138.94
Q9H223	Q9NZN3;Q9H4M9;Q9H223	EHD4_HUMAN	EHD4	8000.38	5989.72	8916.1	15758.3	4984.1	5824.93	6000.78	10840	6305.69
Q9H267	Q9H267	VP33B_HUMAN	VPS33B					605.024	539.629	422.425		
Q9H269	Q9H269	VPS16_HUMAN	VPS16				2811.62	1831.6	1427.43	1174.15	1724.1	926.097
Q9H299	Q9H299	SH3L3_HUMAN	SH3BGRL3				1976.79	2781.01	4309.68	2074.79	783.828	1498.06
Q9H2C0	Q9H2C0	GAN_HUMAN	GAN	19101.4	11419.2	20952.8	4726.74	9728.88	5477.11	33660.6	10862.8	41923.7
Q9H2D6	Q9H2D6	TARA_HUMAN	TRIOBP	8179.8	9035.62	6183.26	4429.66	5477.87	3758.81	1471.13		1282.64
Q9H2G2	Q9H2G2	SLK_HUMAN	SLK	101416	69084.1	257474	74346.2	27947.8	76169.6	54947.7	76982.5	103044
Q9H2M9	Q9H2M9	RBGPR_HUMAN	RAB3GAP2	1549.02	958.261	620.377	2295.31	2129.03	2630.45	2381.84	1667.3	2354.01
Q9H2P0	Q9H2P0	ADNP_HUMAN	ADNP				967.856	1001.77	1170.98	2051.8	1610.59	1920.49
Q9H2U1	Q9H2U1	DHX36_HUMAN	DHX36	2703.11	2992.87		2862.36	13420.8	4183.59	1977.36	2020.52	1930.4
Q9H2U2	Q9H2U2	IPYR2_HUMAN	PPA2			11363.2			3677.42	7130.35	4158.76	5094.98
Q9H2V7	Q9H2V7	SPNS1_HUMAN	SPNS1				2390.12				2114.74	
Q9H2X9	Q9H2X9	S12A5_HUMAN	SLC12A5									
Q9H307	Q9H307	PININ_HUMAN	PNN				2111.61	8172.96		2174.42	2006.84	
Q9H329	Q9H329	E41LB_HUMAN	EPB41L4B							2839.65		2050.1
Q9H3G5	Q9H3G5	CPVL_HUMAN	CPVL				6176.88	3321.93	3149.19			
Q9H3M7	Q9H3M7	TXNIP_HUMAN	TXNIP	5464.24	8787.91	5210.3		4255.62	1706.49	36035.5	10169	22602.6
Q9H3N1	Q9H3N1	TMX1_HUMAN	TMX1	4403.98	4726.5	5829.77	5623.89	7386.79	12470.6	6308.49	5583.91	5266.83
Q9H3P2	Q9H3P2	NELFA_HUMAN	NELFA	3671.21	1512.46	2038.28	2055.21	2626.19	2315.79	2933.02	1608.4	3262.31
Q9H3P7	Q9H3P7	GCP60_HUMAN	ACBD3				2541.27				1289.21	
Q9H3R2	Q9H3R2	MUC13_HUMAN	MUC13							8716.46	5916.74	7625.91
Q9H3U1	Q9H3U1	UN45A_HUMAN	UNC45A	10572.7	8125.41	10014.1	18395.4	15284.8	8009.4	11614.9	13447.4	10703.7
Q9H425	Q9H425	CA198_HUMAN	C1orf198				1135.92	2095.9	1676.21	2652.35		2053.22
Q9H444	Q9H444	CHM4B_HUMAN	CHMP4B	2673.08	2143.51		3075.93		1646.06	3101.79	3036.08	
Q9H488	Q9H488	OFUT1_HUMAN	POFUT1				2743.03	1570.03	2562.11			
Q9H4A3	Q9H4A3	WNK1_HUMAN	WNK1				1481.25	1185.83	1225.29	2673.55	1871.1	3389.08
Q9H4A4	Q9H4A4	AMPB_HUMAN	RNPEP	4176.25		4052.3	3311.54		1879.93	1603.73		3028.18
Q9H4B6	Q9H4B6	SAV1_HUMAN	SAV1			4753.12	1466.06	2729.59	2746.3	4007.47		4787.29
Q9H4G4	Q9H4G4	GAPR1_HUMAN	GLIPR2				6620.51	4610.6	4440.76	940.161	3229.28	
Q9H4H8	Q9H4H8	FA83D_HUMAN	FAM83D				1612.81	3323.42	2265.29	2440.84		2611.74
Q9H4L5	Q9H4L5	OSBL3_HUMAN	OSBPL3				1650.98	2298.72	1346.9	3946.21	2029.49	3893.46
Q9H4L7	Q9H4L7	SMRCD_HUMAN	SMARCAD1		1117.94		1549.69	3060.01	2088.41	4159.97	1972.71	3105.31
Q9H4M9	Q9NZN3;Q9H4M9	EHD1_HUMAN	EHD1	6349.1	6980.28	7946.79	5398.72	6084.91	6002.35	4266.37	4175.26	4156.6
Q9H4P4	Q9H4P4	RNF41_HUMAN	RNF41		5469.38		13163.7	8878.24	8493.53	5892.12	7257.99	7439.61
Q9H553	Q9H553	ALG2_HUMAN	ALG2	11770.6	8652.95	9196.89	5973.27	5578.15	4863.32	5890.93	5679.47	6844.5
Q9H583	Q9H583	HEAT1_HUMAN	HEATR1					5148.4	1945.06	2680.78	1494.98	4956.86
Q9H5Q4	Q9H5Q4	TFB2M_HUMAN	TFB2M	5625.74	4065.16	4515.6	9071.61	17747.8	10909.6	9427.78	10646	9465.41
Q9H5V8	Q9H5V8	CDCP1_HUMAN	CDCP1							21480.4	9179.53	19872.4
Q9H5Z1	Q9H5Z1	DHX35_HUMAN	DHX35	3329.2	1980.68	2679.06	3471.01			1345.13	1467.65	1706.28
Q9H694	Q9H694	BICC1_HUMAN	BICC1									
Q9H6R7	Q9H6R7	WDCP_HUMAN	WDCP				1901.09		873.69	742.183	2402.67	1203.78
Q9H6S3	Q9H6S3	ES8L2_HUMAN	EPS8L2			5564.56	7377.34		3110.38	4043.93	6321.92	4543.36
Q9H6T3	Q9H6T3	RPAP3_HUMAN	RPAP3				2757.99		3618.36	2989.02		5045.69
Q9H6V9	Q9H6V9	LDAH_HUMAN	LDAH				1210.19	4711.81	4137.7	5116.31		4508.63
Q9H6W3	Q9H6W3	RIOX1_HUMAN	RIOX1					3197.94		2081.77		1761.17
Q9H773	Q9H773	DCTP1_HUMAN	DCTPP1						591.619			
Q9H7B4	Q9H7B4	SMYD3_HUMAN	SMYD3		2386.69	2017.24	1735.69		1882.97	2103.76	2121.95	1655.9
Q9H7D7	Q9H7D7	WDR26_HUMAN	WDR26	5858.72	12696.8	9091.15	4511.14	4584.25	6869.71	4537.27		4643.67
Q9H7E9	Q9H7E9	CH033_HUMAN	C8orf33				1951.38			745.133	2827.87	779.057
Q9H814	Q9H814	PHAX_HUMAN	PHAX					6201.73	3276.49	1079.21		
Q9H845	Q9H845	ACAD9_HUMAN	ACAD9	4858.87	5881.37	5750.54	13005.2		2289.57	4626.26	3677.63	5103.68
Q9H857	Q9H857	NT5D2_HUMAN	NT5DC2	7576.27	5898.32	4461.61	2629.37	6238.37	3816.84	8635.29	6148.55	4799.61
Q9H8H0	Q9H8H0	NOL11_HUMAN	NOL11				800.406	1590.54	1640.32	1770.56	784.9	
Q9H8H3	Q9H8H3	TMT1A_HUMAN	TMT1A							2957.39	4628.61	4630.01
Q9H8N7	Q9H8N7	ZN395_HUMAN	ZNF395	1580.51			1320.25				1633.97	998.416
Q9H8V3	Q9H8V3	ECT2_HUMAN	ECT2					1115.29		1177.51		706.023
Q9H8Y8	Q9H8Y8	GORS2_HUMAN	GORASP2	2441.19	1446.31	2071.17	1946.57	1896.71	2085.84	4190.08	1189.14	2827.86
Q9H900	Q9H900	ZWILC_HUMAN	ZWILCH						1366.06	2199.67	3832.2	
Q9H910	Q9H910	JUPI2_HUMAN	JPT2	3290.14		2302.41	4267.35	3289.64	5408.38	3227.62	3826.4	4647.42
Q9H967	Q9H967	WDR76_HUMAN	WDR76	6337.43	10786.1		2094.71				3013.07	
Q9H974	Q9H974	QTRT2_HUMAN	QTRT2	4181.28	4974.17	5011.07	2117.64	3963.87	2245.29	3281.11	2136.23	3125.77
Q9H981	Q9H981	ARP8_HUMAN	ACTR8				760.651	1085.52	1171.26	860.358	521.624	880.558
Q9H9A5	Q9H9A5	CNO10_HUMAN	CNOT10					1518.83	800.984	1396.23		908.8
Q9H9A6	Q9H9A6	LRC40_HUMAN	LRRC40	7876.04	7434.59	6946.79	4667.74	5438.84	2708.87	5028.32	3399.37	5829.04
Q9H9A7	Q9H9A7	RMI1_HUMAN	RMI1	10283.8	28595.9		4659.89	8034.69		1369	3130.86	
Q9H9E3	Q9H9E3	COG4_HUMAN	COG4			7303.67	1944.68	2143.84	1684.84	2470.19	1566.22	3422.59
Q9H9G7	Q9H9G7	AGO3_HUMAN	AGO3					11474.7	4755.7	4587.58		4774.6
Q9H9H4	Q9H9H4	VP37B_HUMAN	VPS37B			1584.37	2084.46	3043.5	2989.72	2952.68	843.413	2084.8
Q9H9P8	Q9H9P8	L2HDH_HUMAN	L2HGDH			1880.58	1809.3	1659.98	1132.53	7698.32	6835.68	8513.52
Q9H9T3	Q9H9T3	ELP3_HUMAN	ELP3		3064.31	3974.77	8517.09	10718.3	12734.1	11419.7	8504.69	11950.7
Q9H9Z2	Q9H9Z2	LN28A_HUMAN	LIN28A				8896.11	14927.1	13202.6			
Q9HA77	Q9HA77	SYCM_HUMAN	CARS2							3900.05	2016.39	4026.52
Q9HAC8	Q8WUN7;Q9HAC8	UBTD1_HUMAN	UBTD1				3204.65	8706.03	7097.63	11098.8	2148.1	9230.13
Q9HAS0	Q9HAS0	NJMU_HUMAN	C17orf75				1043.31		1246.64	1312.44		1306.91
Q9HAU0	Q9HAU0	PKHA5_HUMAN	PLEKHA5	11246.4	11017.6	10904.8	9214.18	8809.27	7767.68	2938.05	3326.33	3551.72
Q9HAV4	Q9HAV4	XPO5_HUMAN	XPO5				5774.45	4180.2	4345.16	7410.36	6985.26	9801
Q9HAV7	Q9HAV7	GRPE1_HUMAN	GRPEL1						3670.73	3347.31		2539.03
Q9HB07	Q9HB07	MYG1_HUMAN	MYG1				2990.9	2114.34	7179.42	4223.43		2734.79
Q9HB71	Q9HB71	CYBP_HUMAN	CACYBP	2229.3		3479.43	2346.85	2758.32	3561.74	3723.5	1754.53	3665.28
Q9HB90;Q9NQL2	Q9NQL2;Q9HB90	RRAGC_HUMAN;RRAGD_HUMAN	RRAGC;RRAGD				2239.66		2252.93	4585.02		4999.65
Q9HBH1	Q9HBH1	DEFM_HUMAN	PDF							2299.56	1955.18	2812.22
Q9HC07	Q9HC07	TM165_HUMAN	TMEM165			2682.68	2252.48		876.833	2790.09	2210.47	2654.19
Q9HC35	Q9HC35	EMAL4_HUMAN	EML4	2179.85		2382.85	4879.08	3744.05	5398.95	3904.9	2936.25	3197.4
Q9HC36	Q9HC36	MRM3_HUMAN	MRM3					850.29	474.341	1527.28	930.156	1320.92
Q9HC38	Q9HC38	GLOD4_HUMAN	GLOD4			18154.9	21702.5		2402.52	6303.85		3724.35
Q9HC84	Q9HC84	MUC5B_HUMAN	MUC5B			3012.7					3511.91	
Q9HCC0	Q9HCC0	MCCB_HUMAN	MCCC2	967844	886827	1.07395E+06	496079	159907	156055	413533	758240	446840
Q9HCE1	Q9HCE1	MOV10_HUMAN	MOV10	2243.67	3181.53	2154.96		4238.92	1192.88	1739.7	1342.64	2325.01
Q9HCE6	Q9HCE6	ARGAL_HUMAN	ARHGEF10L					1970.74	2062.77			3005.57
Q9HCE7	Q9HCE7	SMUF1_HUMAN	SMURF1				7822.25				5619.52	3569.13
Q9HCH0	Q9HCH0	NCK5L_HUMAN	NCKAP5L						541.46			
[illegible]	[illegible]	[illegible]	[illegible]	[illegible]	[illegible]	[illegible]	[illegible]	[illegible]	[illegible]	[illegible]	[illegible]	[illegible]

Q 9HD20	Q9HD20	AT131_HUMAN	ATP13A1	2054.73	1435.11	1700.62	1757	6682.07	3176.89	2333.38	1183.11	2479.61
Q 9HD45	Q9HD45	TM9S3_HUMAN	TM9SF3	6563.56	6138.38	6536.66	5705.13	7856.39	8540.88	9297.41	7458.73	8661.61
Q 9HD67	Q9HD67	MYO10_HUMAN	MYO10					7821.82	8217.7	6696.97	12637.1	11306.7
Q 9HDC9	Q9HDC9	APMAP_HUMAN	APMAP	5758.58	3396.29	4170.23	3592.3	6118.91	6930.99	10442.5	9071.09	8617.44
Q 9NNW5	Q9NNW5	WDR6_HUMAN	WDR6				2602.63	7417.2	5514.84	1163.46		5770
Q 9NP55	Q9NP55	BPIA1_HUMAN	BPIFA1	7233.32	5720.18	12879.5	3541.79	3383.2	3606.42	4369.25	9875.51	2713.2
Q 9NP61	Q9NP61	ARFG3_HUMAN	ARFGAP3					2379.1	2598.66	490.008		
Q 9NP66	Q9NP66	HM20A_HUMAN	HMG20A	3387.89				1781.42	1591.08			
Q 9NP72	Q9NP72	RAB18_HUMAN	RAB18									
Q 9NP79	Q9NP79	VTA1_HUMAN	VTA1	3137.13		2943.85	4776.6	2783.66	3251.11	2186.3		2960.26
Q 9NP81	Q9NP81	SYSM_HUMAN	SARS2	2954.06		3530.53	2888.63	3509.23	3164.05	2476.8	1938.31	3232.17
Q 9NP97	Q9NP97;Q8TF09	DLRB1_HUMAN	DYNLRB1	2326.85		954.049	1390.23	3209.65	3408.08	1714.56	1538.73	2288.98
Q 9NPA0	Q9NPA0	EMC7_HUMAN	EMC7		1423.69		1061.97		2712.7	2683.29		3718.03
Q 9NPA3	Q9NPA3	M1IP1_HUMAN	MID1IP1			3843.58	3310.54	1880.76	2343.62	2333.11	3448.11	2272.01
Q 9NPA8	Q9NPA8	ENY2_HUMAN	ENY2					5932.53	1789	1285.66		
Q 9NPD3	Q9NPD3	EXOS4_HUMAN	EXOSC4	2778.33	3123.46	3188.16	1918.63	2373.85	5318.6	3678.43	1252.57	3936.82
Q 9NPH2	Q9NPH2	INO1_HUMAN	ISYNA1					2665.36	5797.43			
Q 9NPI6	Q9NPI6	DCP1A_HUMAN	DCP1A				648.699	2950.82	2596.94	723.203	594.466	557.03
Q 9NPJ3	Q9NPJ3	ACO13_HUMAN	ACOT13					1230	2459.84	604.58	807.832	862.322
Q 9NPQ 8	Q9NPQ8;Q9NVN3	RIC8A_HUMAN	RIC8A				5831.46	5683.09	7737.25	6174.99	7813.85	6172.8
Q 9NQ 84	Q9NQ84	GPC5C_HUMAN	GPRC5C				4474.02	8528.82	7145.59	4543.31		4228.03
Q 9NQ A3	C4AMC7;Q6VEQ5;A8K0Z3;A8MWX3;Q9NQA3	WASH6_HUMAN	WASH6P	2632.02	2811.43	1755.29	2821.75	1793.08	3878.46	2652.38	1958.44	2845.92
Q 9NQ B0	Q9NQB0	TF7L2_HUMAN	TCF7L2				1968.41					
Q 9NQ C3	Q9NQC3	RTN4_HUMAN	RTN4	35297.6	18158.4	38939.2	24543.6	21754.5	25799	19323.8	17848.9	18478.9
Q 9NQ G5	Q9NQG5	RPR1B_HUMAN	RPRD1B				3587.08	3661.06	7355.79	1192.24		2209.86
Q 9NQ P4	Q9NQP4	PFD4_HUMAN	PFDN4	4277.08		2942.85	2428.52		2774.51	1962.84	1982.07	1833.62
Q 9NQ S1	Q9NQS1	AVEN_HUMAN	AVEN		623.337			1140.23	373.131	324.622		
Q 9NQ T4	Q9NQT4	EXOS5_HUMAN	EXOSC5					1111.28	1954.57	2458.79		1389.37
Q 9NQ T5	Q9NQT5	EXOS3_HUMAN	EXOSC3					696.615	824.249	704.344		561.394
Q 9NQ W6	Q9NQW6	ANLN_HUMAN	ANLN				668.746	671.263	704.738	543.023	304.564	289.142
Q 9NQ W7	Q9NQW7	XPP1_HUMAN	XPNPEP1	4326.16		3321.15	3826.43	5497.49	8941.76	4004.72	2258.27	4185.49
Q 9NQ X3	Q9NQX3	GEPH_HUMAN	GPHN					13100.7	5235.21	3180.04		
Q 9NQ X4	Q9NQX4	MYO5C_HUMAN	MYO5C				5348.14	11328.7	7000.99	25137.9	12896.1	24294.6
Q 9NQ Z2	Q9NQZ2	SAS10_HUMAN	UTP3							9204.52	17409.2	
Q 9NR09	Q9NR09	BIRC6_HUMAN	BIRC6				1782.35		674.17	2672.54		2117.74
Q 9NR12	Q9NR12	PDLI7_HUMAN	PDLIM7	17233.1	14910.1	15253.1	26129.5	8773.14	10023.8	6019.53	8649.08	6793.04
Q 9NR30	Q9NR30	DDX21_HUMAN	DDX21	2724.18	3236.84	5552.5	7389.74	12225.9	9934.51	17963.8	18620.1	19278
Q 9NR31	Q9NR31	SAR1A_HUMAN	SAR1A	11802.2	5801.25	16985.7	19800.1		2495.11	9564.64	14673	10958.4
Q 9NR45	Q9NR45	SIAS_HUMAN	NANS		3099.78	5476.27	3017.74	3471.79	6368.3	3546.38		2847.16
Q 9NR50	Q9NR50	EI2BG_HUMAN	EIF2B3	8759.82	8795.65	7641.55	11489.2	6559.43	7647.53	10457.8	10559.5	11572.1
Q 9NR56	Q9NUK0;Q5VZF2;Q9NR56	MBNL1_HUMAN	MBNL1			1651.09	2125.62	3617.81	2710.42	1679.68	1681.23	2473.51
Q 9NRA8	Q9NRA8	4ET_HUMAN	EIF4ENIF1				617.647	2836.63	1136.38	434.436		
Q 9NRF8	Q9NRF8	PYRG2_HUMAN	CTPS2			2494.43	2835.01	2141.41	4305.48	4413.73	5389.72	5513.24
Q 9NRG0	Q9NRG0	CHRC1_HUMAN	CHRAC1				934.634	584.925	604.474	444.749	1031.83	
Q 9NRG9	Q9NRG9	AAAS_HUMAN	AAAS		4345.01	2173.58	8261.62	9581.16	2813.15	5530.37	6293.55	7788.89
Q 9NRN7	Q9NRN7	ADPPT_HUMAN	AASDHPPT				4609.1	2513.22	8398.91	2622.07		3357.6
Q 9NRP0	Q9NRP0	OSTC_HUMAN	OSTC	4035.96	3492.28	4407.1	2623.24	3970.35	3367.74	5753.77	2977.3	3881.96
Q 9NRR4	Q9NRR4	RNC_HUMAN	DROSHA		2592.6			1473.95		815.675		376.718
Q 9NRR6	Q9NRR6	INP5E_HUMAN	INPP5E									
Q 9NRR8	Q9NRR8	C42S1_HUMAN	CDC42SE1					1654.48	1002.99	2680.16		2638.43
Q 9NRW3	Q9NRW3	ABC3C_HUMAN	APOBEC3C	3405.59	7504.44	2279.96	1948.09	3159.06		2138.91	1756.2	1208.58
[illegible]	[illegible]	[illegible]	[illegible]	[illegible]	[illegible]	[illegible]	[illegible]	[illegible]	[illegible]	[illegible]	[illegible]	[illegible]

Q9Y222	Q9Y222	DMTF1_HUMAN	DMTF1		1309.44			1070.89				
Q9Y224	Q9Y224	RTRAF_HUMAN	RTRAF	5469.86	3679.63	1239.67	3113.26	14326.6	5848.33	4610.03	1865.82	4716.25
Q9Y230	Q9Y230	RUVB2_HUMAN	RUVBL2	16288.8	13670.2	17158	23733.7	22685	23214.3	13179.2	11169.2	14372.2
Q9Y237	Q9Y237	PIN4_HUMAN	PIN4					310.058	970.04	395.387		271.701
Q9Y261	Q9Y261	FOXA2_HUMAN	FOXA2								2629.49	
Q9Y262	Q9Y262	EIF3L_HUMAN	EIF3L	5455.75	2287.11	4529.49	9156.32	5336.94	6884.66	3541.59	4648.64	4877.93
Q9Y263	Q9Y263	PLAP_HUMAN	PLAA	6548.79	5481.55	4576.76	5852.96	1593.51	2657.88	1911.61	4720.68	2374.49
Q9Y265	Q9Y265	RUVB1_HUMAN	RUVBL1	32692.8	28722.6	30879.4	48235.7	48315.9	43874.3	33733.8	29845.8	28628.9
Q9Y266	Q9Y266	NUDC_HUMAN	NUDC	7890.18	4271.83	6809.43	7787.57	6772.12	14873.7	16970.7	11920	16883
Q9Y277	Q9Y277	VDAC3_HUMAN	VDAC3	13357.3	20480.8	14232.3	1575.51	8152.79	4917.3	5703.93	5122.32	6633.02
Q9Y281	Q9Y281	COF2_HUMAN	CFL2			12783.8	1870.14	15485.9	9330.46			
Q9Y285	Q9Y285	SYFA_HUMAN	FARSA			4036.44	3860.03	3610.18	3174.58	3889.09	3086.78	4002.71
Q9Y289	Q9Y289	SC5A6_HUMAN	SLC5A6	1807.63			2694.53		3749.9	5840.6	3833.34	4784.4
Q9Y295	Q9Y295	DRG1_HUMAN	DRG1	4363.58	2616.83	3348.3	2269.73	4584.42	6003.38	5218.39	1208.39	5932.25
Q9Y2A7	Q9Y2A7	NCKP1_HUMAN	NCKAP1	7220.96	7393.52	12281	5828.48	7048.1	7855.77	5088.61	3290.53	5478.95
Q9Y2B0	Q9Y2B0	CNPY2_HUMAN	CNPY2			893.39	4009.07		1178.43		3362.59	1135.65
Q9Y2D4	Q9Y2D4	EXC6B_HUMAN	EXOC6B	2851.02	2898.53	3395.96	1839.27	3218.15	3714.85	1603.41	1229.32	
Q9Y2D5	Q9Y2D5	AKAP2_HUMAN	AKAP2	5840.32	3152.58	6221.69	3859.14	3624.46	5782.35		1568.27	
Q9Y2G0	Q9Y2G0	EFR3B_HUMAN	EFR3B							1699.75		1496.39
Q9Y2H1	Q9Y2H1	ST38L_HUMAN	STK38L			36237.4	6466.47		1626.9	7890.27	11864.7	9493.58
Q9Y2I6	Q9Y2I6	NINL_HUMAN	NINL		16368.1	8602.21	21062.9	41051.5	28214.7	18957.5	12858.7	17067.3
Q9Y2J2	Q9Y2J2	E41L3_HUMAN	EPB41L3				3138.09	2371.61	3478.72	1477.43		1274.69
Q9Y2K3	Q9Y2K3	MYH15_HUMAN	MYH15	20232.6	19024.2	13011.2	77631.6	76174.3	51171	9342.63		8671.16
Q9Y2K7	Q9Y2K7	KDM2A_HUMAN	KDM2A				2771.64			4489.8		2285.5
Q9Y2L1	Q9Y2L1	RRP44_HUMAN	DIS3				916.9	2097.42	2793.1	2561.4	1485.57	1991.79
Q9Y2Q3	Q9Y2Q3	GSTK1_HUMAN	GSTK1	6781.97	3222.24	4520.07	2019.85	1729.9	2836.49	5276.42	4539.63	3919.87
Q9Y2Q5	Q9Y2Q5	LTOR2_HUMAN	LAMTOR2					2093.41	1046.98	1830.82		563.203
Q9Y2R9	Q9Y2R9	RT07_HUMAN	MRPS7	6300.71	9080.21	7298.93	3928.55	6012.69	3617.69	8442.23	5284.09	9710.43
Q9Y2S7	Q9Y2S7	PDIP2_HUMAN	POLDIP2	2308.99			3925.54	3256.44	4495.12	3668.92	3254.79	4732.68
Q9Y2T2	Q9Y2T2	AP3M1_HUMAN	AP3M1	9613.61	9782.19	5531.18	5843	12160.1	7481.26	7254.46	7210.06	8455.05
Q9Y2U5	Q9Y2U5	M3K2_HUMAN	MAP3K2	28585.9	27609.2		13533.2	7056.61	10465.6		11462.3	29152
Q9Y2V2	Q9Y2V2	CHSP1_HUMAN	CARHSP1			340.101	253.131		1368.7			
Q9Y2V7	Q9Y2V7	COG6_HUMAN	COG6				1311.85		1752.93	1688.19	1567.34	1999.69
Q9Y2W1	Q9Y2W1	TR150_HUMAN	THRAP3	2724.23	3400.07			2442.76	1054.57	1093.06		
Q9Y2W2	Q9Y2W2	WBP11_HUMAN	WBP11					9746.73	8611.56	4753.15		3308.85
Q9Y2W6	Q9Y2W6	TDRKH_HUMAN	TDRKH						539.065	1335.63	238.963	1398.97
Q9Y2X0	Q9Y2X0	MED16_HUMAN	MED16	1921.47	4358.37	2943.86	3239.13	2211.12	2249.03	4504.9	3752.91	4689.66
Q9Y2X3	Q9Y2X3	NOP58_HUMAN	NOP58				1860.79	3186.56	2243.37	1803.45	1258.63	1842.26
Q9Y2X7	Q9Y2X7	GIT1_HUMAN	GIT1			2420.56	3618.7		859.389	4994.3	2159.68	5506.87
Q9Y2X9	Q9Y2X9	ZN281_HUMAN	ZNF281					1583.09	384.53			
Q9Y2Y1	Q9Y2Y1	RPC10_HUMAN	POLR3K									
Q9Y2Y9	Q9Y2Y9	KLF13_HUMAN	KLF13									
Q9Y2Z0	Q9Y2Z0	SGT1_HUMAN	SUGT1				1442.55	978.455	2154.56	1597.65		1518.4
Q9Y2Z2	Q9Y2Z2	MTO1_HUMAN	MTO1	4514.3			1600.6	2732.6	1400.27	3350.61	2908.7	3845.82
Q9Y305	Q9Y305	ACOT9_HUMAN	ACOT9	11435.4	10912.8	10629.2	12346	8013.95	5598.11	10193.2	17720.8	10973.2
Q9Y312	Q9Y312	AAR2_HUMAN	AAR2		1479.37			2152.37	1056.21	2051.05	2911.42	3442.54
Q9Y314	Q9Y314	NOSIP_HUMAN	NOSIP					3108.31	2782.14	944.047		1505.94
Q9Y315	Q9Y315	DEOC_HUMAN	DERA		3282.74	4386.15	4639.05	2736.5	3938.76	6292.66	4977.68	5614.28
Q9Y320	Q9Y320	TMX2_HUMAN	TMX2		2013.89		1787.2	4464.05	4649.97	7142.75	2427.46	3511.37
Q9Y371	Q9Y371	SHLB1_HUMAN	SH3GLB1				1976.87	2625.76	3271.04	1805.07		2424.3
Q9Y383	Q9Y383	LC7L2_HUMAN	LUC7L2				1941.62	1832.52	1180.23		2501.74	1620.77
Q9Y3B3	Q9Y3B3	TMED7_HUMAN	TMED7				3893.55	6685.2	10550.1	9019.2	3603.45	8140.94
Q9Y3B4	Q9Y3B4	SF3B6_HUMAN	SF3B6	1499.74	2805.49			1253.44	1045.49		1574.8	917.339
Q9Y3B7	Q9Y3B7	RM11_HUMAN	MRPL11	6234.1	7113.32	5935.23	3289.71	6177.46	3655.84	3641.89	6703.93	3380.07
Q9Y3C1	Q9Y3C1	NOP16_HUMAN	NOP16			4506.3		22743.4	26777.4			
Q9Y3C6	Q9Y3C6	PPIL1_HUMAN	PPIL1			11773.9	2161.8	2099.68	2735.04			2216.14
Q9Y3D0	Q9Y3D0	CIA2B_HUMAN	CIAO2B				1578.22	1256.53	2813.93	2930.61	2134.12	4242.52
Q9Y3D3	Q9Y3D3	RT16_HUMAN	MRPS16	1560.05	4280.33	1555.32	1005.38	1130.64	1419.17	2276.95	1304.56	2937.66
Q9Y3D6	Q9Y3D6	FIS1_HUMAN	FIS1					1866.2	1026.96	1956.18	1262.8	2518.72
Q9Y3D8	Q9Y3D8	KAD6_HUMAN	AK6					2839.57	1668.62	2125.36		3698.28
Q9Y3D9	Q9Y3D9	RT23_HUMAN	MRPS23	7047.49		1948.76	3899.9	2268.91	2759.99	4180.69	5851.94	4152.03
Q9Y3E5	Q9Y3E5	PTH2_HUMAN	PTRH2		800.468			2331.74	1720.02	823.142		1043.39
Q9Y3F4	Q9Y3F4	STRAP_HUMAN	STRAP	1285.12		2101.82	2088.1	2797.01	6302.54	4140.27	1282.39	3758.92
Q9Y3I0	Q9Y3I0	RTCB_HUMAN	RTCB	8943.79	3561.84	5831.44	8719.69	15102.9	10090.4	9293.14	7702.14	9708.99
Q9Y3I1	Q9Y3I1	FBX7_HUMAN	FBXO7				2695.01	2017.56	1868.88	1587.53	1909.76	2535.13
Q9Y3L5	Q9Y3L5	RAP2C_HUMAN	RAP2C				3851.94	3858.46	4953.45	14987.7	3706.16	14296.2
Q9Y3M8	Q9Y3M8	STA13_HUMAN	STARD13	2310.26	1312.76	915.518	542.602	594.641	632.68			120.595
Q9Y3R4	Q9Y3R4	NEUR2_HUMAN	NEU2									
Q9Y3R5	Q9Y3R5	DOP2_HUMAN	DOP1B				593.452	1951.53	1711.02	2027.09		637.394
Q9Y3T6	Q9Y3T6	R3HC1_HUMAN	R3HCC1				5739.24	3228.21	4806.43	4427.81	5963.51	4393.46
Q9Y3U8	Q9Y3U8	RL36_HUMAN	RPL36	12027.6	17504.1	22125.7	24563.7	45466.6	17364.6	24407.5	24897	25236.8
Q9Y3Z3	Q9Y3Z3	SAMH1_HUMAN	SAMHD1	24260.2	21938.4	18536.3	14488.9	11579.8	10395.6	13962.4	25554.1	18648.7
Q9Y421	Q9Y421	FA32A_HUMAN	FAM32A				3128.09	3576.62	2996.96		2572.54	2628.47
Q9Y446	Q9Y446	PKP3_HUMAN	PKP3				6247.81	10626.8	4978.52	8096.58	8097.72	8985.41
Q9Y467	Q9Y467	SALL2_HUMAN	SALL2					441.493	335.667			
Q9Y487	Q9Y487	VPP2_HUMAN	ATP6V0A2					5245.22	1966.85	2639.28		1488.86
Q9Y490	Q9Y490	TLN1_HUMAN	TLN1	13751.4	11348.5	11526.7	13811.7	10540.8	12570	6707.58	5482.43	7221.61
Q9Y4B5	Q9Y4B5	MTCL1_HUMAN	MTCL1				4613.06	6145.49	2960.31	1055.94	1295.08	1227.86
Q9Y4C2	Q9Y4C2	TCAF1_HUMAN	TCAF1			3307.99	2852.86	5123.37	3130.15	5319.74	6517.24	8256.6
Q9Y4D1	Q9Y4D1	DAAM1_HUMAN	DAAM1			3495.16	1745.25	3851.99	3041.07	3361.63	1491.82	2359.61
Q9Y4F1	Q9Y4F1	FARP1_HUMAN	FARP1	9762.59	6198.98	5587.4	2061.86		3936.93	14556.5	5327.33	16003.1
Q9Y4F3	Q9Y4F3	MARF1_HUMAN	MARF1					3130.73	1194.65	832.133		
Q9Y4H2	Q9Y4H2	IRS2_HUMAN	IRS2	11055	8018.8	9236.06	6364.6	5899.15	4555.08	1470.75	1297.47	2027.29
Q9Y4I1	Q9Y4I1	MYO5A_HUMAN	MYO5A	12207	13103.7		22765.3	74388.2	36084.1	38025.6	23844.7	35136.2
Q9Y4K0	Q9Y4K0	LOXL2_HUMAN	LOXL2					6159.81	5089.15			
Q9Y4L1	Q9Y4L1	HYOU1_HUMAN	HYOU1	5167.94	2536.06	5848.74	9065.3	9491.88	14428.2	7728.3	3955.16	7849.47
Q9Y4P3	Q9Y4P3	TBL2_HUMAN	TBL2							1856.37	1918.58	1345.2
Q9Y4R8	Q9Y4R8	TELO2_HUMAN	TELO2	1335.58	1029.78	975.307	7001.4	4979.54	4409.37	4682.33	4984.95	6524.85
Q9Y4W6	Q9Y4W6	AFG32_HUMAN	AFG3L2				2402.11	2483.52	3881.95	3967.75	3281.22	5804.1
Q9Y4X0	Q9Y4X0	AMMR1_HUMAN	AMMECR1					2343.92	2018.96	2346.97		2436.3
Q9Y4X4	Q9Y4X4	KLF12_HUMAN	KLF12									
Q9Y4Z0	Q9Y4Z0	LSM4_HUMAN	LSM 4.00					1782.36	2082.04	1282.49		1526.3
Q9Y512	Q9Y512	SAM50_HUMAN	SAMM50	42610.5	38171.9	32078.3		11210.2	4988.79	10852.4		10234.1
Q9Y520	Q9Y520	PRC2C_HUMAN	PRRC2C	2595.11	1469.8	1874.5	2539.93	5123.59	3231.54	2266.85	1709.48	1892.09
Q9Y570	Q9Y570	PPME1_HUMAN	PPME1				1539.06	2426.76	3438.94	1360.52		
Q9Y5A7	Q9Y5A7	NUB1_HUMAN	NUB1			1006.47	1221.54	1012.22	866.119	1688.85	787.494	1622.49
Q9Y5A9	Q9Y5A9	YTHD2_HUMAN	YTHDF2					10041.3	4951.46		2936.75	6196.53
Q9Y5B9	Q9Y5B9	SP16H_HUMAN	SUPT16H	7688.07	7586.79	4196.91	6899.18	7934.53	4644.4	5828.99	5102.44	6281.67
Q9Y5K5	Q9Y5K5	UCHL5_HUMAN	UCHL5				3166.35	5075.76	5196.39	2370.42	2512.56	2108.18
Q9Y5K6	Q9Y5K6	CD2AP_HUMAN	CD2AP				4405.32	4781.6	8327.58	2621.36	2138.5	3433.91
Q9Y5K8	Q9Y5K8	VATD_HUMAN	ATP6V1D	3268.21	4357.03	5806.96	2461.91	5847.89	5601.81	3870.87		4006.76
Q9Y5L0	Q9Y5L0	TNPO3_HUMAN	TNPO3	5643.74	5781.85	7254.99	7307.33	9049.16	3905.26	10109.4	8541.25	11174.7
Q9Y5L4	Q9Y5L4	TIM13_HUMAN	TIMM13	3539.95	2215.28	2285.56	5824.61	9249.12	12656	10753.2	5364.76	8332.6
Q9Y5M8	Q9Y5M8	SRPRB_HUMAN	SRPRB	2630.85	2860.65	3431.65	7030.48	4266.21	2917.45	7493.56	8484.68	6348.03
Q9Y5P6	Q9Y5P6	GMPPB_HUMAN	GMPPB				3247.25				4641.69	
Q9Y5Q8	Q9Y5Q8	TFIC5_HUMAN	GTF3C5	2839.9			2396.76	2191.21	3172.07	4027.04	3318.54	4658.96
Q9Y5Q9	Q9Y5Q9	TFIC3_HUMAN	GTF3C3		2514.01		1610.08	2373.19	2613.34	3964.44	1815.91	3562.61
Q9Y5S1	Q9Y5S1	TRPV2_HUMAN	TRPV2				5280.08	5407.44	4485.99	3078.04		4664.61
Q9Y5S2	Q9Y5S2;Q5VT25	MRCKB_HUMAN	CDC42BPB	4847.7	6992.82	3500.88	3185.18	3510.85	3502.57	4279.08	2506.56	4458.2
Q9Y5V3	Q9Y5V3	MAGD1_HUMAN	MAGED1	5394.87	4328.76	5491.35	3948.93	3066.4	2084.77	5433.26	9866.1	5428.84
Q9Y5X1	Q9Y5X1	SNX9_HUMAN	SNX9	6949.96		6117.23	4555.8	3330.73	5008.74	2236.33		3415.18
Q9Y5X2	Q9Y5X2	SNX8_HUMAN	SNX8	3900.85	3877.98	4161.23	4205.45	963.974	1942.37	2090.42	2185.92	2441.48
Q9Y5X3	Q9Y5X3	SNX5_HUMAN	SNX5						2117.08	1013.6		591.94
Q9Y5Y0	Q9Y5Y0	FLVC1_HUMAN	FLVCR1					760.36	843.826	2518.24		1605.85
Q9Y605	Q9Y605	MOFA1_HUMAN	MRFAP1		1904.01	1270.34	546.726	1211.35	2309.29	1261.86		1684.6
Q9Y606	Q9Y606	PUS1_HUMAN	PUS1			1708.01	2691.67		4086.14	5149.34	2475.42	5718.24
Q9Y613	Q9Y613	FHOD1_HUMAN	FHOD1				2322.43	6635.03	4919.48	2273.51	2457.63	2068.97
Q9Y617	Q9Y617	SERC_HUMAN	PSAT1	7165.64	4404.86	5658.77	10977.9	8106.15	16824.6	6219.96	4134.23	4675.23
Q9Y618	Q9Y618	NCOR2_HUMAN	NCOR2			2207.19	1629.59	3615.15	3943.71	3494.45	1522.26	4756.3
Q9Y624	Q9Y624	JAM1_HUMAN	F11R				2411.05	4858.04	4265.44	12113.4		9789.83
Q9Y639	Q9Y639	NPTN_HUMAN	NPTN	4336.58	4330.74	5344.54	3445.68	7652.43	5894.82	2733.48		2396.99
Q9Y666	Q9Y666	S12A7_HUMAN	SLC12A7							3103.98		3910.29
Q9Y673	Q9Y673	ALG5_HUMAN	ALG5				3813.07				4101.68	
Q9Y678	Q9Y678	COPG1_HUMAN	COPG1	9048.11	8040.86	10173.4	14109.9	9358.75	8234.1	9619.59	9299.48	9999.04
Q9Y679	Q9Y679	AUP1_HUMAN	AUP1	1423.18	1781.52	1428.37	3301.65	3369.47	4305.25	7517.77	1777.87	8013.77
Q9Y696	Q9Y696	CLIC4_HUMAN	CLIC4	3792.44	3326.09	4914.65	2906.13		2062.41			
Q9Y697	Q9Y697	NFS1_HUMAN	NFS1			2452.88	2488.88	2652.46	2153.98	2744.23	3942.03	3779.65
Q9Y6C9	Q9Y6C9	MTCH2_HUMAN	MTCH2		11592	12495.1		5119.58	3569.55	8249.47	3175.69	7830.89
Q9Y6D5	Q9Y6D6;Q9Y6D5	BIG2_HUMAN	ARFGEF2	1545.14			4797.14	4520.7	3787.31	3974.47	5132.93	3969.94
Q9Y6D6	Q9Y6D6	BIG1_HUMAN	ARFGEF1	3134.44	4240.44	2559.52	3737.56	2389.08	3079.34	3366.39	2673.54	3956.51
Q9Y6E0	O00506;Q9Y6E0	STK24_HUMAN	STK24	1920.11	1519.32	2097.96	1568.11	8951.96	3479.92	3215.27	1216.42	3107.89
Q9Y6E2	Q9Y6E2	5MP1_HUMAN	BZW2	6109.25	4211.44	3611.5	7218.49	6166.45	7899.03	9246.45	4615.23	8711.47
Q9Y6G9	Q9Y6G9	DC1L1_HUMAN	DYNC1LI1	4247.42	6253.87	8098.47	10680.3	7540.7	6344.05	6827.38	9644.69	7621.88
Q9Y6J9	Q9Y6J9	TAF6L_HUMAN	TAF6L	4816.23	3485.18	3797.1	1854.3	7140.06		6819.49	4282.91	7913.76
Q9Y6K0	Q9Y6K0	CEPT1_HUMAN	CEPT1	7533.7	7772.49	4925.49	5224.65	5760.63	3812.93	11895.1	7110.08	15948.5
Q9Y6K5	Q9Y6K5	OAS3_HUMAN	OAS3	4452.85	3324.69	3728.21	2640.99	4421.12	1904.95	2536.63	11576.9	3496.62
Q9Y6K8	Q9Y6K8	KAD5_HUMAN	AK5	5301.86	2416.8	5796.63						
Q9Y6M1	Q9Y6M1	IF2B2_HUMAN	IGF2BP2	9441.43	6780.44	7037.14	4915.4	8831.42	3929.85	2976.58	3321.82	2558.44
Q9Y6M4	Q9Y6M4;P78368;Q9HCP0	KC1G3_HUMAN	CSNK1G3				6312.54	12541.8	11647.8	38637.1	9624.57	29625.9
Q9Y6M7	Q9Y6M7	S4A7_HUMAN	SLC4A7					1049.67	414.339	1065.33		1869.15
Q9Y6N5	Q9Y6N5	SQOR_HUMAN	SQOR	41651.9	31822.8	42817				11606.8	16047.3	14247.7
Q9Y6N7	Q9Y6N7	ROBO1_HUMAN	ROBO1	1408.06		1096.86		973.143	1178.54	2372.51	1203.91	2092.39
Q9Y6Q1	Q9Y6Q1	CAN6_HUMAN	CAPN6							2302.61		2206.29
Q9Y6Q9	Q9Y6Q9	NCOA3_HUMAN	NCOA3									
Q9Y6R9	Q9Y6R9	CCD61_HUMAN	CCDC61	10653.8	9698.03	9733.35			2092.17			
Q9Y6V7	Q9Y6V7	DDX49_HUMAN	DDX49				1599.43	5535.06	4363.02	3355.87	4511.82	3484.61
Q9Y6W5	Q9Y6W5	WASF2_HUMAN	WASF2				4532.58	1531.43	2501.08	614.923		923.282
Q9Y6X9	Q9Y6X9	MORC2_HUMAN	MORC2				686.516	675.432		1065.59	276.819	1092.7
Q9Y6Y8	Q9Y6Y8	S23IP_HUMAN	SEC23IP	4454.89		3227.22	3952.03	10427.3	11092.1	6359.16	1739.71	5245.94
